# A Geometric Definition of Short to Medium Range Hydrogen-Mediated Interactions in Proteins

**DOI:** 10.3390/molecules25225326

**Published:** 2020-11-15

**Authors:** Matthew Merski, Jakub Skrzeczkowski, Jennifer K. Roth, Maria W. Górna

**Affiliations:** 1 Biological and Chemical Research Centre, Department of Chemistry, University of Warsaw, 02-089 Warsaw, Poland; jakub.skrzeczkowski@student.uw.edu.pl; 2 Department of Psychology, Carlow University, Pittsburgh, PA 15213, USA; jkroth@carlow.edu

**Keywords:** hydrogen bond, CH-π bond, protein crystal structure, methionine, protein geometry

## Abstract

We present a method to rapidly identify hydrogen-mediated interactions in proteins (e.g., hydrogen bonds, hydrogen bonds, water-mediated hydrogen bonds, salt bridges, and aromatic π-hydrogen interactions) through heavy atom geometry alone, that is, without needing to explicitly determine hydrogen atom positions using either experimental or theoretical methods. By including specific real (or virtual) partner atoms as defined by the atom type of both the donor and acceptor heavy atoms, a set of unique angles can be rapidly calculated. By comparing the distance between the donor and the acceptor and these unique angles to the statistical preferences observed in the Protein Data Bank (PDB), we were able to identify a set of conserved geometries (15 for donor atoms and 7 for acceptor atoms) for hydrogen-mediated interactions in proteins. This set of identified interactions includes every polar atom type present in the Protein Data Bank except OE1 (glutamate/glutamine sidechain) and a clear geometric preference for the methionine sulfur atom (SD) to act as a hydrogen bond acceptor. This method could be readily applied to protein design efforts.

## 1. Introduction

Hydrogen atoms mediate many of the important interactions involved in protein structure and function. One major type of these interactions is made up of canonical hydrogen bonds, defined as a set of interactions between a hydrogen atom bound to a pair of electronegative atoms in which there is evidence of a bond between the hydrogen and both atoms [1]. These bonds are dominated by simple electrostatic terms, but also include contributions from higher-order electrostatic interactions (dipoles, quadrupoles, etc.), π-interactions, dispersion, and charge transfer terms [1,2,3,4,5,6,7,8]. In proteins, hydrogen bonds between strongly electronegative nitrogen amine and carbonyl oxygen define the α-helical and β-sheet secondary structure elements [6,9,10] essential to forming compact folded structures [11]. Similarly, oxygen [12,13] and nitrogen [14] atoms present in the amino acid side chains also tend to be involved in canonical hydrogen bonding interactions with water molecules [15] and other electronegative atoms present in ligands [16,17], as well as in the protein itself, involving both the backbone [9] and sidechains [10] (including sulfur atoms in cysteine and methionine [18]). Sulfur is a large, soft atom, unlike the smaller, harder oxygen and nitrogen atoms, and these chemical differences mean that hydrogen bonds formed by sulfur atoms typically have greater contributions from the higher-order electrostatic terms, dispersive interactions, and charge transfer terms and are less well defined by traditional definitions of hydrogen bonding [18]. These issues become even more pressing when even less traditional hydrogen bonding-type interactions are subject of interest, such as hydrogen bonding from carbon donors (CH) [19] or to π-cloud acceptors [20,21,22] including those of aromatic ring systems (Ar-H) in phenylalanine, tyrosine, histidine, and tryptophan [13] as well as the guanidinium moiety of arginine [23].

While issues involving non-canonical hydrogen bonds can be dealt with both theoretically [24,25] and experimentally [22,26,27] when studying small molecules, this is generally not currently possible for proteins [27]. Instead, treatments of hydrogen bonding interactions in proteins typically utilize a simplified electrostatic model [9,28], which requires estimation of the distances between the hydrogen atom and the two electronegative atoms [9]. Additionally, for many such interactions, the positional error of the hydrogen atom is negligible when compared to the experimental positional uncertainty of the heavy atoms in the protein, especially those in which the pK values of the donor and acceptor are well defined [29] and the structures are quite rigid. However, the environment in protein structures often perturbs pK values [30,31] and such phenomena are quite common in the active sites of enzymes and other areas of biochemical interest [32,33]. Additionally, even in well-ordered catalytic and ligand binding sites, atoms can exist in several conformations due to side chain rotations [34] and backbone flexibility [35]. Even where there is no evidence for any such flexibility, there are often complex networks of hydrogen bonds present in proteins which often involve multiple partnered electronegative atoms, thus making the position of the hydrogen atom in these networks ambiguous [36,37], especially-so for side chains with significantly rotatable hydrogen positions such as the lysine amine and serine hydroxyl [29]. These rotations often confound automatic hydrogen placement algorithms; a survey of 28 automated hydrogen prediction methods found poor agreement with experimental data with a median error of 0.76 Å for hydroxyl hydrogens [29]. Such errors are often obvious and can constitute a significant obstacle for research utilizing protein crystal structures, such as molecular docking [38].

**Figure 1:**
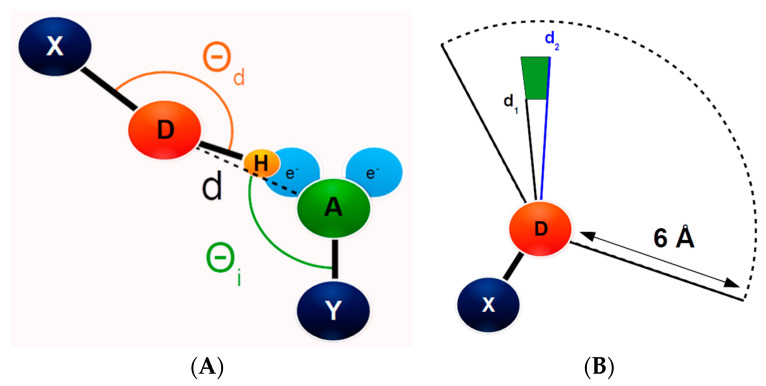
Graphical illustration of the geometric definition of hydrogen-mediated interactions (HMI). (A) Illustration of the heavy atoms which are included in the HMI definition including the two electronegative atoms (hydrogen atom donor D and hydrogen atom acceptor A) and their partners, X and Y, respectively. The distance between D and A is illustrated as distance d. The direct angle between X, D, and A (Θ_d_, orange) and the indirect angle made up of points Y, A, and D (Θ_i_, green) are also illustrated. (B) Demonstrative side view showing the annular shape of the volume of each bin in space. Each bin is 0.1 Å and 1° between d_1_ and d_2_ so a cone correction is applied to compensate for the greater volume of the longer distance bins.

However, even when high-quality experimental data is available, such as from atomic (better than 1 Å) resolution crystal structures or neutron diffraction data, many hydrogen atoms are still poorly localized, especially for side chain atoms in which the hydrogen atom has rotational freedom [29]. While these methods (and NMR) are capable of producing experimental evidence for hydrogen atoms, to date, the vast majority of protein structural information has been collected using x-ray crystallography (with a gradually increasing average resolution) [39]. To address this problem, we sought out a method that could investigate these kinds of interactions and utilize the large amount of available protein crystallographic data. We used a geometric definition that relies solely on the position of the non-hydrogen heavy atoms and relies on the basic principle that energetically favorable interactions will tend to be statistically overrepresented among all possible geometric conformations, a principle that is recognized in the IUPAC definition of hydrogen bonding [1]. Because the definition introduced here relies only on heavy atom geometry (Figure 1), we could not only look at traditional hydrogen bonds but also non-canonical interactions such as CH-π bonds [21]. While the existence of these bonds is not without controversy, we also acknowledge that lacking explicit detail on the position of a hydrogen atom makes it difficult to differentiate hydrogen bonding and salt bridge interactions [3,11,40], so we refer to all of these collectively (e.g., canonical hydrogen bonds [1,9], water-mediated hydrogen bonds [15], salt bridges [8,11,28], Ar-H [19,21,22], and ion-π [41] interactions) as hydrogen-mediated interactions (HMI). By examining high-resolution crystal structures using this method, we were able to identify at least one favored geometry for all the expected donor atom types in the Protein Data Bank (PDB) [39] and produce a simple method to estimate HMI quality in current and future protein structures that is readily applicable to side chain interactions and does not necessitate the use of experimental or predicted hydrogen atoms but rather utilizes the much more experimentally accessible heavy atom geometry.

## 2. Results

Searching for favored geometries identifies hydrogen-mediated interactions. By searching for geometrically favored relationships between hydrogen, donor, and acceptor atoms, we were able to readily and easily identify hydrogen-mediated interactions in proteins. This process identified 68 geometrical interactions around hydrogen-donating atoms and 55 centered on hydrogen-accepting atoms which surpassed the appropriate threshold volume (Tables S1–S3). Of these, those that involved very short (<2.5 Å) interaction distances between the donor and acceptor atoms and were eliminated. For example, the major interaction for the cysteine sulfur (SG) was very short (2.0 Å) and at an acute angle when analyzed as either a donor or acceptor, suggesting a covalent disulfide bond rather than an HMI. For donor-defined interactions, the largest peak (as defined by total cluster volume) was associated with interactions of the backbone nitrogen involved in the formation of a secondary structure in both α-helices and β-sheets. These 55 include at least one (and usually only one) interaction for all the expected atom types as well as a few non-traditional bonds including 38 distinct, possible CH donor interactions (Tables S1–S3), several of which are centered on short distances between the CH atom and backbone nitrogen atoms on the same (CG1 and one CB) or backbone atoms on neighboring residues (CA, CB, CG). These HMI tend to be short (2.4 Å) for CA and CB, with narrow (<110°) angles (13 of 38 (34%) CH donors). There are two major exceptions to the one-to-one correlation between atom types and HMI: the atom type NE2 (which is chemically different when found in histidine and glutamine) and OH (tyrosine hydroxyl). For the NE2 interactions, both the nearly linear (180°) histidine-based and the planar (120°) amine interaction geometry of glutamine can both be visually identified in the NE2 heat map (Figure S1). The other special case, the tyrosine OH atom, is clearly defined as a single-interaction cluster when measured from the direct angle but rather has two distinct clusters when measured from the indirect angle. By eliminating those possible HMI which are either too short, do not meet the appropriate volume threshold, or lack an equivalent interaction in the indirect angle, 15 identified HMI remain (Table 1). All 15 of these HMI can be readily identified as canonical hydrogen bonding interactions (Figure S2). Among these canonical 15, the average distance determined using both the direct and indirect angle measures were identical to within the same distance bin (0.1 Å) for 10 of the 15 (67%) HMIs, with only one varying by more than 0.1 Å (ND1). Notably, the tyrosine OH atom type has two HMI when measured from the indirect angle, one of which matches the direct angle distance. Altogether, this gives an overall good distance match between the identified interactions given the size of the bins and the expected spatial positioning error for protein structures of 2.0 Å resolution of 0.3 Å [42].

On the other hand, when acceptor atoms were analyzed, 55 putative interactions were identified. Using the previously discussed criteria (volume, interaction distance, and indirect interactions) quickly reduced this to a set of 8 interaction geometries. However, one of these 8, CD1, is chemically forbidden from acting as an HMI acceptor and was also eliminated, leaving 7 identified HMI (Table S1, Figure S2). Much as with the donors, the major class (backbone O) was associated with secondary structure interactions, as expected (Table 1). Of the detected interactions, 6 of 7 (86%) had matching distance lengths in both the direct and indirect angles, although this time, one interaction (OH) differed by 0.3 Å. (It should be noted here that the geometries for OH both as a donor and an acceptor were identical.) In general, the acceptor-defined interactions had smaller average cluster volumes (385) than the donors (966) when accounting from the direct orientation. Most of the oxygen atom types will be good acceptors; OD1, OD2, and OE2 of glutamine, glutamate, asparagine, and aspartate side chains will likely be not protonated at physiological pH (even allowing for side chain flipping), while the tyrosine OH atom, which is typically singly protonated under physiological conditions and thus can be a functional acceptor. Furthermore, the non-traditional acceptor of the methionine sulfur atom (SD) was clearly identified to have a preferred HMI geometry, having the 4th largest cluster volume of all the acceptor HMIs. On the other hand, it also had a much longer HMI distance (3.8 Å) and a broad angle bin range (Q25–Q75 = 16 bins direct angle, 23 bins indirect angle). SD also tends to be involved in interactions with CH atom donors; 89.5% of all donor atom partners in its center of mass bin (3.8 Å, 140°, direct) are carbon atoms.

The existence of many of the putative HMIs examined were not statistically supported by the data. Two of the identified acceptor atom type interactions were problematic from a chemical point of view. The CE1-acceptor HMI likely arose from the method’s flexibility in identifying donors and acceptors due to ring flipping, while the ND1 atom type as an acceptor also likely arose from possible side chain flipping. Additionally notable, the OE1 atom type did not have any statistically preferred acceptor HMI. Another 20 of 55 (36%) putative HMI were centered on nitrogen atoms, which generally had weaker HMI identifiers as acceptors than donors. Identification of these as acceptors appeared to arise from atom types that were dually classed as donors and acceptors. Ten of these 20 putative (50%) nitrogen acceptor HMIs had direct angles more acute than 110°, while a further 4 of 20 (20%) had nearly linear (>170°) direct angles, making many of these putative nitrogen acceptor HMIs likely incidental contacts regardless of chemical concerns. The two acceptor HMIs that were identified but may be concerning (CE1 and ND1) both are present in histidine side chains. None of the Ar-H HMIs had clusters of significant size in the indirect angle analysis. These bin clusters had low values and also (typically,5 of 10, 50%) close contacts (2.0–2.2 Å) with broad angle requirements (Q25–Q75 = 20–26 bins, average 22.4). Furthermore, there were no indirect angle clusters that met the significance thresholds required for an HMI (Tables S1–S3). In general, identification of hydrogen-mediated interactions using hydrogen accepting atoms was less well supported than those of donor atoms.

Discussion and Conclusions: Using this simple geometric definition, HMIs could be identified for donor atoms in the range of 2–4 Å without explicit experimental identification of hydrogen atoms (Figure S3). The discovered geometric preferences were generally narrow, both in terms of distance and angle range (Table 1). All of the statistically identified HMIs were also chemically reasonable [43]. Being able to detect these interactions indirectly, without the need to experimentally locate the hydrogen atoms including on sidechain atoms, allowed HMI analysis of a large number of protein [43], and in theory could be readily and efficiently applied to an empirical scoring function [44] as a novel method to retrospectively quantify HMIs in solved crystal structures and in theory, de novo protein designs [45]. This geometric method is quick and easy to calculate and utilizes the information that is readily available from protein crystal structures of reasonably high resolution while obviating the need to introduce either a calculated hydrogen atom and its concurrent positional uncertainties [29,34,46] or the need to rely on estimated pK_a_ values [30,31], and it allows the analysis of non-canonical acceptors such as Ar-H interactions [19,21] which can be notoriously problematic to estimate in protein structures [5,28,47,48]. This method provides at least one optimal geometry for each of the donor atom types present in the PDB and can differentiate the chemically different forms of most PDB atom types.

In this work, we focused on the aspects of hydrogen bonding using the formal IUPAC definition, that are applicable to proteins [1]. Due to the size of proteins and their complexity, this is mainly the inherently directional nature of these interactions [49] that distinguishes HMI from isotropic (non-directional) interactions. Hydrophobic (Van der Waals), dispersive (London), and steric (6/12 potentials) are isotropic while hydrogen bonding, Ar-H [19], and salt bridge interactions [4,40,50], all of which we are including as HMIs, are directional [5,46]). Because we analyzed distances between 2 and 6 Å, we were able to largely obviate the need for a specific distance metric, thereby avoiding some controversies in this area as this length regime includes everything from long covalent disulfide bonds to water-mediated hydrogen bonds [15]. The ability to examine this entire distance range was doubly important for Ar-H interactions, which are typically considered to be longer than canonical hydrogen bonds (3–6 Å) [5,41,51]. However, only one (the six-membered ring of tryptophan) of the putative aromatic π-interactions had a greater interaction distance than 3.5 Å, and the angle of interaction was acute, to the main chain atoms of the residue itself (Table S3). The range of angles found for aromatic π-interactions was also quite wide (mean = 11.6˚, Table S3) [25]. In general, we did not find good evidence for a statistical predisposition towards anisotropic HMI for non-canonical atomic pairs, with the exception of the ability of the methionine SD atom to function as an HMI acceptor.

It should be noted that removing the hydrogen atom from the place of central importance and the somewhat arbitrary decisions used to choose the angular limits [21,43], as well as our somewhat unusual inclusion of hydrogens with free rotations [24], makes it difficult to compare interaction angles between this analysis of HMIs with other protein hydrogen bond analyses [2,4,7,52,53,54]. However, we note that we found no donor-centered interactions longer than the common distance limit of 3.5 Å [2,4,20,25,55,56] and only one acceptor interaction (Table 1) greater than that length despite measuring interactions out to 6 Å. It should neither escape notice that a large number of longer-distance CH-O interactions (average distance = 3.3 Å) have been identified in proteins [57], and there is a notable bulge at this length in the carbonyl O heat map in our analysis (Figure S1). It should not be understated that there is a pressing need for analyses of non-canonical HMIs in proteins, which have been laboriously documented in protein x-ray [19,21,41,43,47] and neutron structures [26]. Because this method is more general than those that specifically investigated these interactions (possibly due to their relative rarity [41]), our inability to detect aromatic π-interactions in no way disproves their existence [13,21,58]. Nor should these results in anyway detract from the power of the many widely used methods that deal with hydrogen placement in proteins which have long stood the test of time [9,39,52,53]. Rather, we propose this model as a way to extend the functionalities of those gold-standard methods.

Obviously, our method is not without its limitations. This model is simply too simple to differentiate hydrogen bonds from salt bridges, which tend to have only slightly shorter interaction distances [40,50], and we focus exclusively on intra-chain interactions [59]. It is also worth noting that the ambiguity present in several atom types (e.g., carboxylates of aspartate and glutamate, or histidine nitrogen atoms) necessitates ambiguity in donor and acceptor assignments, and also ambiguity in finding interactions between atoms which may in their natural state be matched donors or acceptors. More concerning is that this definition does not work particularly well to identify HMIs for hydrogen bond accepting atoms, including the π-clouds of aromatic residues. While many of the expected acceptor atom types do also have at least one well-defined, statistically preferred geometry (Table S3), as a whole, these HMIs are less well defined than those of donor atoms. Additionally, the atom type OE1 has a preferred donor HMI geometry but no acceptor geometries despite also being present in glutamine side chains (Table 1, Tables S1–S3). It should be noted that this method fell short of the original goal of modeling many if not all of the non-canonical HMIs such as those arising from carbon atom donors or π-cloud acceptors [19,21,41]. Part of this is undoubtedly due to the relative weakness of these bonds compared to canonical strong donor/strong acceptor pairs [5,19,20,21,41,51,57,60]. It is not unexpected that these weaker bonds would be weaker drivers of geometric preferences [5]. Additionally, the larger volume of space occupied by π-cloud acceptors [56,61] compared to singular atoms also likely dilutes any geometric preferences that might be detected as there is a much greater region of space and thus geometries with which a donor could interact with the π-electrons. Carbon atom donors do not have this problem, but many of the statistical preferences are overwhelmed by steric contacts around the backbone (Table S3, Figure S1). It should not be expected that these geometric preferences can directly be applied to an energy function, but rather function more as a quality check external to the energy function itself [14,48].

However, these short coming should not distract from the fact that by simply including a pair of partner atoms, it is possible to identify a set of conserved, optimal geometries for the most common donor-atom-based HMI in protein crystals. These geometrical constraints provide a quick method to identify likely HMI in protein atoms using the information that is readily available from protein crystal structures, which have potential applications in error-checking both actual protein experimental structures and de novo designed theoretical models.

Materials and Methods: The entire PDB database [39] was downloaded on 5 July 2016. The dataset was filtered to contain only those models with a resolution of 2 Å or better in order to focus on well-defined atomic positions. In order to minimize potential errors in the deposited models and to better account for statistical variation, the dataset was also limited to only those proteins which have been deposited at least ten times in the PDB (as defined by the PDB at 90% sequence identity). This was confirmed on a per residue basis by sequence alignment. In the end, these criteria resulted in 1874 groups of proteins, which contained a total of 49,549 protein chains. Water molecules, ligands, and other heteroatoms were not included in the analysis and each chain was analyzed individually so only intra-chain interactions were examined. Hydrogen-mediated interactions were identified as follows: each heavy atom (e.g., C, N, O, and S) in the protein chain that could be involved in an HMI according to its atom type (as defined by the atom name parameter in the PDB file) was classified either a donor (D), acceptor (A), or both based on its hydrogen bonding characteristics (Table S2). The atom naming convention was taken from the PDB; an illustration of this is included (Figure S4). Many atoms were classed as both donors and acceptors due to their equivocal chemical nature or common errors in identification in protein structures; the obvious example of this would be the CE1 atom of histidine, which may be a misidentified nitrogen atom or, due to natural ring flipping, be a nitrogen atom in some proportion of the protein structure. Each donor was associated with a partner atom (X) according to its atom type, and each acceptor with its equivalent partner (Y) as determined by its atom type, in order to properly orient the assumed hydrogen atoms (Figure 1). For many atom types, the partner atom was geometrically identical to a heavy atom covalently bonded to the original atom. However, for those atom types for which the partner atom could not be trivially placed, the positions of the appropriate dummy atom were defined by a geometric rule based on two or more additional atoms (Table S2). For example, the π-cloud of the phenyl ring of tyrosine was defined by a pair of points located 1 Å distant from a point located at the center of the ring as defined by the six carbon atoms of the side chain ring (CG, CD1, CD2, CE1, CE2, and CZ) and normal to the plane of the ring. One point was used for each face of the ring to make the pair of points; tryptophan has four such points due to its pair of rings. Then for each atom, the distance between that atom and every other atom in the protein chain was calculated and every atom within 6 Å, but at least 2 Å distance, was considered a potential HMI if that atom had a complementary classification to the query atom (i.e., “acceptor” or “both” for donors). Two angles were calculated from the four atoms in the potential HMI, XDA, and YAD, respectively (Figure 1A). Purpose-built code to analyze the HMIs present in PDB structures on a per chain basis and statistical correlation for a full data set is available online at https://gorna.uw.edu.pl/en/research/software. Analysis of the entire PDB took less than two weeks on a single desktop LINUX machine. A potential HMI was then considered to be an actual HMI if both angles were between 80° and 180°. The angle XDA is referred to as the direct angle for donors and the indirect angle for acceptors, while the opposite holds for the angle YAD. HMIs were analyzed in terms of both donor and acceptor characteristics (where applicable) and using both angles alternatively regardless of the atom characteristic.

All the HMIs detected for a given atom type were collected and divided into bins of 0.1 Å distance and 1° angle ranges. Bins were normalized for the total volume of the bin (cone correction) [62] (Figure 1B). The data were also normalized for atom frequency by dividing the total number of detected HMIs by the number of bins (*n* = 4000). An HMI was identified as a cluster of five or more contiguous bins that exceeded a threshold normalized value. Threshold values were employed to both reduce the amount of noise present in the data and to determine the limits of acceptable geometries for each HMI. The normalized value was established by empirical observation to separate legitimate signal peaks from stochastic noise. The bins themselves were arranged in a standard 2-D table and any bin in the eight surrounding a bin with at least a value of 3 that also had a value of at least 3 was considered a contiguous contact. For each identified HMI cluster, the bin in which the center-of-mass in each dimension resided was identified, along with the bins that defined the limits of the 25th and 75th quartiles (Table 1). For donor atoms, hydrogen-mediated interactions were verified by examining the sum total of the ratio values in the bins exceeding a threshold volume of 500 units (determined empirically by identifying narrow peaks in the heat maps) for the direct angle and at least 150 units for the indirect angle formulation. Identified bonds were further screened by requiring that an equivalent bond was also present in the other formulation and a minimum bond length of 2.5 Å was also measured (Tables S1–S3). For acceptor atoms, all the same held except the threshold was lowered to 150 units for the direct angle but raised to 200 for the indirect angle. Examination of the bonds identified in the PDB allowed categorization of the bond type.

## Figures and Tables

**Table 1:** Table of identified and confirmed HMI. The confirmed HMIs are listed according to donor or acceptor heavy atom identity with geometries given using either the direct or indirect angles. Atom types are listed in order of decreasing cluster volume (for the direct angle) and the average (by center of mass) distance and angle and the 1st and 3rd quartiles (25–75 range) are also given, as well as general information about the HMI.

**Donors, Direct**							
	**Atom**	**Distance**	**25–75 Range**	**Angle**	**25–75 Range**	**Volume**	**Notes**
**1**	N	2.9	2.8–3.1	171	166–176	2126	strand to strand/helix
**2**	NE	2.8	2.7–2.9	171	165–176	1678	standard bond
**3**	NE1	2.8	2.8–2.9	172	166–176	1659	standard bond (Trp)
**4**	ND1	2.7	2.7–2.8	173	168–177	1511	standard bond
**5**	OD1	2.8	2.8–2.9	136	124–151	839	standard bond
**6**	NH2	2.9	2.8–3.0	105	92–119	827	standard bond
**7**	OD2	2.8	2.7–2.8	125	118–133	780	standard bond
**8**	NZ	2.8	2.7–2.8	107	99–114	777	standard bond
**9**	NE2	2.7	2.7–2.8	173	169–177	766	His bond
**10**	ND2	2.9	2.8–2.9	120	114–126	719	standard bond
**11**	OH	2.6	2.6–2.7	115	112–119	610	standard bond
**12**	OE2	2.8	2.7–2.8	123	117–130	590	standard bond
**13**	NH1	2.8	2.8–2.9	132	127–136	576	standard bond
**14**	OG	2.7	2.6–2.8	113	105–120	529	standard bond
**15**	OG1	2.7	2.6–2.8	112	106–118	504	standard bond
**Donors, Indirect Indirect**							
	**Atom**	**Distance**	**25–75 Range**	**Angle**	**25–75 Range**	**Volume**	**Notes**
**1**	N	2.9	2.8–2.9	159	154–166	1197	strand to strand/helix
**2**	NE	2.8	2.7–2.8	135	120–148	349	standard bond
**3**	NE1	2.8	2.8–2.9	135	125–142	368	standard bond
**4**	ND1	2.9	2.8–3.0	170	166–176	353	standard bond
**5**	OD1	2.8	2.8–2.9	171	164–176	1414	standard bond
**6**	NH2	2.8	2.8–2.9	137	123–150	986	standard bond
**7**	OD2	2.8	2.7–2.9	173	168–177	1562	standard bond
**8**	NZ	2.8	2.7–2.8	148	134–163	1232	standard bond
**9**	NE2	2.8	2.8–2.9	141	132–154	601	standard bond(Gln and His (poor))
**10**	ND2	2.9	2.8–2.9	142	131–156	966	standard bond
**11**	OH	2.6	2.5–2.6	131	122–139	502	standard bond
**11a**	OH	2.8	2.7–2.9	174	169–177	577	standard bond
**12**	OE2	2.8	2.7–2.9	174	168–177	1334	standard bond
**13**	NH1	2.8	2.8–2.9	140	129–152	953	standard bond
**14**	OG	2.8	2.7–3.0	164	150–172	1239	standard bond
**15**	OG1	2.8	2.7–2.9	160	144–169	1234	standard bond
**Acceptors, Direct**							
	**Atom**	**Distance**	**25–75 Range**	**Angle**	**25–75 Range**	**Volume**	**Notes**
**1**	O	2.9	2.8–3.2	156	150–162	1073	strand to strand/helix
**2**	SD	3.8	3.8–3.9	140	132–147	439	CH-acceptor bond
**3**	OD2	2.8	2.7–2.8	124	119–132	324	standard bond
**4**	OD1	2.8	2.8–2.9	137	131–144	182	standard bond
**5**	OE2	2.8	2.7–2.8	123	119–127	160	standard bond
**6**	OH	2.6	2.5–2.6	115	113–118	154	standard bond
**7**	ND1	3.2	3.1–3.3	95	91–99	305	poor bond
**Acceptors, Indirect Indirect**							
	**Atom**	**Distance**	**25–75 Range**	**Angle**	**25–75 Range**	**Volume**	**Notes**
**1**	O	2.9	2.8–3.0	170	164–176	1478	strand to strand/helix
**2**	SD	3.8	3.7–4.0	163	152–174	856	CH-acceptor bond
**3**	OD2	2.8	2.7–2.9	173	168–177	1001	standard bond
**4**	OD1	2.8	2.8–2.9	171	164–176	958	standard bond
**5**	OE2	2.8	2.7–2.9	174	169–178	791	standard bond
**6**	OH	2.9	2.8–3.0	176	173–178	248	standard bond
**7**	ND1	3.2	3.1–3.3	112	109–115	236	poor bond

## Supplementary Materials

The following are available online, Table S1: Table of identified hydrogen mediated interactions identified in the PDB, Table S2: Table of amino acids and atom types present in the PDB, Table S3: Table identifying the partner atom for each analyzed atom (by type), Figure S1: Heat maps for each of the HMI, Figure S2: Example geometries for each of the HMI, Figure S3: Average geometries of the identified HMI, Figure S4: The 20 standard amino acids and their atom names from the PDB. Source code for the calculations performed in this work is available online at https://gorna.uw.edu.pl/en/research/software.

## References

[1] Arunan E., Desiraju G.R., Klein R.A., Sadlej J., Scheiner S., Alkorta I., Clary D.C., Crabtree R.H., Dannenberg J.J., Hobza P. (2011). Definition of the hydrogen bond (IUPAC Recommendations 2011). Pure Appl. Chem..

[2] Baker E.N., Hubbard R.E. (1984). Hydrogen-Bonding in Globular-Proteins. Prog. Biophys. Mol. Biol..

[3] Meot-Ner M. (2005). The ionic hydrogen bond. Chem. Rev..

[4] Bissantz C., Kuhn B., Stahl M. (2010). A Medicinal Chemist’s Guide to Molecular Interactions. J. Med. Chem..

[5] Burley S.K., Petsko G.A. (1988). Weakly Polar Interactions in Proteins. Adv. Protein Chem..

[6] Dill K.A. (1990). Dominant Forces in Protein Folding. Biochemistry.

[7] Fersht A.R., Shi J.P., Knill-Jones J., Lowe D.M., Wilkinson A.J., Blow D.M., Brick P., Carter P., Waye M.M., Winter G. (1985). Hydrogen bonding and biological specificity analysed by protein engineering. Nature.

[8] Hubbard R.E. (2001). Hydrogen Bonds in Proteins: Role and Strength. Encyclopedia of Life Sciences.

[9] Kabsch W., Sander C. (1983). Dictionary of protein secondary structure: Pattern recognition of hydrogen-bonded and geometrical features. Biopolymers.

[10] Garcia A.E., Sanbonmatsu K.Y. (2002). α-Helical stabilization by side chain shielding of backbone hydrogen bonds. Proc. Natl. Acad. Sci. USA.

[11] Vogt G., Woell S., Argos P. (1997). Protein thermal stability, hydrogen bonds, and ion pairs. J. Mol. Biol..

[12] Derewenda Z.S., Lee L., Derewenda U. (1995). The Occurrence of C–H···O Hydrogen-Bonds in Proteins. J. Mol. Biol..

[13] Dragelj J.L., Stankovic I.M., Bozinovski D.M., Meyer T., Veljkovic D.Z., Medakovic V.B., Knapp E.W., Zaric S.D. (2016). C-H/O Interactions of Aromatic CH Donors within Proteins: A Crystallographic Study. Cryst. Growth Des..

[14] Fleming P.J., Rose G.D. (2005). Do all backbone polar groups in proteins form hydrogen bonds?. Protein Sci..

[15] Levinson N.M., Boxer S.G. (2014). A Conserved Water-Mediated Hydrogen Bond Network Underlies Selectivity of the Kinase Inhibitor Bosutinib. Biophys. J..

[16] Boobbyer D.N.A., Goodford P.J., Mcwhinnie P.M., Wade R.C. (1989). New Hydrogen-Bond Potentials for Use in Determining Energetically Favorable Binding-Sites on Molecules of Known Structure. J. Med. Chem..

[17] Hamdane D., Bou-Nader C., Cornu D., Hui-Bon-Hoa G., Fontecave M. (2015). Flavin-Protein Complexes: Aromatic Stacking Assisted by a Hydrogen Bond. Biochemistry.

[18] Zhou P., Tian F.F., Lv F.L., Shang Z.C. (2009). Geometric characteristics of hydrogen bonds involving sulfur atoms in proteins. Proteins Struct. Funct. Bioinform..

[19] Nishio M. (2011). The CH/π hydrogen bond in chemistry. Conformation, supramolecules, optical resolution and interactions involving carbohydrates. Phys. Chem. Chem. Phys..

[20] Nanda V., Schmiedekamp A. (2008). Are aromatic carbon donor hydrogen bonds linear in proteins?. Proteins Struct. Funct. Bioinform..

[21] Brandl M., Weiss M.S., Jabs A., Suhnel J., Hilgenfeld R. (2001). C-H···π-interactions in proteins. J. Mol. Biol..

[22] Plevin M.J., Bryce D.L., Boisbouvier J. (2010). Direct detection of CH/π interactions in proteins. Nat. Chem..

[23] Kubickova A., Krizek T., Coufal P., Wernersson E., Heyda J., Jungwirth P. (2011). Guanidinium Cations Pair with Positively Charged Arginine Side Chains in Water. J. Phys. Chem. Lett..

[24] Morozov A.V., Kortemme T., Tsemekhman K., Baker D. (2004). Close agreement between the orientation dependence of hydrogen bonds observed in protein structures and quantum mechanical calculations. Proc. Natl. Acad. Sci. USA.

[25] Thomas A., Benhabiles N., Meurisse R., Ngwabije R., Brasseur R. (2001). Pex, analytical tools for PDB files. II. H-Pex: Noncanonical H-bonds in α-helices. Proteins Struct. Funct. Bioinform..

[26] Chen J.C.H., Hanson B.L., Fisher S.Z., Langan P., Kovalevsky A.Y. (2012). Direct observation of hydrogen atom dynamics and interactions by ultrahigh resolution neutron protein crystallography. Proc. Natl. Acad. Sci. USA.

[27] Cordier F., Grzesiek S. (1999). Direct observation of hydrogen bonds in proteins by interresidue ^3h^J_NC’_ scalar couplings. J. Am. Chem. Soc..

[28] O’Meara M.J., Leaver-Fay A., Tyka M.D., Stein A., Houlihan K., DiMaio F., Bradley P., Kortemme T., Baker D., Snoeyink J. (2015). Combined Covalent-Electrostatic Model of Hydrogen Bonding Improves Structure Prediction with Rosetta. J. Chem. Theory Comput..

[29] Forrest L.R., Honig B. (2005). An assessment of the accuracy of methods for predicting hydrogen positions in protein structures. Proteins Struct. Funct. Bioinform..

[30] Bas D.C., Rogers D.M., Jensen J.H. (2008). Very fast prediction and rationalization of pK_a_ values for protein-ligand complexes. Proteins Struct. Funct. Bioinform..

[31] Li H., Robertson A.D., Jensen J.H. (2005). Very fast empirical prediction and rationalization of protein pK_a_ values. Proteins Struct. Funct. Bioinform..

[32] Warshel A., Sharma P.K., Kato M., Xiang Y., Liu H.B., Olsson M.H.M. (2006). Electrostatic basis for enzyme catalysis. Chem. Rev..

[33] Watney J.B., Agarwal P.K., Hammes-Schiffer S. (2003). Effect of mutation on enzyme motion in dihydrofolate reductase. J. Am. Chem. Soc..

[34] Krivov G.G., Shapovalov M.V., Dunbrack R.L. (2009). Improved prediction of protein side-chain conformations with SCWRL4. Proteins Struct. Funct. Bioinform..

[35] Merski M., Fischer M., Balius T.E., Eidam O., Shoichet B.K. (2015). Homologous ligands accommodated by discrete conformations of a buried cavity. Proc. Natl. Acad. Sci. USA.

[36] Huynh M.T., Mora S.J., Villalba M., Tejeda-Ferrari M.E., Liddell P.A., Cherry B.R., Teillout A.L., Machan C.W., Kubiak C.P., Gust D. (2017). Concerted One-Electron Two-Proton Transfer Processes in Models Inspired by the Tyr-His Couple of Photosystem II. ACS Cent. Sci..

[37] Schutz C.N., Warshel A. (2004). The low barrier hydrogen bond (LBHB) proposal revisited: The case of the asp his pair in serine proteases. Proteins Struct. Funct. Bioinform..

[38] Shoichet B.K. (2004). Virtual screening of chemical libraries. Nature.

[39] Berman H.M., Westbrook J., Feng Z., Gilliland G., Bhat T.N., Weissig H., Shindyalov I.N., Bourne P.E. (2000). The Protein Data Bank. Nucleic Acids Res..

[40] Barlow D.J., Thornton J.M. (1983). Ion-Pairs in Proteins. J. Mol. Biol..

[41] Steiner T., Koellner G. (2001). Hydrogen bonds with π-acceptors in proteins: Frequencies and role in stabilizing local 3D structures. J. Mol. Biol..

[42] McRee D. (1999). Practical Protein Crystallography.

[43] Panigrahi S.K., Desiraju G.R. (2007). Strong and weak hydrogen bonds in the protein-ligand interface. Proteins Struct. Funct. Bioinform..

[44] Davis I.W., Leaver-Fay A., Chen V.B., Block J.N., Kapral G.J., Wang X., Murray L.W., Arendall W.B., Snoeyink J., Richardson J.S. (2007). MolProbity: All-atom contacts and structure validation for proteins and nucleic acids. Nucleic Acids Res..

[45] Guerois R., Nielsen J.E., Serrano L. (2002). Predicting changes in the stability of proteins and protein complexes: A study of more than 1000 mutations. J. Mol. Biol..

[46] Kortemme T., Morozov A.V., Baker D. (2003). An orientation-dependent hydrogen bonding potential improves prediction of specificity and structure for proteins and protein–protein complexes. J. Mol. Biol..

[47] Ibrahim B.S., Pattabhi V. (2004). Role of weak interactions in thermal stability of proteins. Biochem. Biophys. Res. Commun..

[48] Trachsel M.A., Ottigeri P., Frey H.M., Pfaffen C., Bihlmeier A., Klopper W., Leutwyler S. (2015). Modeling the Histidine–Phenylalanine Interaction: The NH···π Hydrogen Bond of Imidazole Benzene. J. Phys. Chem. B.

[49] Steiner T., Desiraju G.R. (1998). Distinction between the weak hydrogen bond and the van der Waals interaction. Chem. Commun..

[50] Karshikoff A., Jelesarov I. (2008). Salt bridges and conformational flexibility: Effect on protein stability. Biotechnol. Biotechnol. Equip..

[51] Wahl M.C., Sundaralingam M. (1997). C-H···O hydrogen bonding in biology. Trends Biochem. Sci..

[52] Chen V.B., Arendall W.B., Headd J.J., Keedy D.A., Immormino R.M., Kapral G.J., Murray L.W., Richardson J.S., Richardson D.C. (2010). MolProbity: All-atom structure validation for macromolecular crystallography. Acta Crystallogr. Sect. D Biol. Crystallogr..

[53] Gurusaran M., Sivaranjan P., Kumar K.S.D., Radha P., Tharshan K.P.S.T., Satheesh S.N., Jayanthan K., Ilaiyaraja R., Mohanapriya J., Michael D. (2016). Hydrogen Bonds Computing Server (HBCS): An online web server to compute hydrogen-bond interactions and their precision. J. Appl. Crystallogr..

[54] Laurence C., Berthelot M. (2000). Observations on the strength of hydrogen bonding. Perspect. Drug Discov. Des..

[55] Overington J., Johnson M.S., Sali A., Blundell T.L. (1990). Tertiary Structural Constraints on Protein Evolutionary Diversity: Templates, Key Residues and Structure Prediction. Proc. R. Soc. B: Biol. Sci..

[56] Li A.J., Nussinov R. (1998). A set of van der Waals and Coulombic radii of protein atoms for molecular and solvent-accessible surface calculation, packing evaluation, and docking. Proteins Struct. Funct. Bioinform..

[57] Jiang L., Lai L.H. (2002). CH···O hydrogen bonds at protein-protein interfaces. J. Biol. Chem..

[58] Escudero D., Frontera A., Quinonero D., Deya P.M. (2008). Interplay between edge-to-face aromatic and hydrogen-bonding interactions. J. Phys. Chem. A.

[59] Cohen M., Reichmann D., Neuvirth H., Schreiber G. (2008). Similar chemistry, but different bond preferences in inter versus intra-protein interactions. Proteins Struct. Funct. Bioinform..

[60] Newberry R.W., Raines R.T. (2016). A prevalent intraresidue hydrogen bond stabilizes proteins. Nat. Chem. Biol..

[61] Tsai J., Taylor R., Chothia C., Gerstein M. (1999). The packing density in proteins: Standard radii and volumes. J. Mol. Biol..

[62] Kroon J., Kanters J.A. (1974). Non-linearity of Hydrogen Bonds in Molecular Crystals. Nature.

