# Supplementary material for: A Geometric Definition of Short to Medium Range Hydrogen-Mediated Interactions in Proteins
Source: Molecules. 2020 Nov 15;25(22):5326. doi: 10.3390/molecules25225326 (PMC7696500; doi:10.3390/molecules25225326)
Supplement: Supplementary file 1 [file molecules-25-05326-s001.pdf]

Supplemental Material for “A Geometric Definition of Short to Medium Range Hydrogen Mediated Interactions in Proteins”

Matthew Merski [1,*], Jakub Skrzeczkowski [1], Jennifer K. Roth [2] and Maria W. Górna [1,*]

[1] Biological and Chemical Research Centre, Department of Chemistry, University of Warsaw, 02-089 Warsaw, Poland;

[2] Department of Psychology, Carlow University, Pittsburgh, PA, 15213 USA;

* Correspondence: merski@gmail.com (M.M.); mgorna@chem.uw.edu.pl (M.W.G.); Tel.: +48-22-552-6642 (M.M.); +48-225-526-685 (M.W.G.)

Table S1: Table of identified hydrogen mediated interactions identified in the PDB. Table lists the atom type, the average interaction distances and angles as well as the range of the quartiles (25-75%) as well as the total volume of the peak in the heat map. Interactions are divided up based on donors and acceptors in both the direct and indirect orientations. The qualifications column lists the those which are an accepted HMI (HMI), those in which the donor and acceptor atom are less than 2.5 Å distant (short), those which the total volume is does not surpass the threshold (vol), and those which the alternative orientation does not have this interaction (*e.g.* direct or indirect)

**DONORS (Direct)**									
**Atom**	**#**	**[Distance]**	**Dist 25**	**Dist 75**	**[Angle]**	**Ang 25**	**Ang 75**	**Vol**	**qualifications**
CA	1	2.4	2.4	2.4	97	93	99	744.4	short
	2	4.3	4.2	4.3	82	81	83	21.6	vol
	3	4.5	4.4	4.6	111	109	113	96.3	vol
	4	5.1	5.0	5.1	111	110	112	34.5	vol
	5	3.3	3.3	3.4	152	150	154	212.4	vol
CB	1	2.4	2.4	2.4	91	89	92	402.9	vol
	2	3.2	3.1	3.4	98	91	104	412.3	vol
	3	3.4	3.4	3.5	146	145	147	54.5	vol
CD	1	4.9	4.9	4.9	91	90	92	18.6	vol
	2	2.9	2.9	3.0	126	126	127	19.9	vol
	3	3.3	3.3	3.4	162	159	164	68.82	vol
CE	1	5.3	5.3	5.4	97	96	98	30.2	vol
CE1	1	3.4	3.3	3.4	140	138	142	79.9	vol
	2	3.4	3.4	3.5	179	178	180	20	vol
CE2	1	3.4	3.3	3.5	139	137	141	143.1	vol
	2	3.7	3.6	3.7	179	178	179	22.5	vol
CE3	1	5.9	5.8	5.9	129	128	130	16.6	vol
	2	4.7	4.6	4.8	148	146	149	45.1	vol
	3	3.5	3.4	3.6	179	177	180	103.4	vol
	4	3.6	3.6	3.7	169	167	171	125.8	vol
	5	5.0	4.9	5.1	179	178	180	56.8	vol
CG	1	2.4	2.4	2.4	90	89	91	36.7	vol
	2	3.0	3.0	3.1	108	103	118	324.5	vol
	3	3.4	3.4	3.6	129	127	132	112.4	vol
CG1	1	3.2	3.1	3.2	82	81	83	79	vol
	2	4.7	4.6	4.7	83	82	85	41.9	vol
	3	3.0	2.9	3.0	104	101	108	229.8	vol
CG2	1	3.2	3.1	3.2	82	81	82	81.1	vol
	2	3.3	3.3	3.4	89	88	90	48.6	vol
	3	3.9	3.8	4.0	123	122	124	34.4	vol
CH2	1	3.6	3.5	3.6	167	164	168	37.13	vol
	2	3.7	3.6	3.7	177	175	179	98.49	vol
CZ	1	3.6	3.5	3.6	171	169	174	63.24	vol
CZ2	1	3.7	3.6	4.0	131	128	135	358	vol
	2	3.5	3.5	3.6	166	161	170	137.7	vol
	3	3.4	3.4	3.5	180	179	180	26.3	vol
CZ3	1	3.7	3.6	3.7	137	136	139	22.1	vol
	2	3.6	3.5	3.7	175	170	177	168.3	vol
N	1	3.2	3.2	3.3	125	124	127	46.5	vol

	2	2.7	2.7	2.7	131	130	132	35.6	vol
	3	5.0	4.9	5.0	148	147	149	23.3	vol
	4	2.9	2.8	3.1	171	166	176	2126	HMI
ND1	1	2.7	2.7	2.8	173	168	177	1511	HMI
ND2	1	2.9	2.8	2.9	120	114	126	719.4	HMI
NE	1	2.8	2.7	2.9	171	165	176	1678	HMI
NE1	2	2.8	2.8	2.9	172	166	176	1659	HMI
NE2	1	2.9	2.8	2.9	121	118	126	177.1	vol
	2	2.7	2.7	2.8	173	169	177	766	HMI
NH1	1	2.9	2.8	2.9	97	96	99	56.0	vol
	2	2.8	2.8	2.9	132	127	136	575.6	HMI
NH2	1	2.9	2.8	3.0	105	92	119	826.8	HMI
NZ	2	2.8	2.7	2.8	107	99	114	777	HMI
OD1	1	2.9	2.8	3.0	85	83	88	129.3	vol
	2	2.8	2.8	2.9	136	124	151	839.4	HMI
OD2	1	3.4	3.3	3.5	83	81	84	66	vol
	2	2.8	2.7	2.8	125	118	133	779.9	HMI
OE1	1	5.5	5.4	5.6	88	87	89	22.3	vol
	2	2.8	2.7	2.9	131	122	139	376.7	vol
OE2	1	3.4	3.4	3.5	82	81	83	24	vol
	2	2.8	2.7	2.8	123	117	130	589.5	HMI
OG	1	3.1	3.0	3.2	84	82	86	256	vol
	2	2.7	2.6	2.8	113	105	120	529	HMI
	3	3.0	3.0	3.0	142	140	143	27.8	vol
OG1	1	3.1	3.0	3.2	83	82	85	128.1	vol
	2	2.7	2.6	2.8	112	106	118	503.8	HMI
	3	3.0	2.9	3.0	141	139	143	78	vol
OH	1	2.6	2.6	2.7	115	112	119	609.9	HMI
SG	2	2.0	2.0	2.0	105	103	106	637.2	short

**DONORS (INDIRECT)**									
**Atom**	**#**	**[Distance]**	**Dist 25**	**Dist 75**	**[Angle]**	**Ang 25**	**Ang 75**	**Vol**	**qualifications**
CA	1	2.4	2.4	2.4	100	100	101	710	short
	2	3.4	3.3	3.5	85	85	86	48	vol
	3	4.7	4.7	4.8	82	81	83	24.2	vol
	4	3.3	3.3	3.3	152	151	153	15.3	vol
	5	4.5	4.4	4.6	154	152	157	286	vol
	6	5.1	5.0	5.2	162	159	164	200	vol
CB	1	2.4	2.4	2.4	91	89	92	406	vol
	2	3.2	3.1	3.3	105	100	108	356	vol
	3	3.5	3.5	3.6	117	115	120	100	vol
	4	3.4	3.4	3.5	148	145	151	171	vol
CD	1	4.9	4.9	4.9	92	91	94	40	vol
	2	3.5	3.4	3.7	116	113	118	130.3	vol
CD1	1	3.7	3.6	3.7	82	81	83	32.1	vol
CD2	1	3.7	3.6	3.8	85	83	87	112	vol
	2	4.7	4.6	4.7	82	81	83	20.9	vol
CE3	1	3.5	3.4	3.6	84	82	86	114.7	vol
	2	4.4	4.4	4.5	87	86	88	18.8	vol
CG	1	2.4	2.4	2.4	92	91	93	38.3	vol
	2	3.0	3.0	3.1	102	98	104	321.9	vol
	3	3.7	3.7	3.8	89	88	91	66.1	vol

	4	4.6	4.6	4.6	110	109	111	19.7	vol
	5	3.3	3.2	3.4	141	138	144	143.1	vol
CG1	1	3.0	2.9	3.0	104	102	106	211.2	vol
	2	4.7	4.6	4.7	106	105	108	39.0	vol
	3	3.3	3.3	3.3	140	139	142	19.7	vol
CG2	1	3.3	3.3	3.4	140	139	142	34.9	vol
	2	3.7	3.6	3.7	116	113	119	78.0	vol
CZ3	1	3.7	3.6	3.7	93	91	94	36.6	vol
	2	3.7	3.7	3.8	103	100	105	76.4	vol
N	1	3.2	3.2	3.3	105	102	107	209.7	vol
	2	2.7	2.7	2.8	130	128	132	105.9	vol
	3	5.0	4.9	5.0	135	134	137	37.7	vol
	4	2.9	2.8	2.9	159	154	166	1196.8	HMI
ND1	1	2.9	2.8	2.9	142	141	143	22.2	vol
	2	2.9	2.8	3.0	170	166	176	352.8	HMI
ND2	1	2.9	2.8	2.9	142	131	156	965.9	HMI
NE	1	2.8	2.7	2.8	135	120	148	349.3	HMI
NE1	1	2.8	2.8	2.9	135	125	142	367.6	HMI
	2	2.8	2.8	2.8	160	158	161	26	vol
	3	2.8	2.8	2.8	170	167	172	33.7	vol
NE2	1	2.8	2.8	2.9	141	132	154	601.2	HMI
NH1	1	2.8	2.8	2.9	140	129	152	953	HMI
NH2	1	3.5	3.4	3.5	82	81	83	28	vol
	2	2.8	2.8	2.9	137	123	150	985.8	HMI
NZ	1	2.8	2.7	2.8	148	134	163	1232	HMI
OD1	1	2.8	2.7	2.9	128	125	131	58.1	vol
	2	2.8	2.8	2.9	171	164	176	1414	HMI
OD2	1	2.6	2.6	2.6	115	113	117	22.9	vol
	2	2.8	2.7	2.9	173	168	177	1562	HMI
OE1	1	2.8	2.7	2.9	173	167	177	1305	rev
OE2	2	2.8	2.7	2.9	174	168	177	1334	HMI
OG	1	3.2	3.1	3.2	106	104	107	28.1	HMI
	2	2.8	2.7	3.0	164	150	172	1239.4	HMI
OG1	1	2.8	2.7	2.9	160	144	169	1234.3	HMI
OH	1	2.6	2.5	2.6	131	122	139	502.2	HMI
	2	2.8	2.7	2.9	174	169	177	576.8	HMI
SG	1	2.0	2.0	2.0	105	103	106	642	short
	2	3.4	3.3	3.4	138	135	140	65.4	vol
	3	3.4	3.3	3.4	164	163	165	38.2	vol
	4	3.5	3.4	3.6	178	176	179	77.1	vol

ACCEPTORS (DIRECT)									
**Atom**	**#**	**[Distance]**	**Dist 25**	**Dist 75**	**[Angle]**	**Ang 25**	**Ang 75**	**Vol**	**qualifications**
CD2	1	3.5	3.3	3.5	88	85	92	302.0	rev
	2	3.0	3.0	3.1	109	107	111	82.5	vol
CE1	1	3.7	3.6	3.8	93	87	98	438.7	HMI
N	1	2.4	2.4	2.4	91	89	92	225.7	short
	2	2.4	2.4	2.4	100	99	101	430.5	short
	3	3.1	3.1	3.2	104	101	107	268.3	rev
	4	2.7	2.7	2.7	131	130	132	40.5	vol
	5	4.5	4.4	4.5	153	151	155	159.6	rev
	6	5.1	5.0	5.2	161	159	164	79.2	vol
	7	2.8	2.8	2.9	176	174	179	94.4	vol
NE	1	2.4	2.4	2.4	92	91	93	190.8	short
	2	3.0	3.0	3.0	91	89	93	30.4	vol
	3	3.0	3.0	3.0	105	104	107	27.6	vol
	4	4.9	4.9	5.0	91	91	92	17	vol
	5	2.8	2.7	2.9	172	168	176	867.4	rev
ND1	1	3.2	3.1	3.3	95	91	99	304.8	HMI
	2	2.7	2.7	2.8	173	169	177	738.6	rev
NE2	1	2.7	2.6	2.8	174	170	177	329.2	rev
NH1	1	3.5	3.5	3.6	82	81	83	18.8	vol
	2	2.8	2.8	2.9	133	132	134	42.5	vol
NH2	1	3.6	3.5	3.7	85	83	87	202.1	rev
	2	2.8	2.8	2.9	117	115	119	94.9	vol
NZ	1	2.7	2.7	2.8	107	102	111	173	rev
O	1	3.3	3.2	3.3	105	103	107	90.6	vol
	2	2.9	2.8	3.2	156	150	162	1073	HMI
OD1	1	2.9	2.8	2.9	85	82	88	55.1	vol
	2	3.1	3.1	3.2	111	110	112	47.7	vol
	3	2.8	2.8	2.9	137	131	144	181.9	HMI
	4	2.8	2.8	2.8	178	177	179	16.4	vol
OD2	1	2.8	2.7	2.8	124	119	132	323.9	HMI
OE2	1	2.8	2.7	2.8	123	119	127	160	HMI
OG	1	3.1	3.0	3.2	84	82	85	137.8	vol
	2	4.4	4.3	4.4	91	89	92	26	vol
	3	3.4	3.4	3.5	176	173	177	44.4	vol
OG1	1	3.1	3.1	3.2	83	82	84	56.4	vol
	2	4.6	4.5	4.7	86	83	88	71.4	vol
OH	1	3.6	3.6	3.6	86	83	88	47.8	vol
	2	2.6	2.5	2.6	115	113	118	153.5	HMI
SD	1	2.7	2.7	2.7	82	81	82	51.2	vol
	2	3.8	3.8	3.9	140	132	147	439	HMI
	3	3.8	3.8	3.9	166	165	167	24.8	vol
	4	3.8	3.8	3.8	172	172	174	19.2	vol
SG	1	2.0	2.0	2.0	105	103	106	322.9	short
	2	3.7	3.7	3.8	151	150	151	15.2	vol
	3	3.7	3.7	3.7	173	171	174	20.2	vol
X1	1	3.2	3.2	3.3	85	84	86	151	rev
	2	2.1	2.0	2.2	156	148	167	458	short
X2	1	3.5	3.4	3.5	86	85	86	114	vol
	2	2.2	2.0	2.3	155	146	167	756	rev
X3	1	3.5	3.4	3.5	85	84	86	102	vol
	2	2.1	2.0	2.3	158	149	169	648	short

X4	1	3.3	3.2	3.3	85	84	86	109	vol
	2	2.2	2.1	2.4	149	139	162	817	short
X5	1	4.4	4.3	4.5	86	86	87	31.6	vol
	2	2.2	2.1	2.4	154	142	167	1080.3	short

**ACCEPTORS (INDIRECT)**									
**Atom**	**#**	**[Distance]**	**Dist 25**	**Dist 75**	**[Angle]**	**Ang 25**	**Ang 75**	**Vol**	**qualifications**
CD2	1	3.0	3.0	3.0	112	109	114	52.2	vol
	2	3.4	3.3	3.5	109	107	112	127.6	vol
	3	3.4	3.4	3.5	84	82	86	57.4	vol
	4	3.5	3.5	3.6	177	176	178	19.4	vol
CE1	1	3.7	3.6	3.8	157	150	164	590.8	HMI
N	1	2.4	2.4	2.4	94	90	98	706.8	short
	2	3.1	3.0	3.2	103	100	106	19.8	vol
	3	3.2	3.1	3.3	82	81	83	198.9	vol
	4	2.7	2.7	2.7	131	130	132	44	vol
NE	1	2.4	2.4	2.4	90	88	91	229.6	short
	2	2.9	2.9	3.0	109	105	113	296.9	rev
	3	4.2	4.1	4.2	177	176	179	71.1	vol
ND1	1	2.5	2.5	2.5	85	83	86	107	vol
	2	3.3	3.2	3.4	85	83	87	50.4	vol
	3	3.2	3.1	3.3	112	109	115	235.6	HMI
	4	2.9	2.9	3.0	172	167	177	168.6	vol
ND2	1	3.6	3.6	3.6	162	160	163	28	vol
	2	3.6	3.6	3.6	168	167	168	15.6	vol
	3	3.7	3.6	3.8	175	173	176	45.3	vol
NE2	1	3.7	3.7	3.8	177	176	179	75.7	vol
NH2	1	2.8	2.8	2.9	117	115	120	61.8	vol
	2	3.6	3.6	3.7	152	151	153	21.7	vol
	3	3.7	3.7	3.7	159	158	160	15.6	vol
NZ	1	2.7	2.7	2.8	129	124	134	157.8	vol
O	1	3.3	3.2	3.3	125	124	127	36.5	vol
	2	2.9	2.8	3.0	170	164	176	1478	HMI
OD1	1	2.8	2.8	2.9	171	164	176	957.9	HMI
OD2	1	2.8	2.7	2.9	173	168	177	1001	HMI
OE1	1	2.8	2.7	2.9	174	168	177	767.4	rev
	2	3.8	3.8	3.9	178	176	179	41.3	vol
OE2	1	2.8	2.7	2.9	174	169	178	790.9	HMI
OG	1	2.9	2.8	3.0	168	164	175	574.4	rev
OG1	1	3.0	2.9	3.1	168	164	174	414.5	rev
OH	1	3.5	3.4	3.6	152	148	157	153.8	vol
	2	2.9	2.8	3.0	176	173	178	248.2	HMI
SD	1	2.7	2.7	2.7	95	94	96	36.3	vol
	2	3.8	3.7	4.0	163	152	174	855.8	HMI
SG	1	2.0	2.0	2.0	105	103	106	317	short
	2	3.8	3.7	3.9	158	150	171	475.2	rev
X1	1	3.2	3.2	3.2	113	108	119	75.3	vol
X3	1	3.5	3.5	3.5	112	109	115	34.3	vol

Table S2A: Table of amino acids and atom types present in the PDB (Donors). List of atoms analyzed as possible donor atoms.

	A	*C*	*D*	*E*	*F*	*G*	*H*	*I*	*K*	*L*	*M*	*N*	*P*	*Q*	*R*	*S*	*T*	*V*	*W*	*Y*
CA	***	***	***	***	***	***	***	***	***	***	***	***	***	***	***	***	***	***	***	***
CB	***	***	***	***	***		***	***	***	***	***	***	***	***	***	***	***	***	***	***
CD									***				***		***					
CD1					***			***		***									***	***
CD2					***		***			***										***
CE									***		***									
CE1					***		***													***
CE2					***															***
CE3																			***	
CG				***					***	***	***		***	***	***					
CG1								***										***		
CG2								***									***	***		
CH2																			***	
CZ					***															
CZ2																			***	
CZ3																			***	
N(D)	***	***	***	***	***	***	***	***	***	***	***	***	***	***	***	***	***	***	***	***
ND1							***													
ND2												***								
NE															***					
NE1																			***	
NE2							***							***						
NH1															***					
NH2															***					
NZ									***											
OD1			***									***								
OD2			***																	
OE1				***										***						
OE2				***																
OG																***				
OG1																	***			
OH																				***
SG		***																		

Table S2B: Table of amino acids and atom types present in the PDB (Acceptors). List of atoms analyzed as possible acceptor atoms. Atom types X1-X5 are virtual atoms used to represent the aromatic π electron clouds in histidine (X1), tyrosine (X2), phenylalanine (X3), and the tryptophan 5-membered (X4) and 6-membered (X5) rings respectively.

	A	*C*	*D*	*E*	*F*	*G*	*H*	*I*	*K*	*L*	*M*	*N*	*P*	*Q*	*R*	*S*	*T*	*V*	*W*	*Y*
**CD2**							***													
**CE1**							***													
**N(A)**	***	***	***	***	***	***	***	***	***	***	***	***	***	***	***	***	***	***	***	***
**NE**															***					
**ND1**							***													
**ND2**												***								
**NE2**							***							***						
**NH1**															***					
**NH2**															***					
**NZ**									***											
**O**	***	***	***	***	***	***	***	***	***	***	***	***	***	***	***	***	***	***	***	***
**OD1**			***									***								
**OD2**			***																	
**OE1**				***										***						
**OE2**				***																
**OG**																***				
**OG1**																	***			
**OH**																				***
**SD**											***									
**SG**		***																		
**X1**							***													
**X2**																				***
**X3**					***															
**X4**																			***	
**X5**																			***	

Table S3: Table identifying the partner atom for each analyzed atom (by type). This table identifies the partner atom for each analyzed atom (by type) in each amino acid A) in the main chain and B) side chain atoms. Blue indicates donors, yellow acceptors, while green indicates those that were analyzed as both donors and acceptors. In most cases, a single partner atom is indicated. In cases where multiple atoms are indicated, a virtual atom at a point equidistant from the listed atoms was used as a virtual partner atom. For the aromatic π clouds, the virtual point was placed at the center of the ring, while the acceptor atom was placed at points 1 Å away orthogonal to the plane of the ring.

A)

	N	CA	C	O	CB
Alanine	CA, [C(-1)]	N, C, CB	XXX	C	CA
Cysteine	CA, [C(-1)]	N, C, CB	XXX	C	CA, SG
Aspatate	CA, [C(-1)]	N, C, CB	XXX	C	CA, CG
Glutamate	CA, [C(-1)]	N, C, CB	XXX	C	CA, CG
Phenylalanine	CA, [C(-1)]	N, C, CB	XXX	C	CA, CG
Glycine	CA, [C(-1)]	N, C	XXX	C	
Histidine	CA, [C(-1)]	N, C, CB	XXX	C	CA, CG
Isoleucine	CA, [C(-1)]	N, C, CB	XXX	C	CA, CG1, CG2
Lysine	CA, [C(-1)]	N, C, CB	XXX	C	CA, CG
Leucine	CA, [C(-1)]	N, C, CB	XXX	C	CA, CG
Methionine	CA, [C(-1)]	N, C, CB	XXX	C	CA, CG
Asparagine	CA, [C(-1)]	N, C, CB	XXX	C	CA, CG
Proline	XXX	N, C, CB	XXX	C	CA, CG
Glutamine	CA, [C(-1)]	N, C, CB	XXX	C	CA, CG
Arginine	CA, [C(-1)]	N, C, CB	XXX	C	CA, CG
Serine	CA, [C(-1)]	N, C, CB	XXX	C	CA, OG
Threonine	CA, [C(-1)]	N, C, CB	XXX	C	CA, OG1, CG2
Valine	CA, [C(-1)]	N, C, CB	XXX	C	CA, CG1,CG2
Tryptophan	CA, [C(-1)]	N, C, CB	XXX	C	CA, CG
Tyrosine	CA, [C(-1)]	N, C, CB	XXX	C	CA, CG

B)

Alanine											
	**SG**										
Cysteine	CB										
	**CG**	**OD1**	**OD2**								
Aspatate	XXX	CG	CG								
	**CG**	**CD**	**OE1**	**OE2**							
Glutamate	CB, CD	XXX	CD	CD							
	**CG**	**CD1**	**CD2**	**CE1**	**CE2**	**CZ**	X				
Phenylalanine	XXX	CG, CD1,CD2,CE1,CE2,CZ	CG, CD1,CD2,CE1,CE2,CZ	CG, CD1,CD2,CE1,CE2,CZ	CG, CD1,CD2,CE1,CE2,CZ	CG, CD1,CD2,CE1,CE2,CZ	CG, CD1,CD2,CE1,CE2,CZ				
Glycine											
	**CG**	**ND1**	**CD2**	**CE1**	**NE2**	X					
Histidine	XXX	CG, CD2, NE2, CE1, ND1	CG, CD2, NE2, CE1, ND1	CG, CD2, NE2, CE1, ND1	CG, CD2, NE2, CE1, ND1	CG, CD2, NE2, CE1, ND1					
	**CG1**	**CG2**	**CD1**								
Isoleucine	CB, CD1	CB	CG1								
	**CG**	**CD**	**CE**	**NZ**							
Lysine	CB, CD	CG, CE	CD, NZ	CE							
	**CG**	**CD1**	**CD2**								
Leucine	CB, CD1, CD2	CG	CG								
	**CG**	**SD**	**CE**								
Methionine	CB, SD	CG, CE	SD								
	**CG**	**OD1**	**ND2**								
Asparagine	XXX	CG	CG								
	**CG**	**CD**									
Proline	CB, CD	N, CG									
	**CG**	**CD**	**OE1**	**NE2**							
Glutamine	CB, CD	XXX	CD	CD							
	**CG**	**CD**	**NE**	**CZ**	**NH1**	**NH2**					
Arginine	CB, CD	CG, NE	CZ, CD	XXX	CZ	CZ					
	**OG**										
Serine	CB										
	**OG1**	**CG2**									
Threonine	CB	CB									
	**CG1**	**CG2**									
Valine	CB	CB									
	**CG**	**CD1**	**CD2**	**NE1**	**CE2**	**CE3**	**CZ2**	**CZ3**	**CH2**	**X5**	**X6**
Tryptophan	XXX	CG, CD2, CE2, NE1, CD1	XXX	CG, CD2, CE2, NE1, CD1	XXX	CE3,CZ3,CH2,CZ2,CE2,CD2	CE3,CZ3,CH2,CZ2,CE2,CD2	CE3,CZ3,CH2,CZ2,CE2,CD2	CE3,CZ3,CH2,CZ2,CE2,CD2	CG, CD2, CE2, NE1, CD1	CE3,CZ3,CH2,CZ2,CE2,CD2
	**CG**	**CD1**	**CD2**	**CE1**	**CE2**	**CZ**	**OH**	X			
Tyrosine	XXX	CD1,CE1,CZ,CE2,CD2,CG	CD1,CE1,CZ,CE2,CD2,CG	CD1,CE1,CZ,CE2,CD2,CG	CD1,CE1,CZ,CE2,CD2,CG	XXX	CZ	CD1,CE1,CZ,CE2,CD2,CG			

Figure S1: Heat maps for each of the HMI. Heat maps are given for each of the HMI in SI Table 1 for the direct and indirect angle formulations, by PDB atom type. Note that some atom types such as N and OE2 are can be both acceptors and donors and were analyzed as both. Heat maps show the examined space in bins of 0.1Å distance and 1˚ angle increments. In all cases, the heat maps were normalized compared to the average value of all the bins. For all atom types, the direct arrangement is shown on the left and the indirect arrangement is shown on the right. Figures were generated in R. The color of the individual bins is incremented at normalized threshold values of 0.05, 0.1, 0.25, 0.5, 1, 2, 3, 4, 5, 10, 20 & 50, starting with dark blue at values of zero and rising to red for the highest values as below:

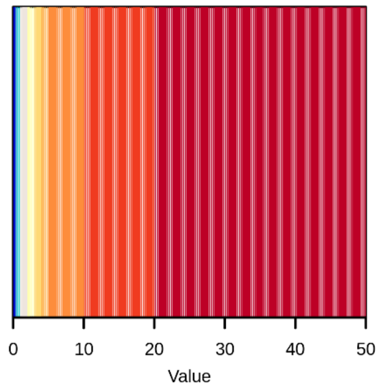

A) CA, donor

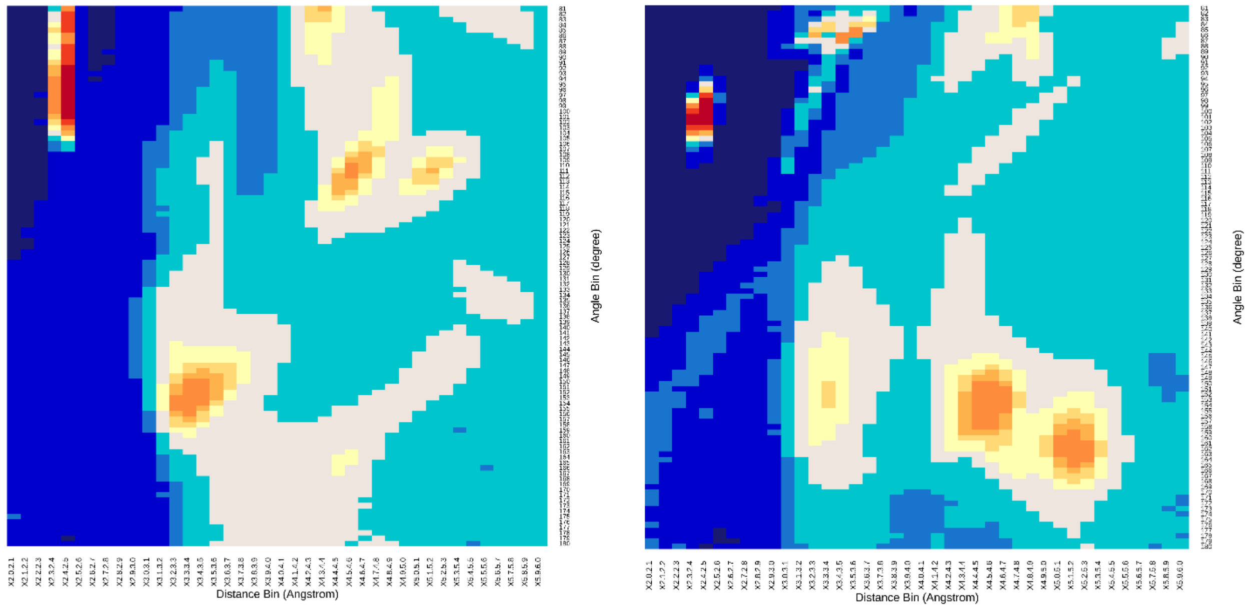

B) CB, donor

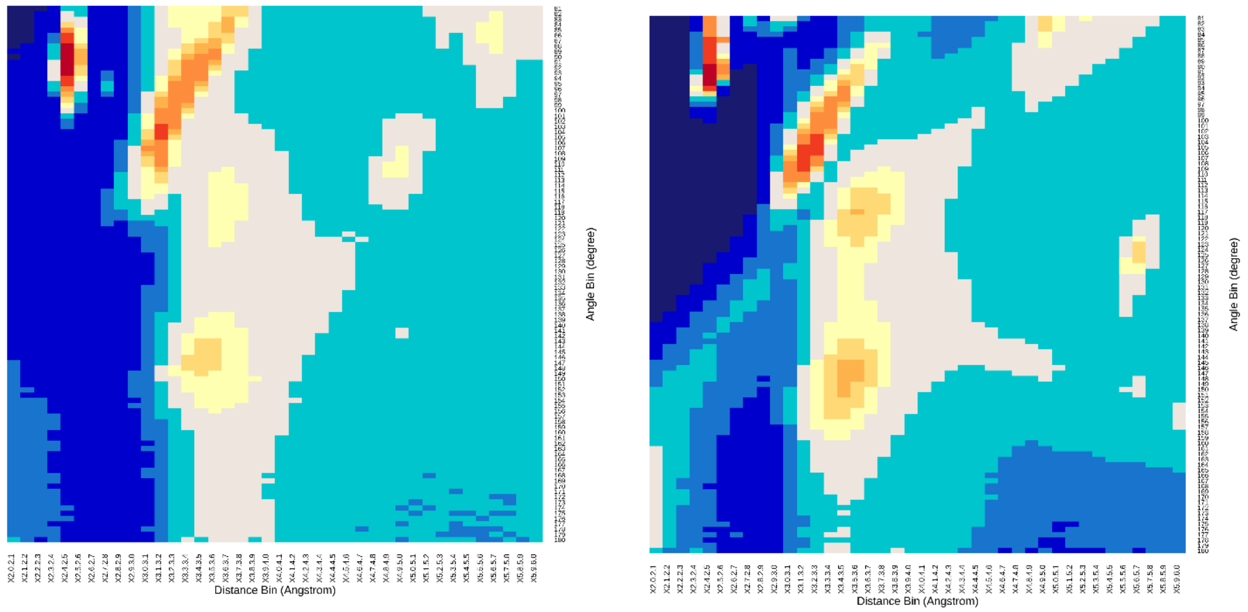

C) CD, donor

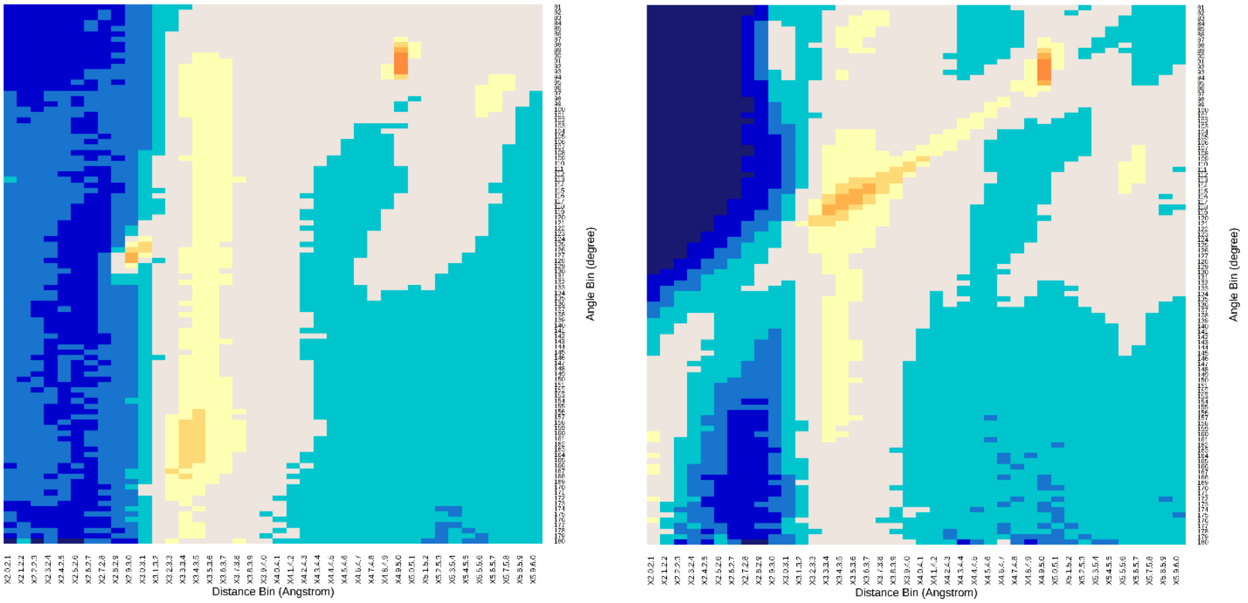

D) CE, donor

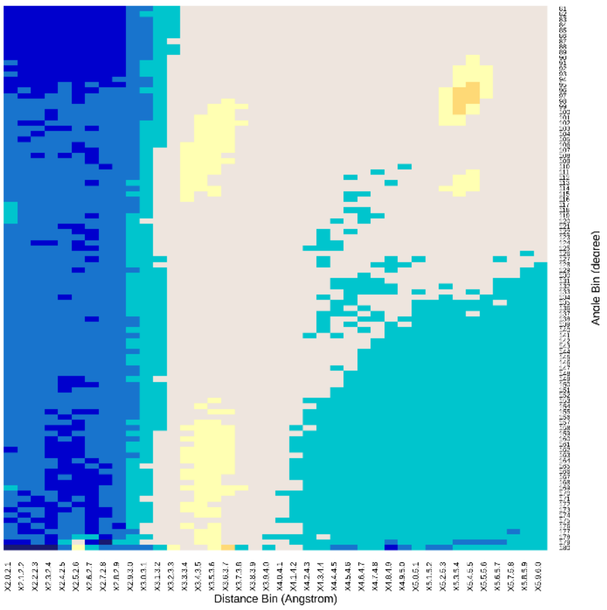

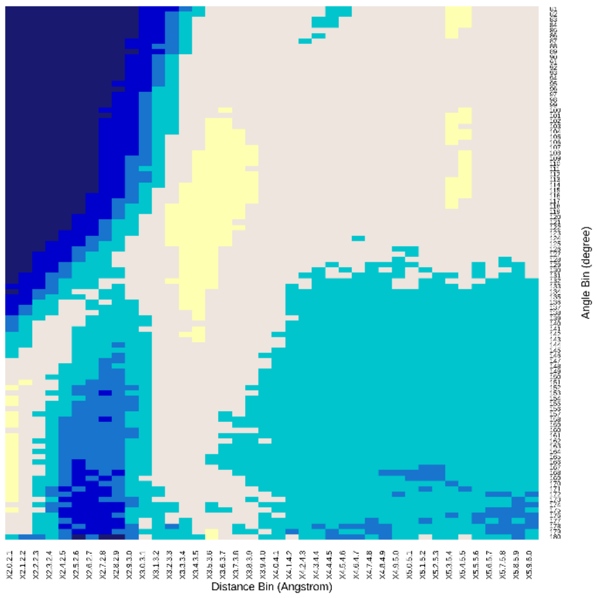

E) CE1, donor

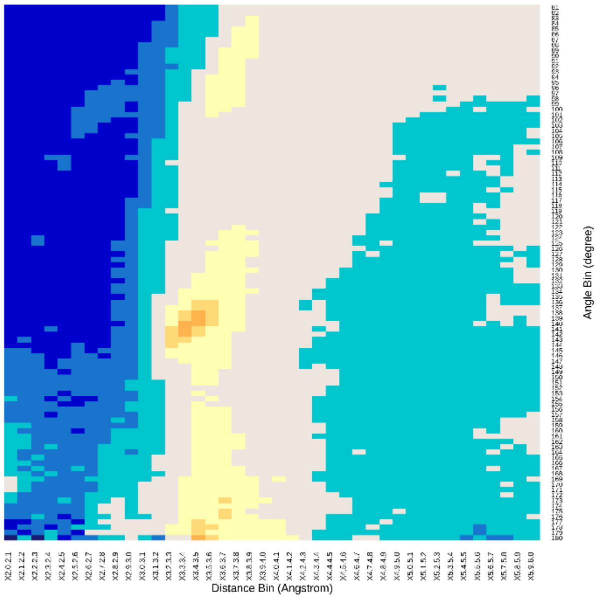

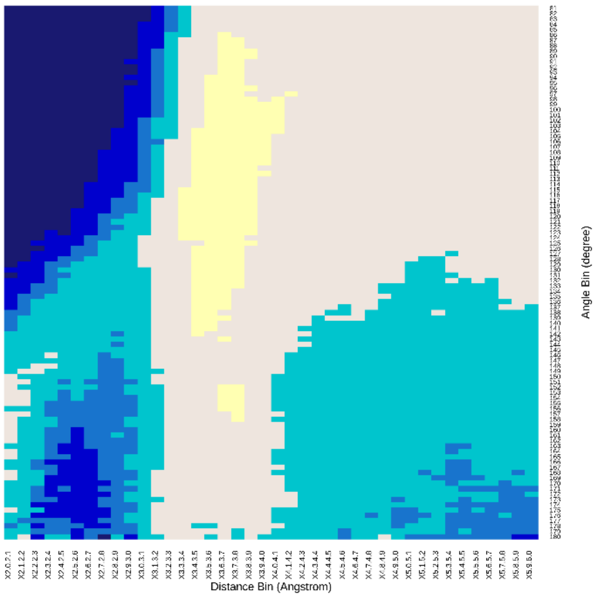

F) CE2, donor

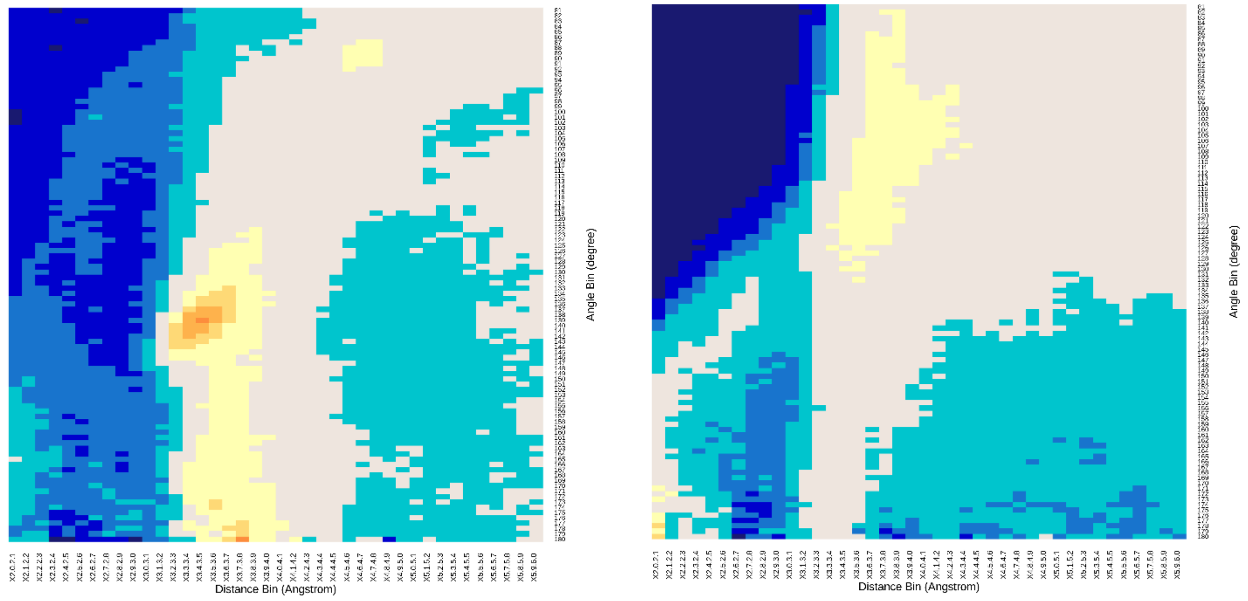

G) CE3, donor

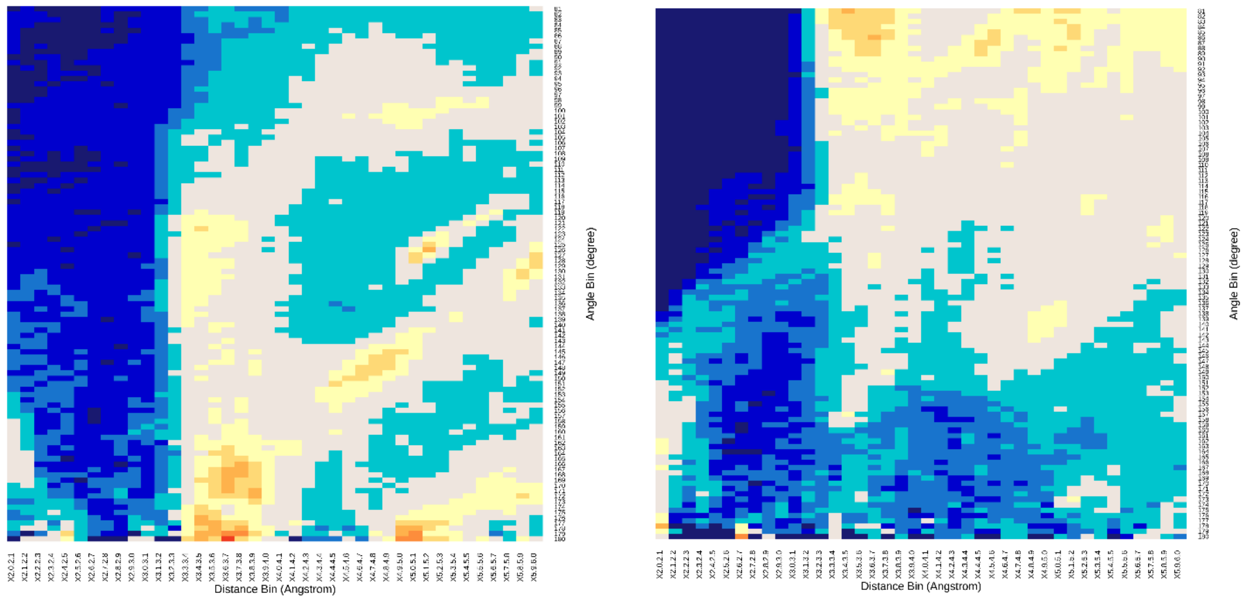

H) CG, donor

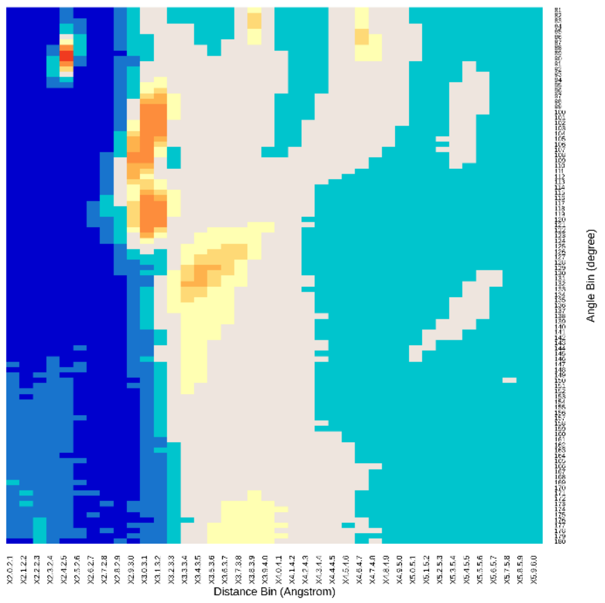

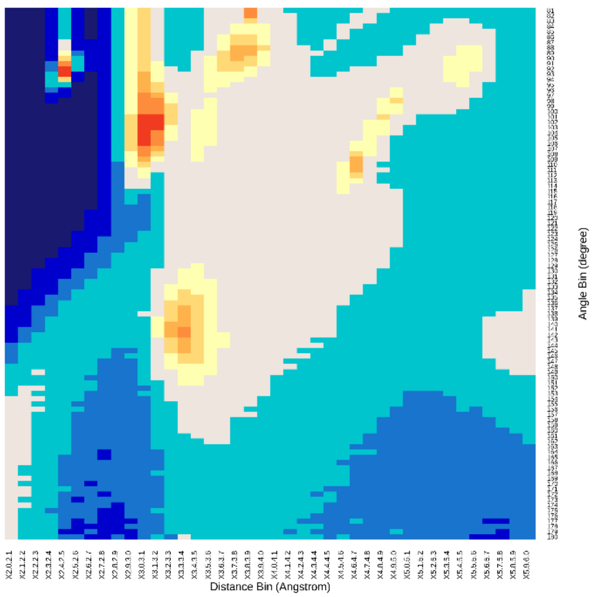

I) CG1, donor

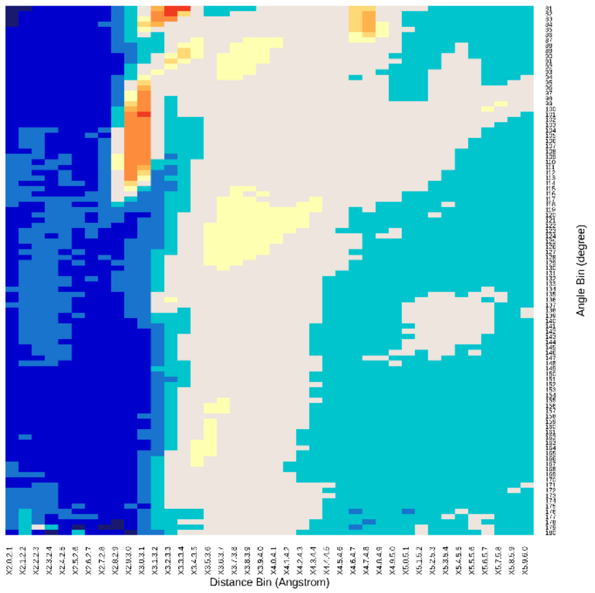

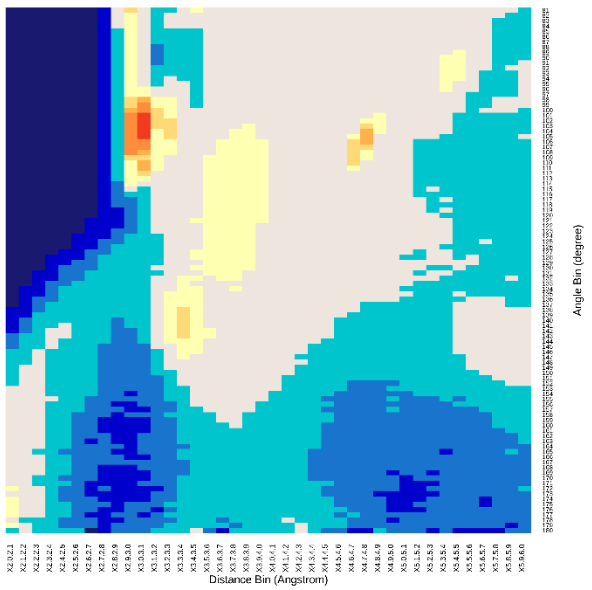

J) CG2, donor

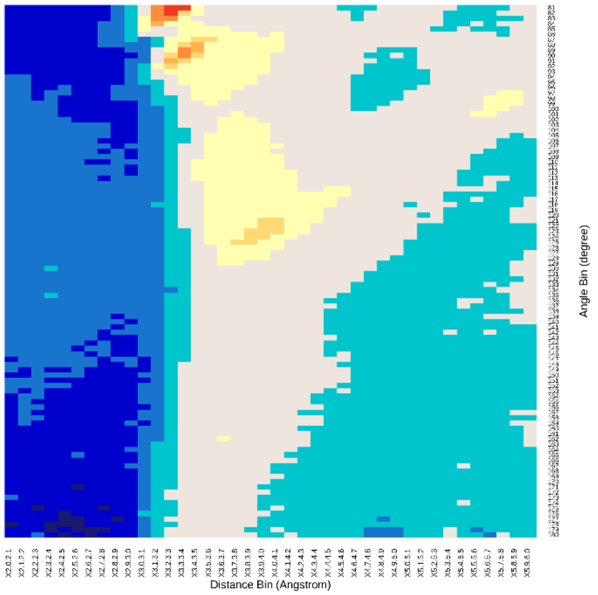

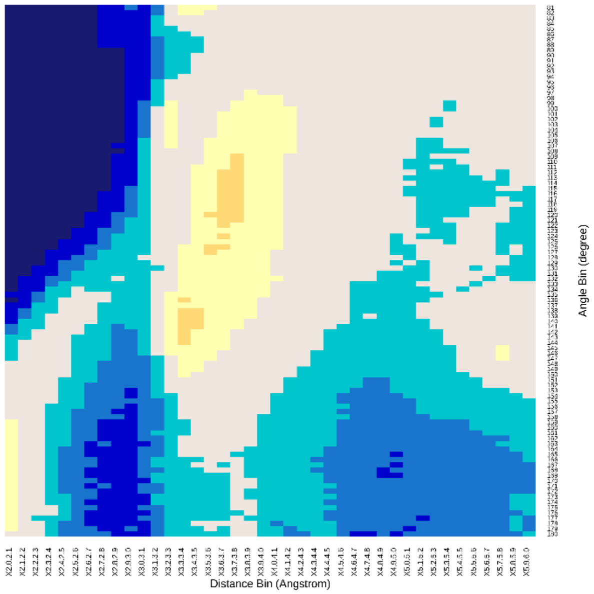

K) CH2, donor

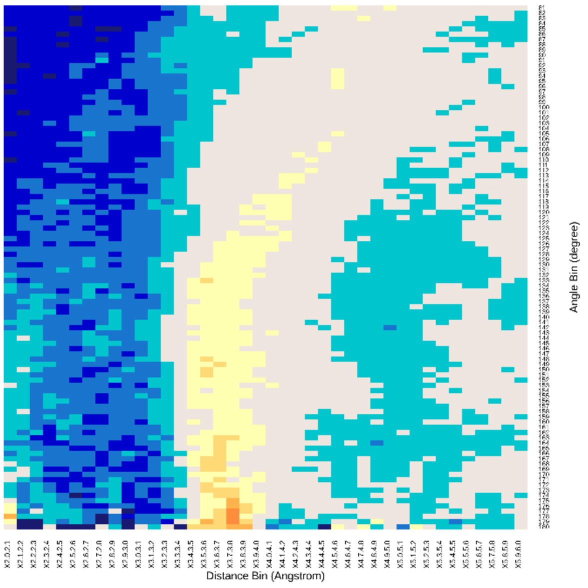

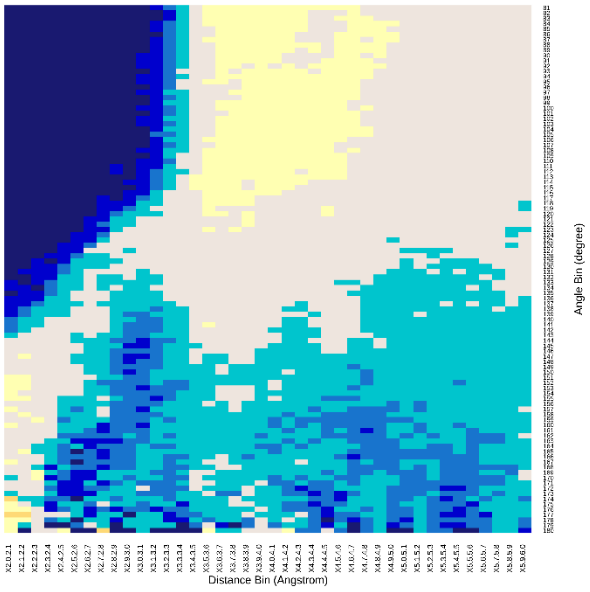

L) CZ, donor

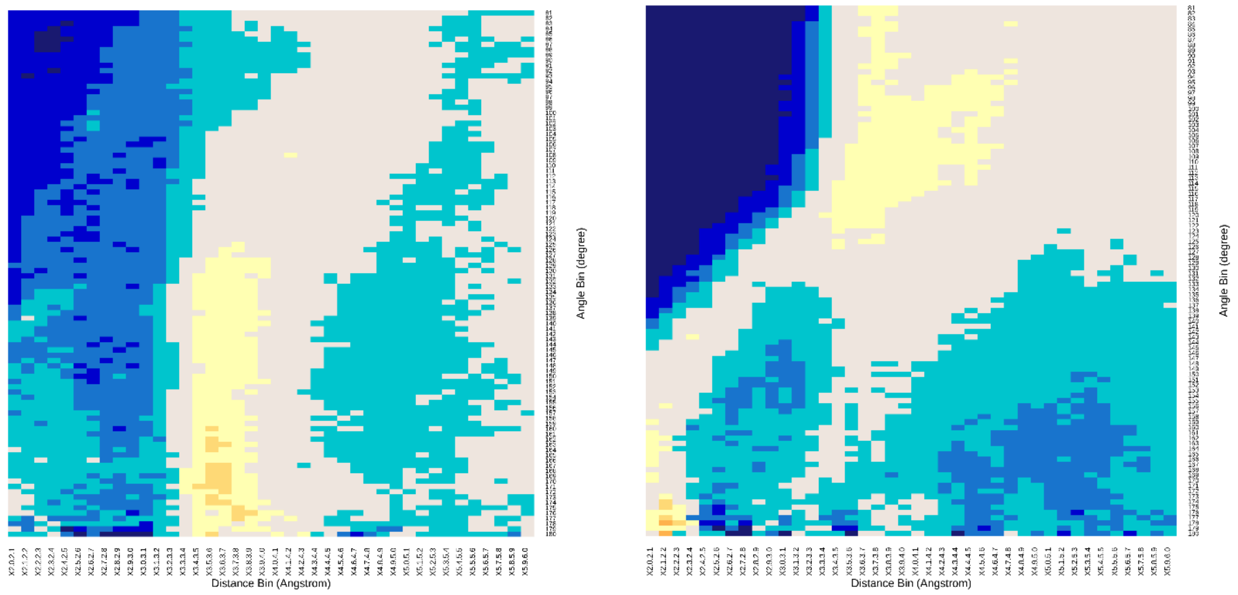

M) CZ2, donor

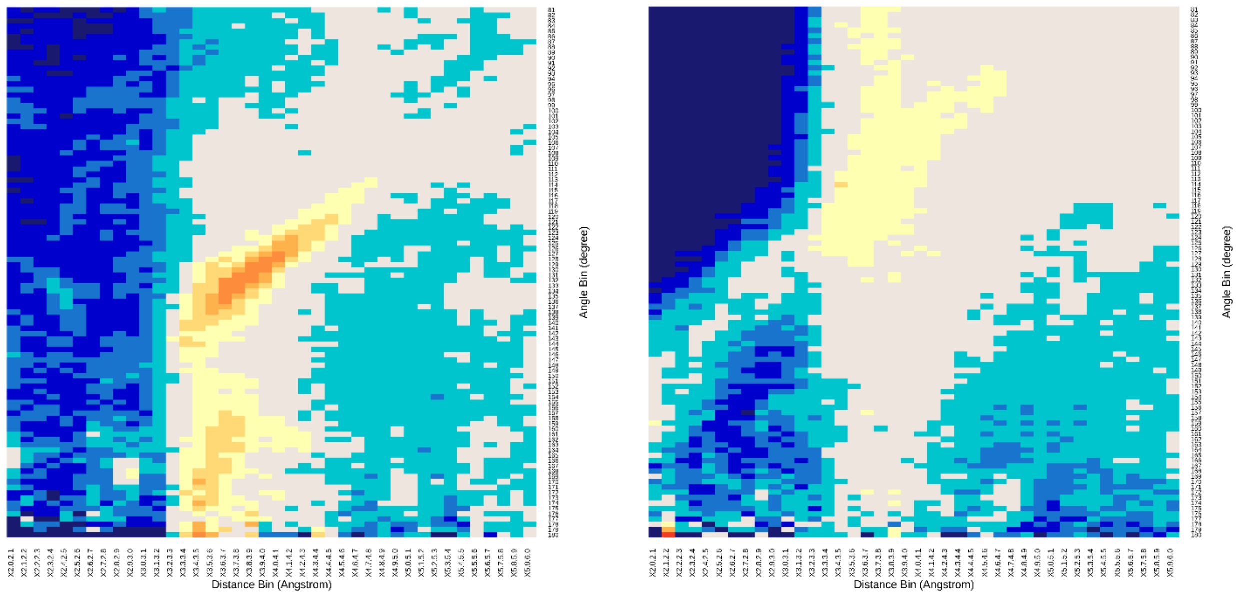

N) CZ3, donor

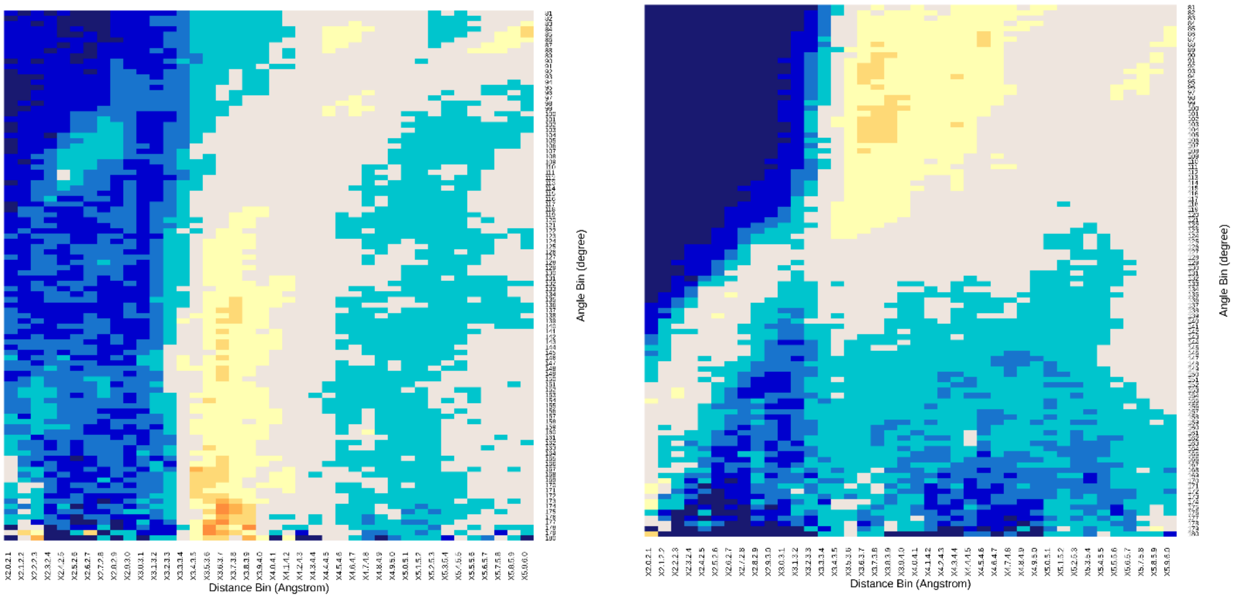

O) N, donor

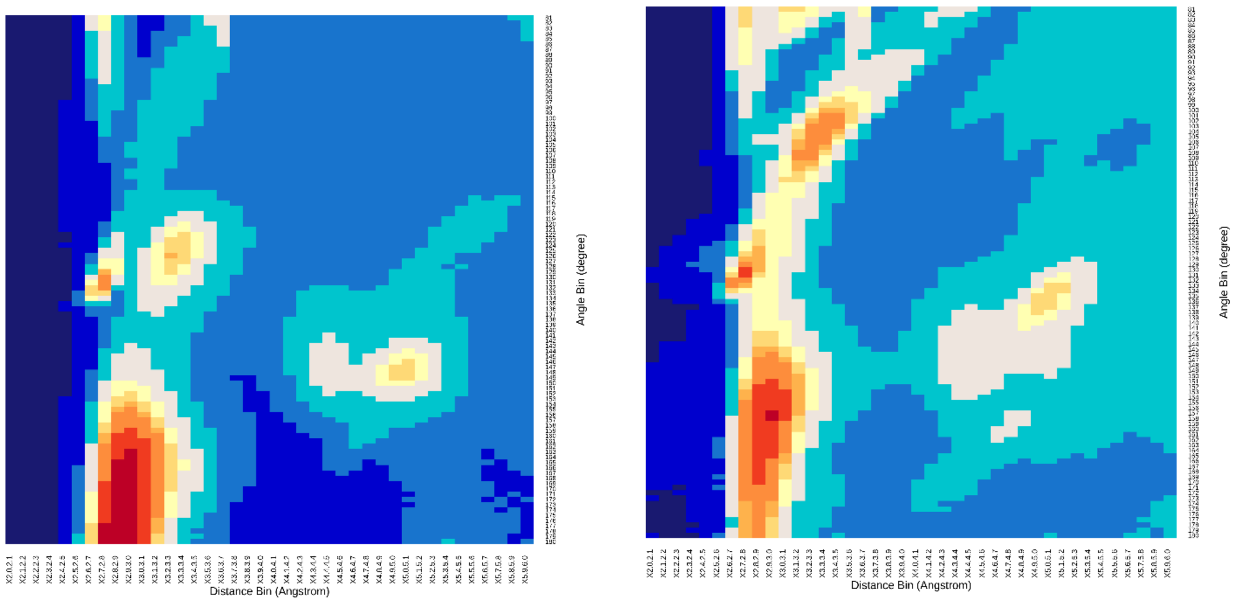

P) ND1, donor

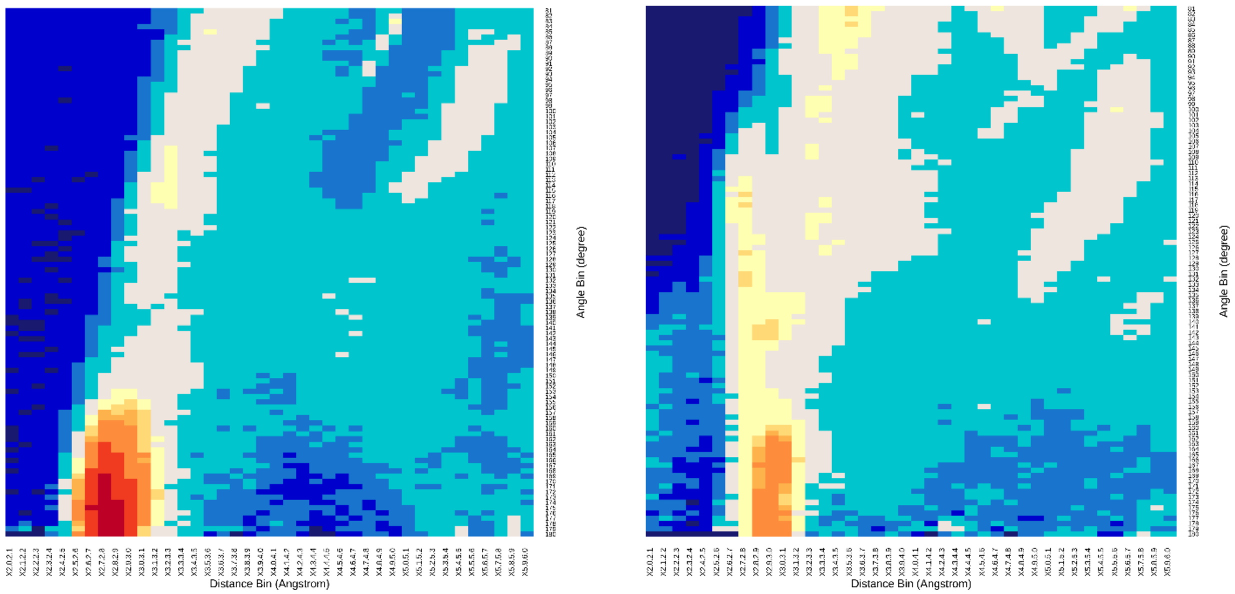

Q) ND2, donor

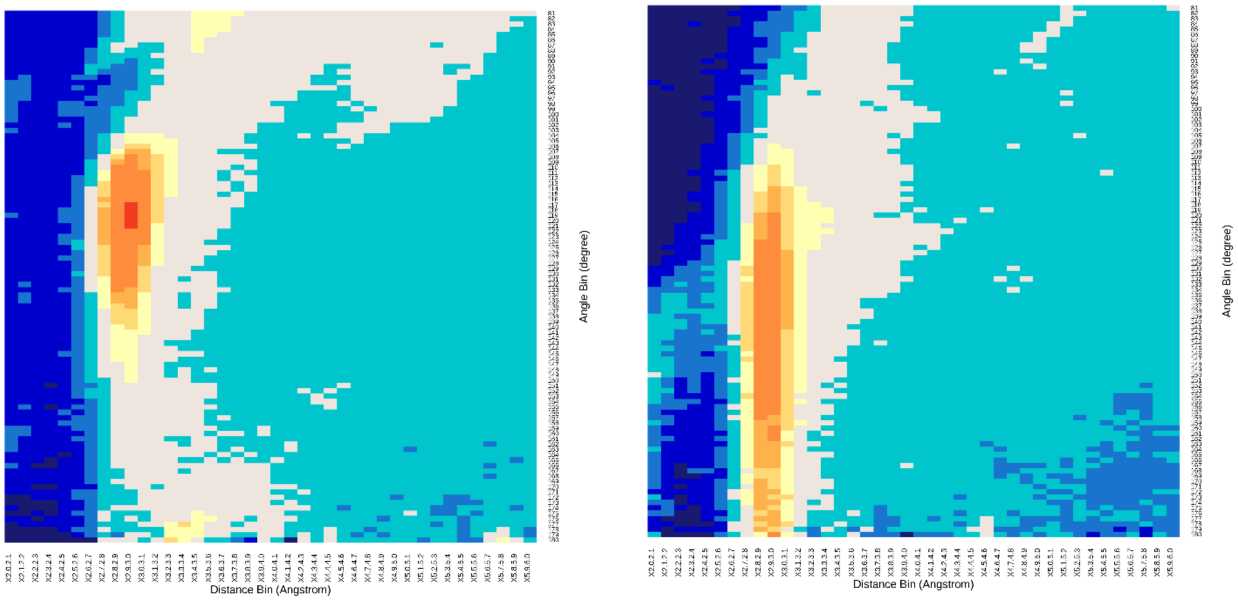

R) NE, donor

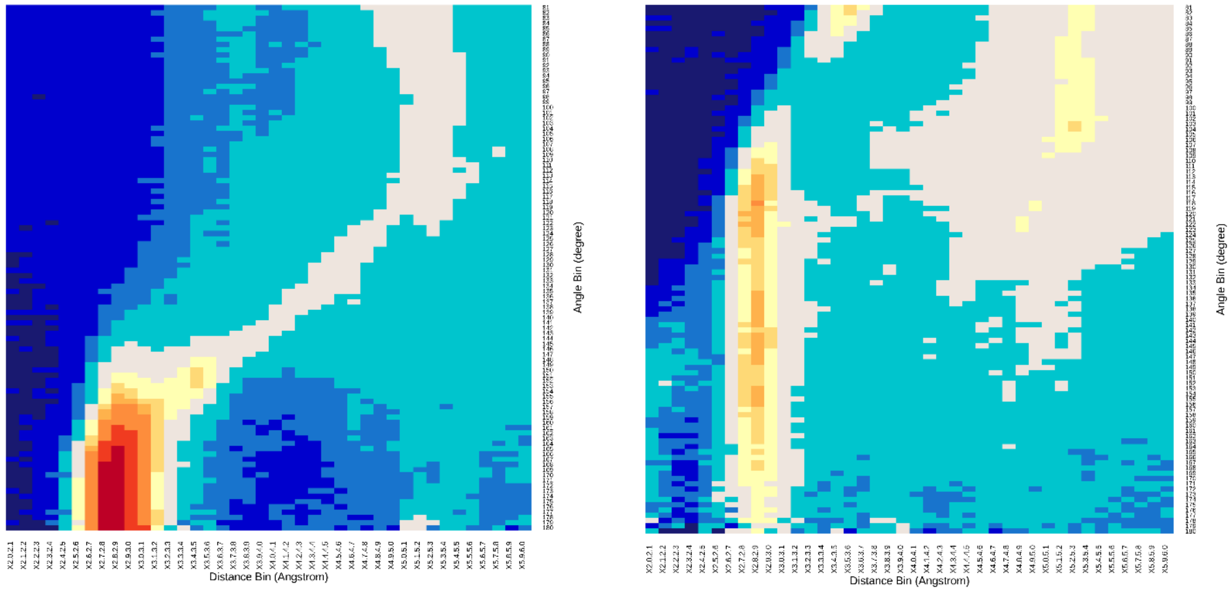

S) NE1, donor

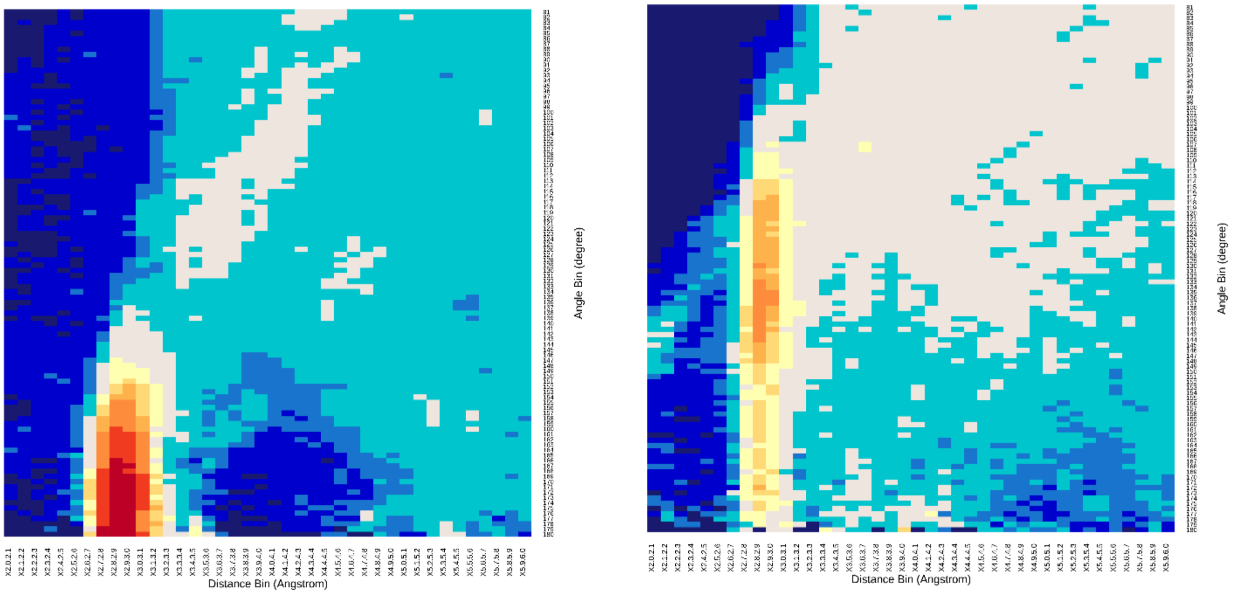

T) NE2, donor

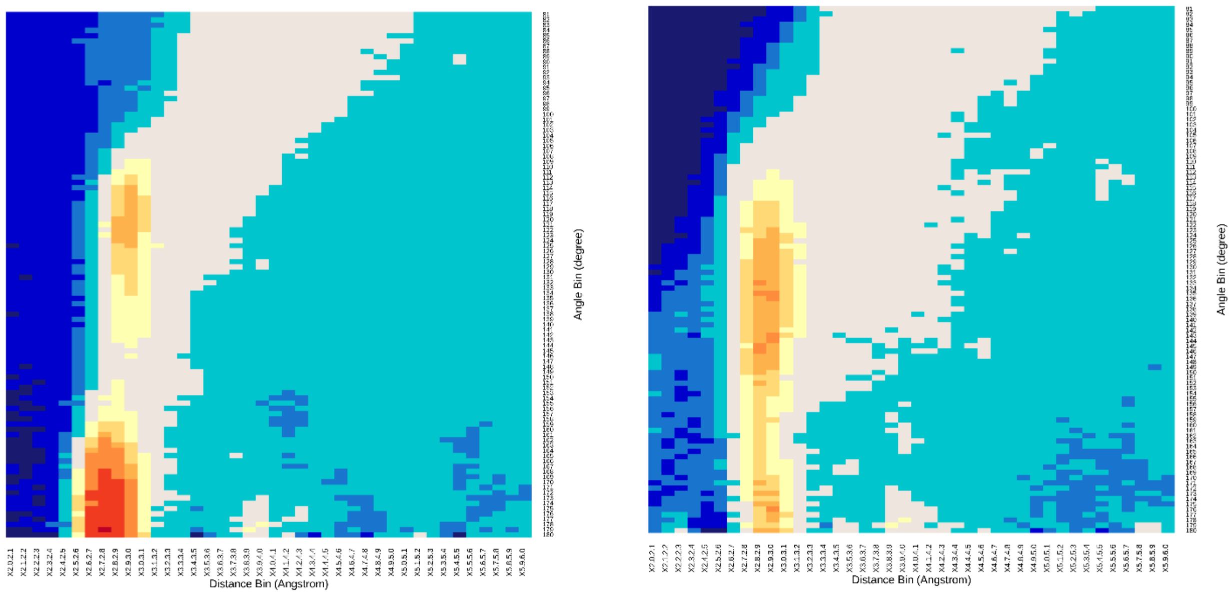

U) NH1, donor

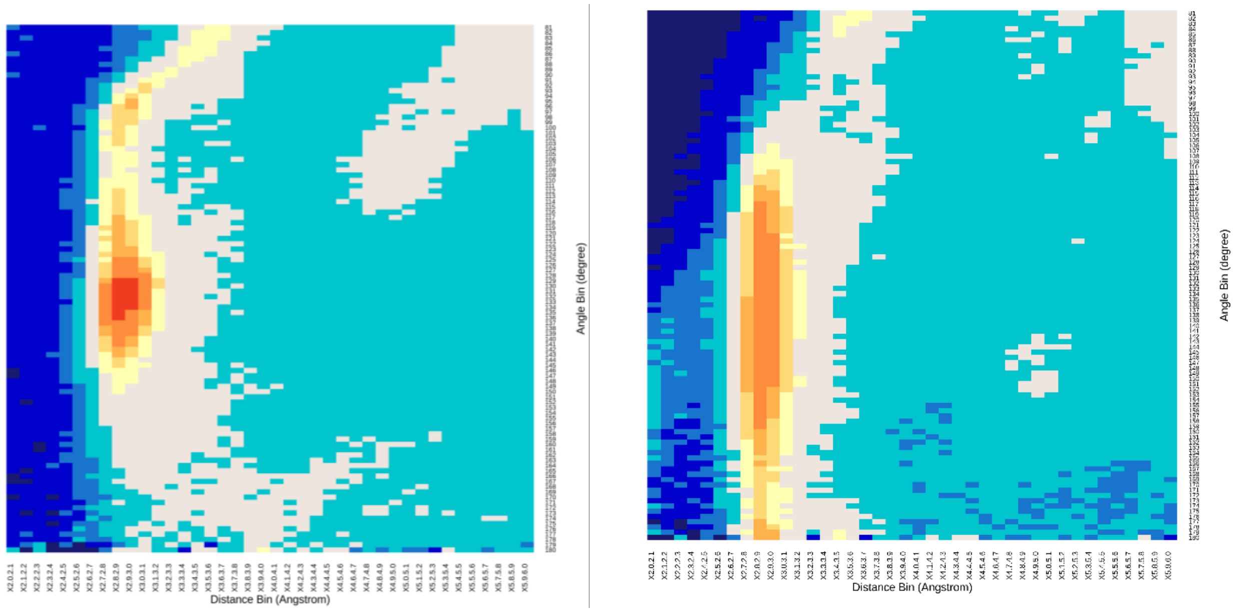

V) NH2, donor

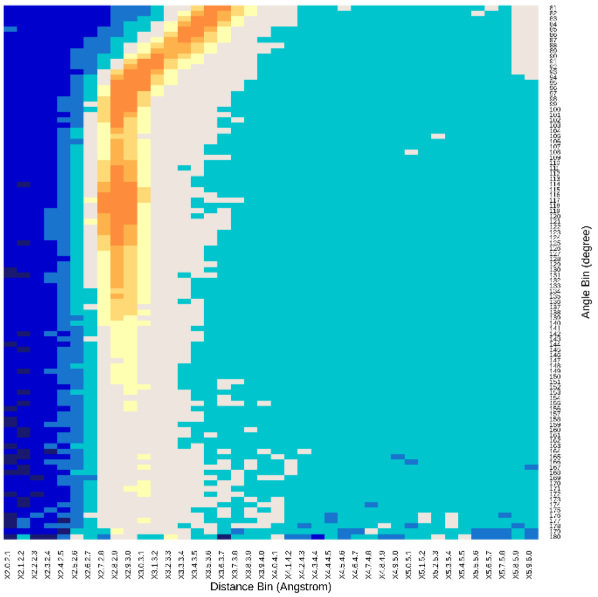

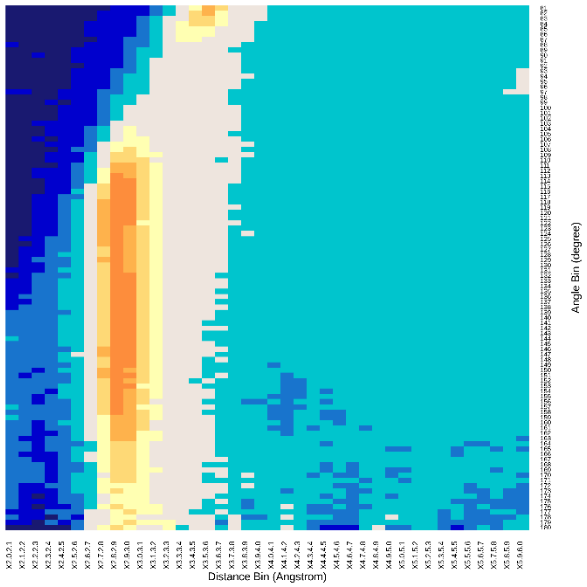

W) NZ, donor

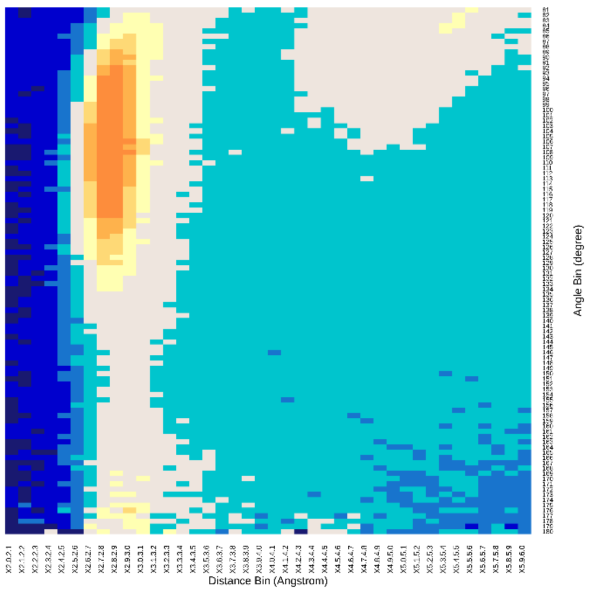

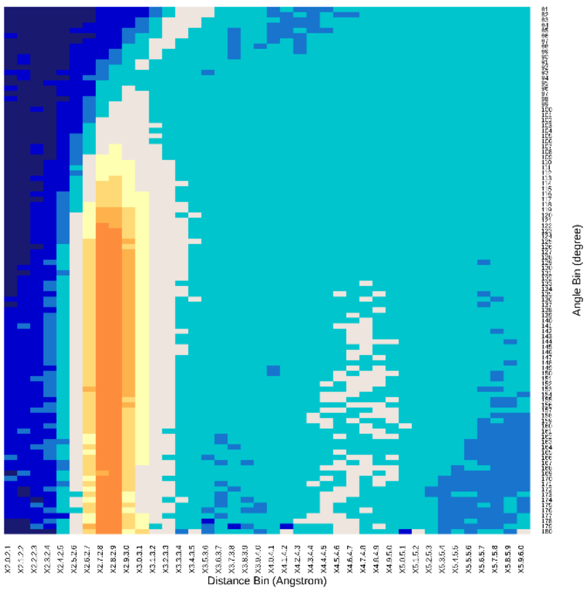

X) OD1, donor

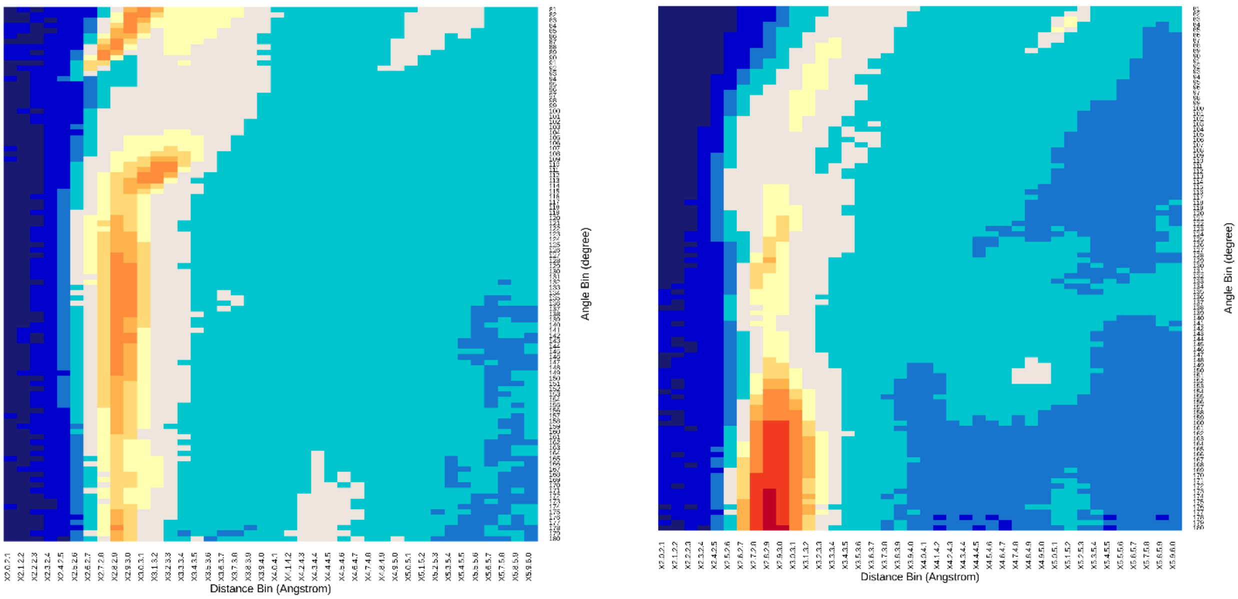

Y) OD2, donor

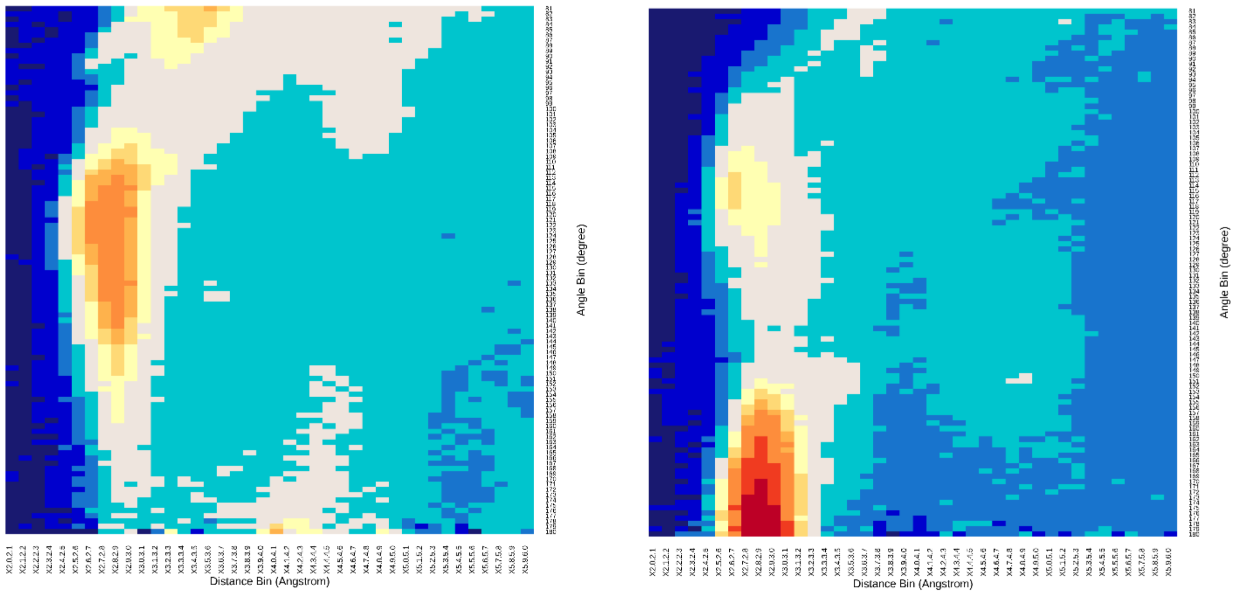

Z) OE1, donor

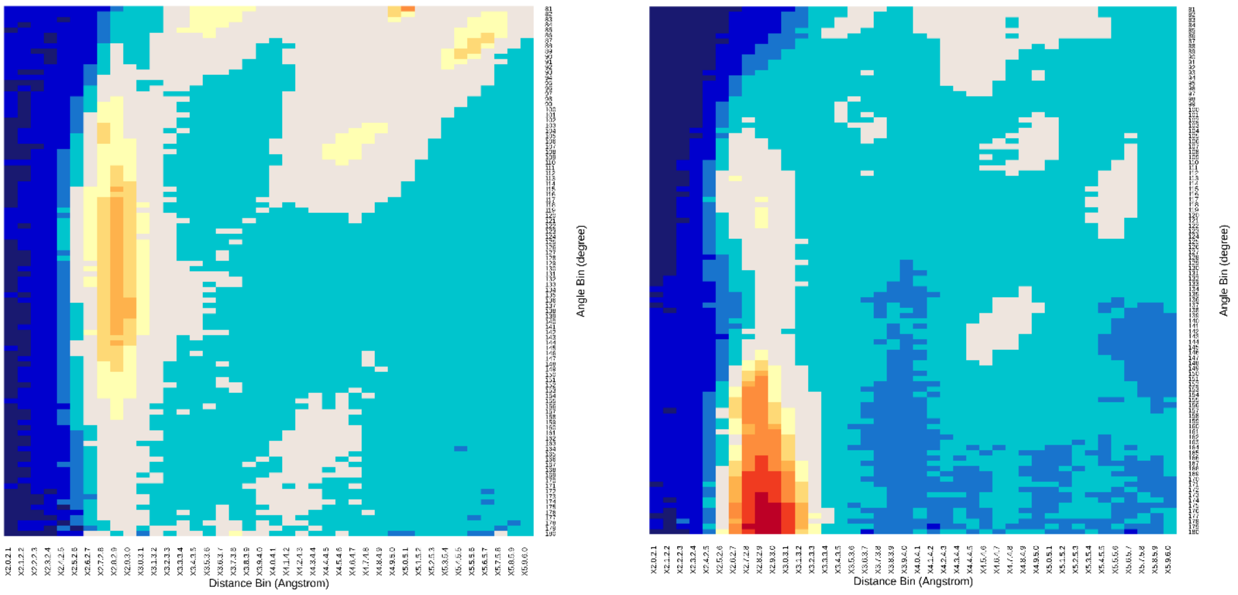

AA) OE2, donor

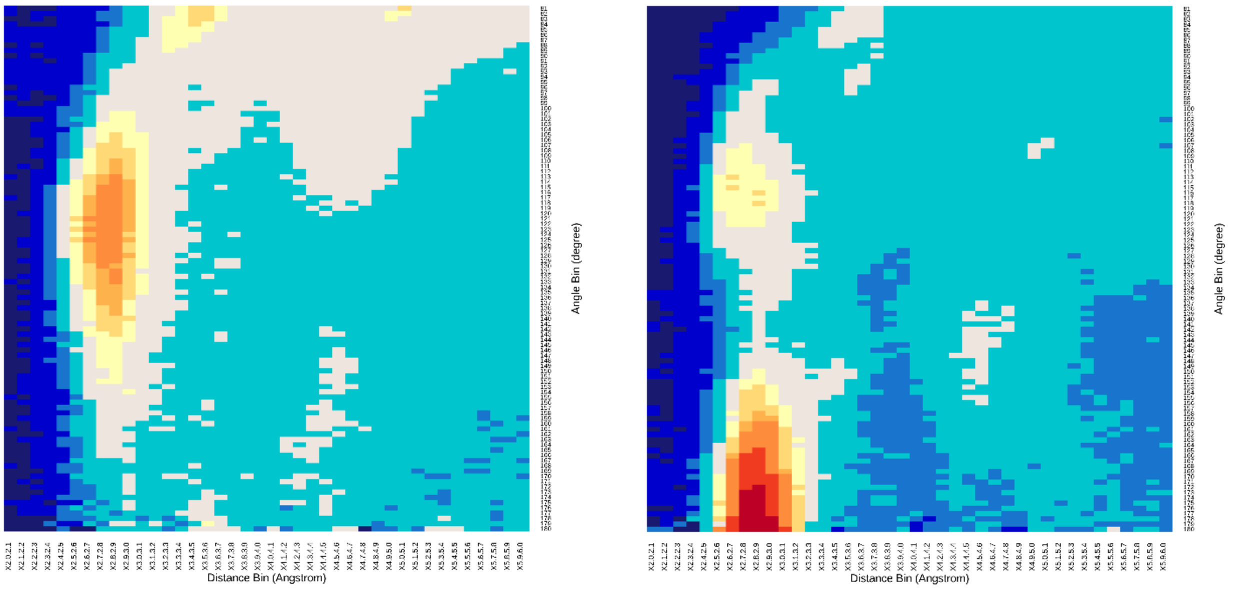

AB) OG, donor

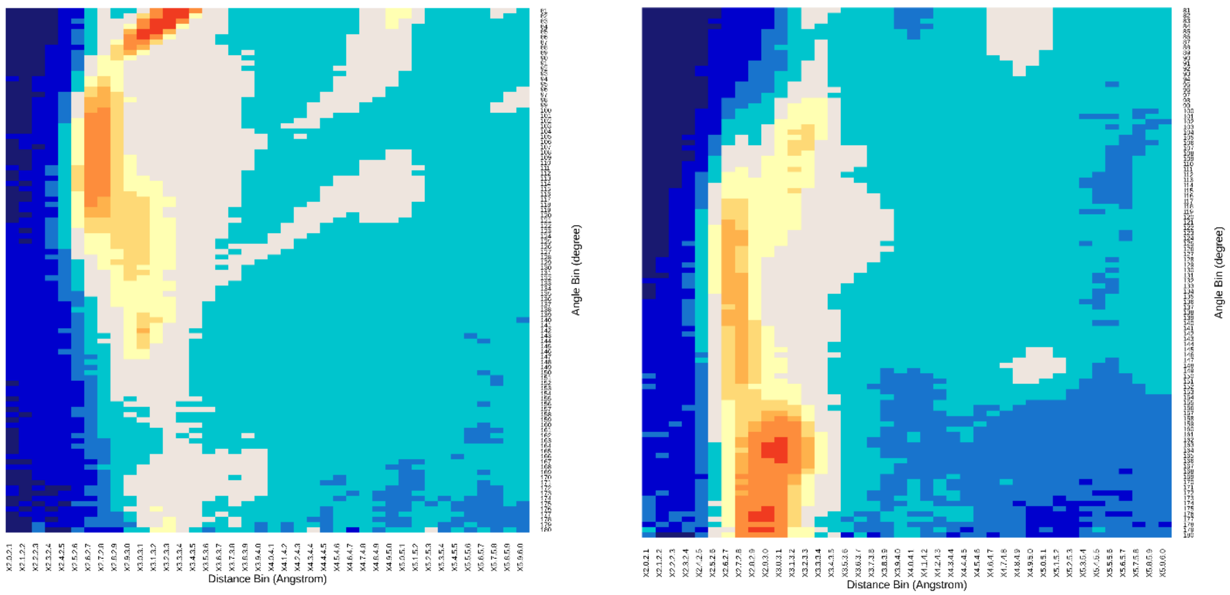

AC) OG1, donor

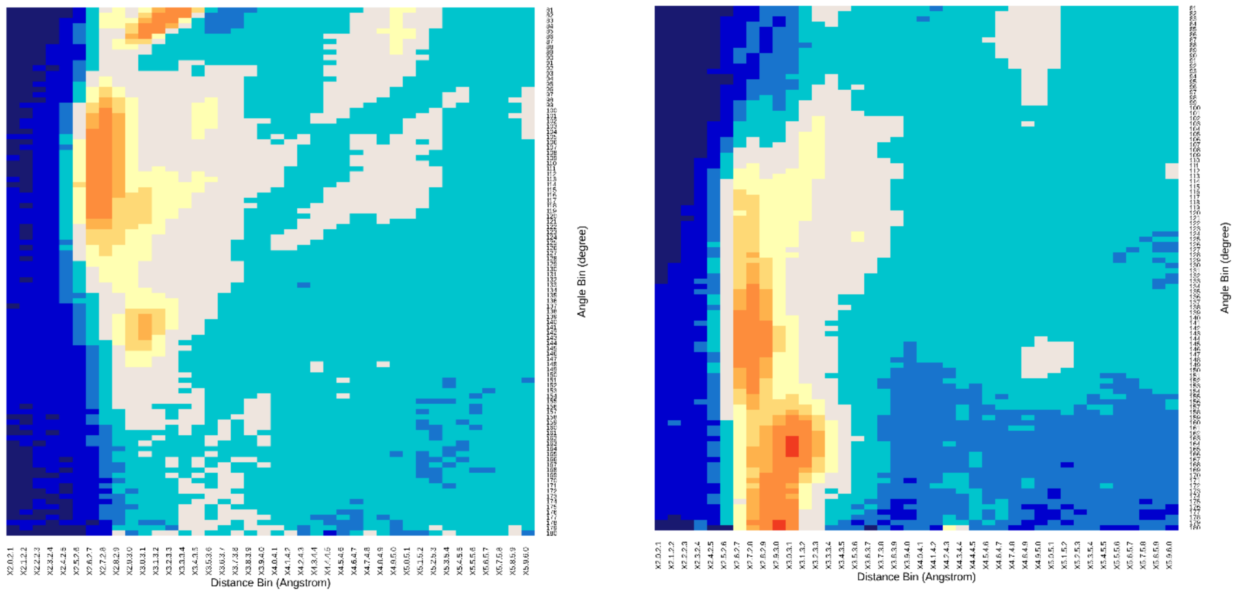

AD) OH, donor

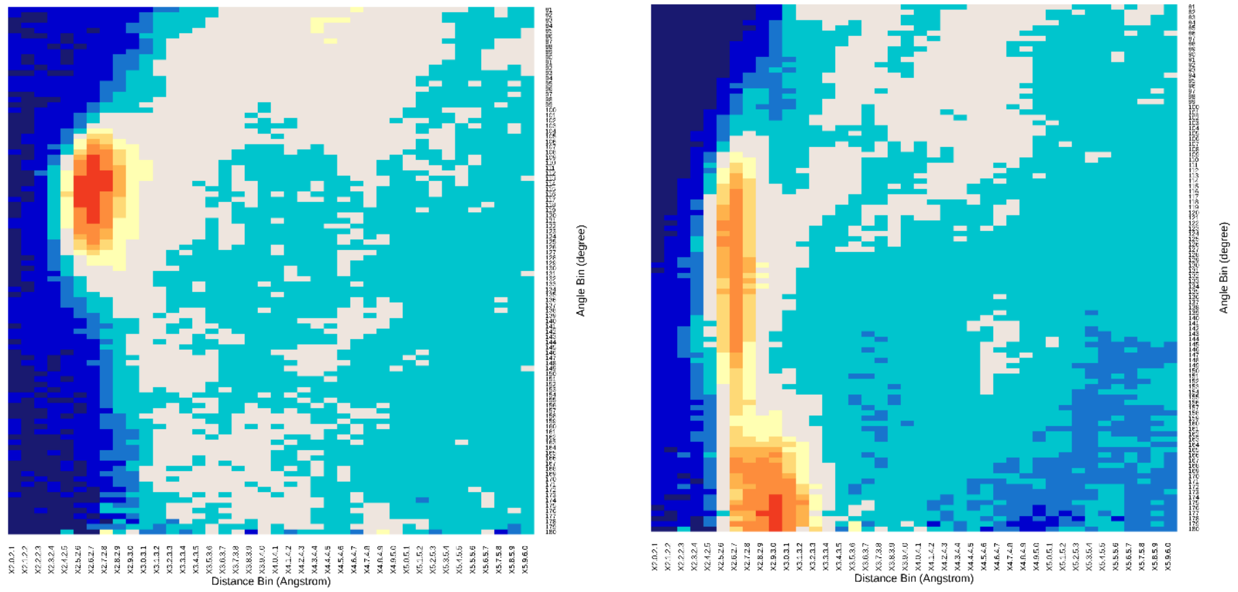

AE) SG, donor

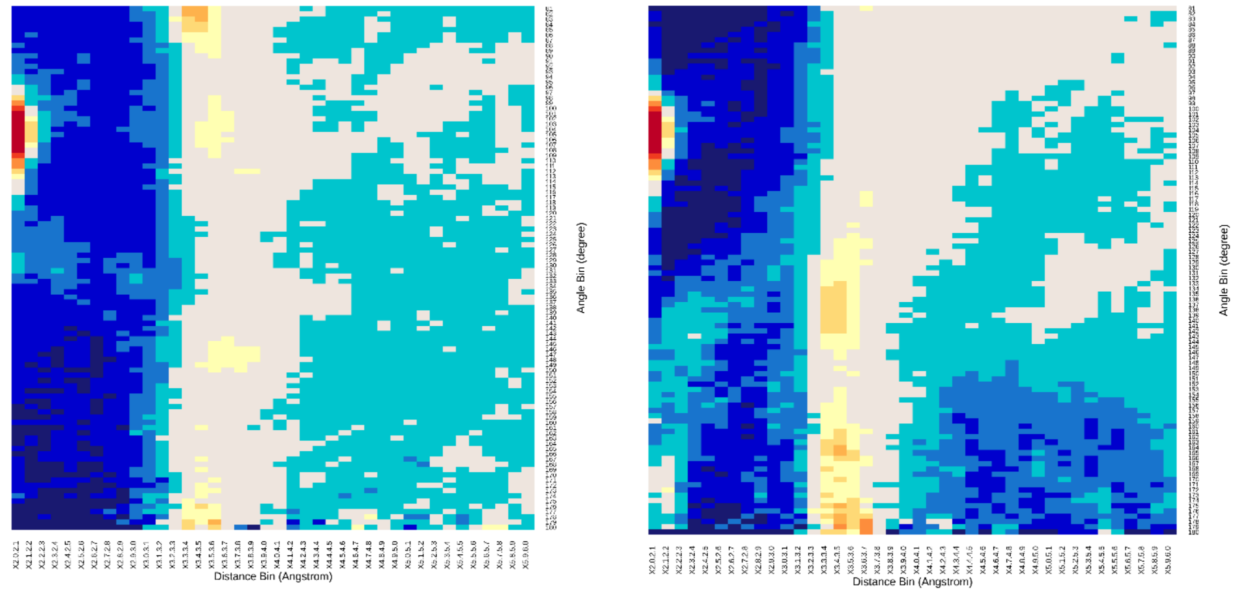

AF) CD2, acceptor

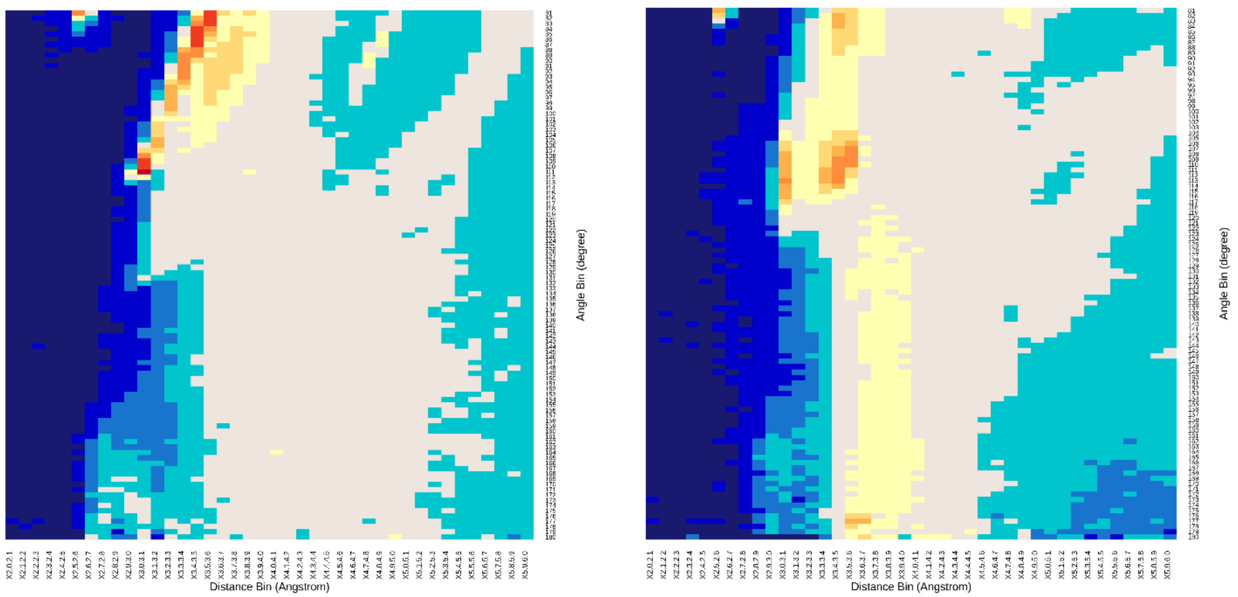

AG) CE1, acceptor

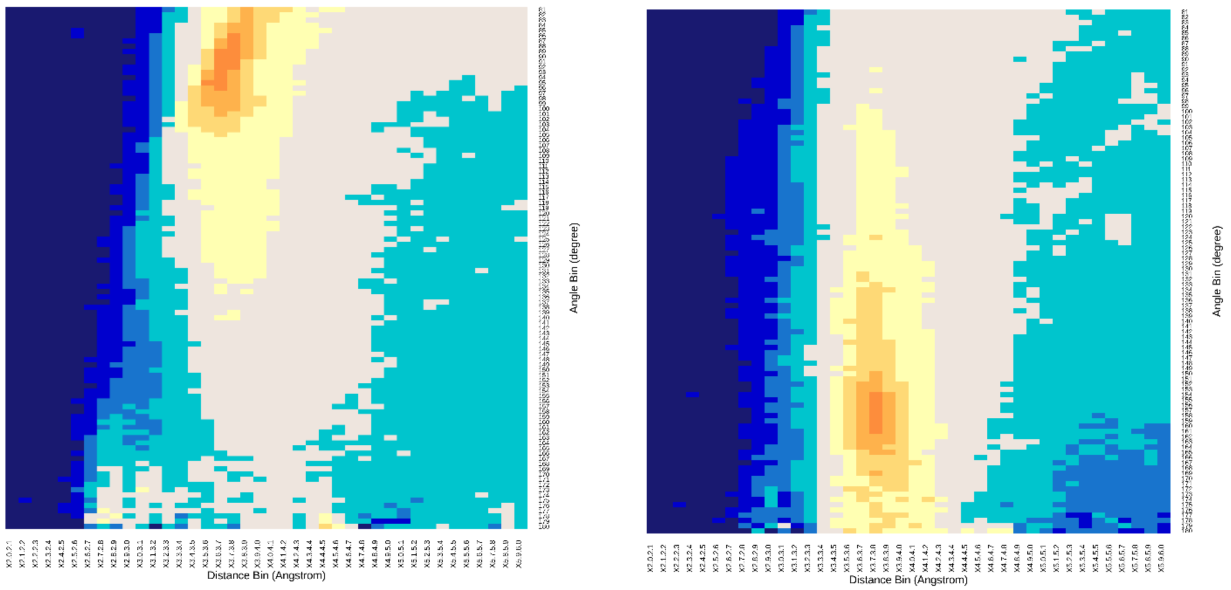

AH) N, acceptor

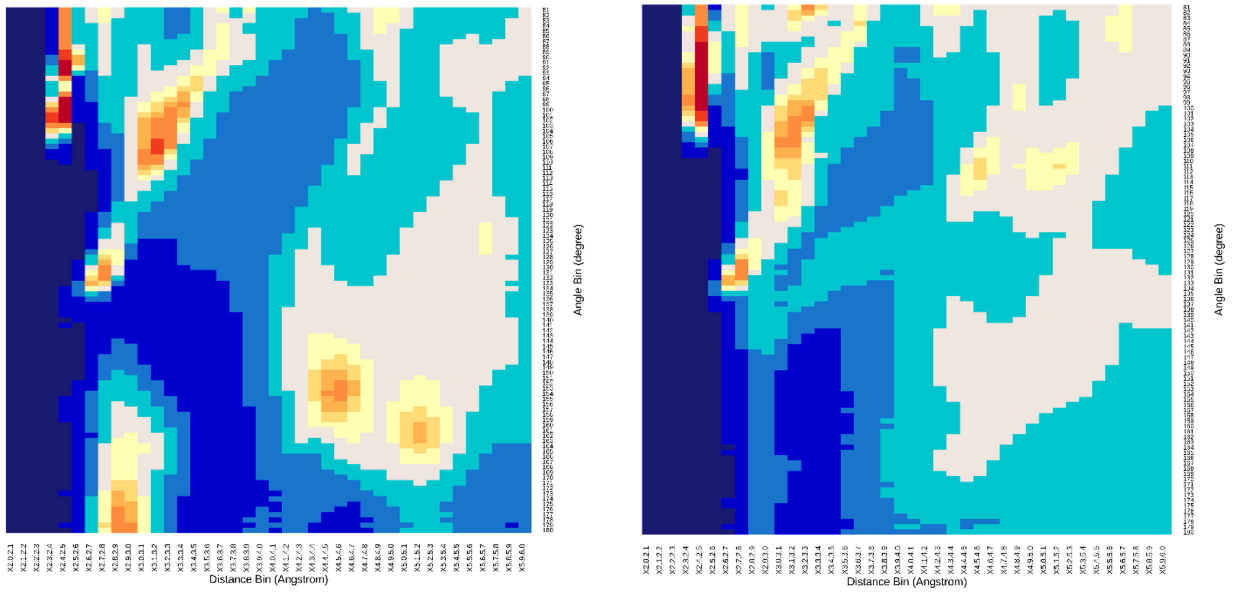

AI) ND1, acceptor

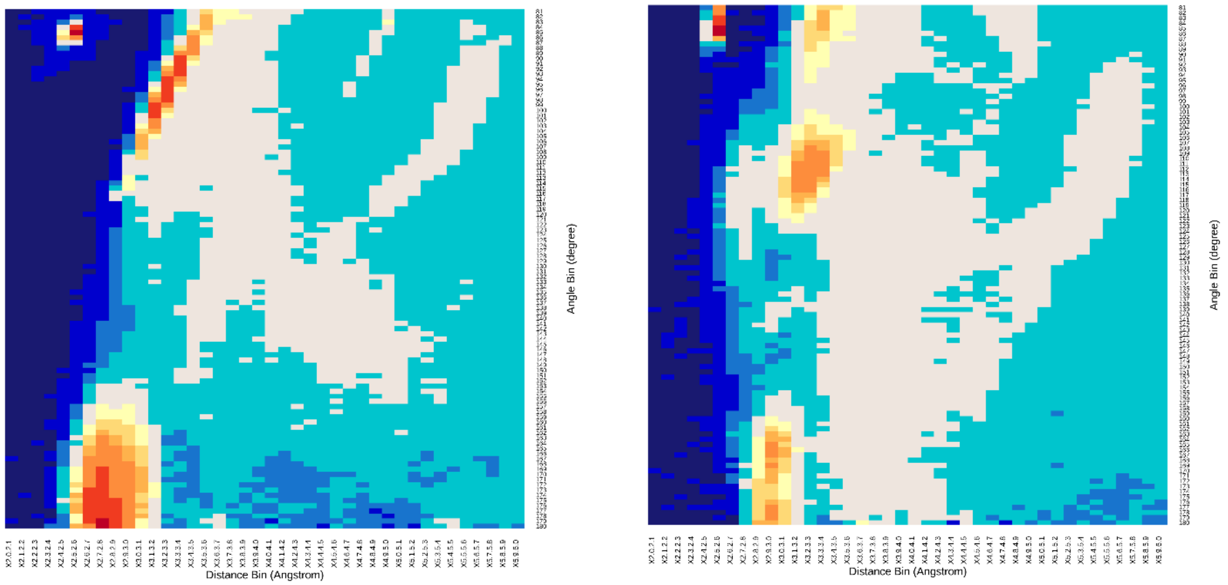

AJ) ND2, acceptor

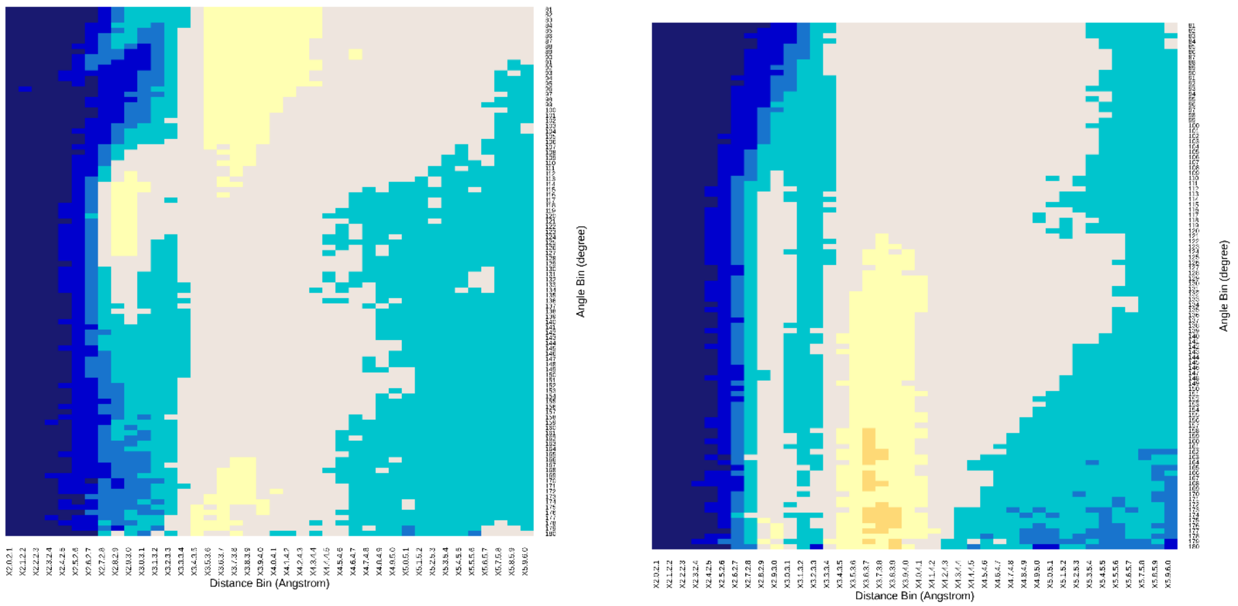

AK) NE, acceptor

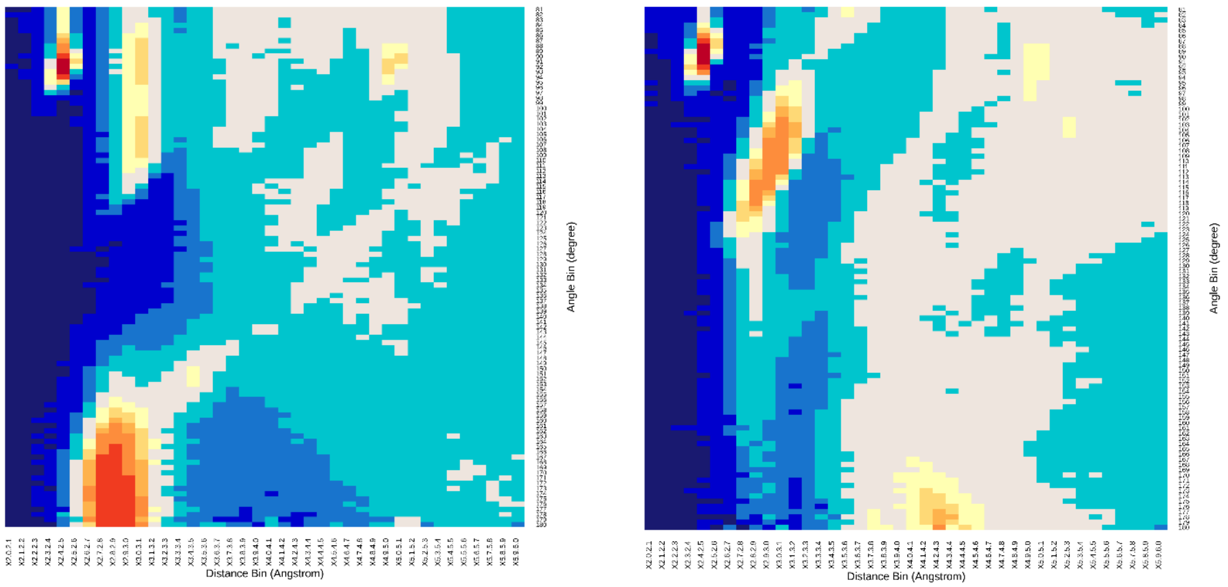

AL) NH1, acceptor

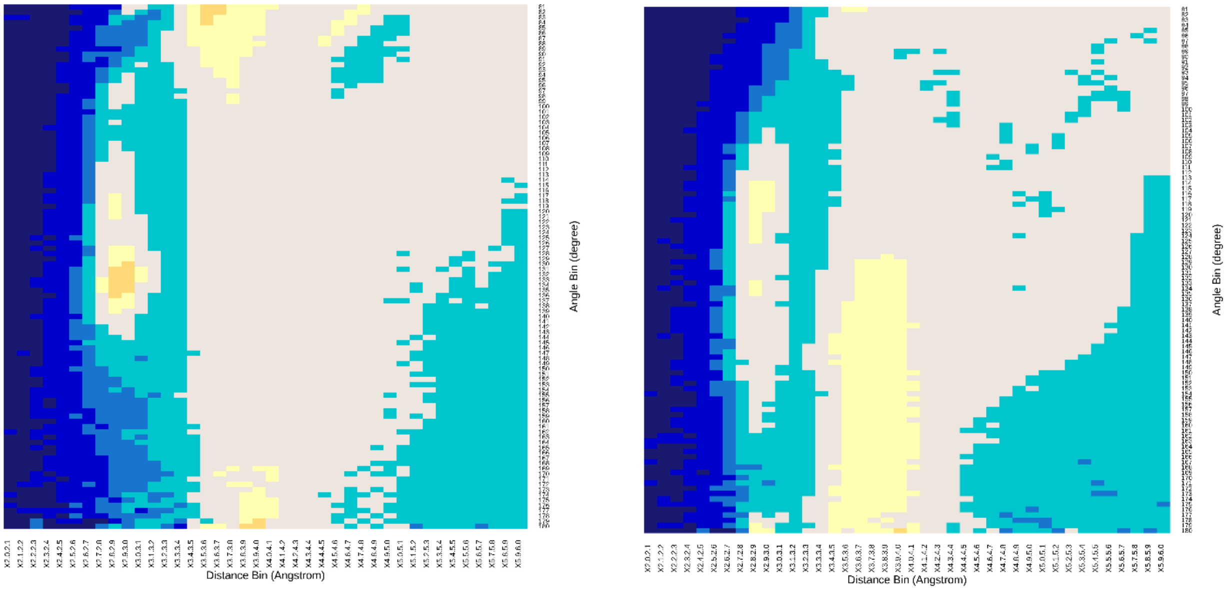

AM) NH2, acceptor

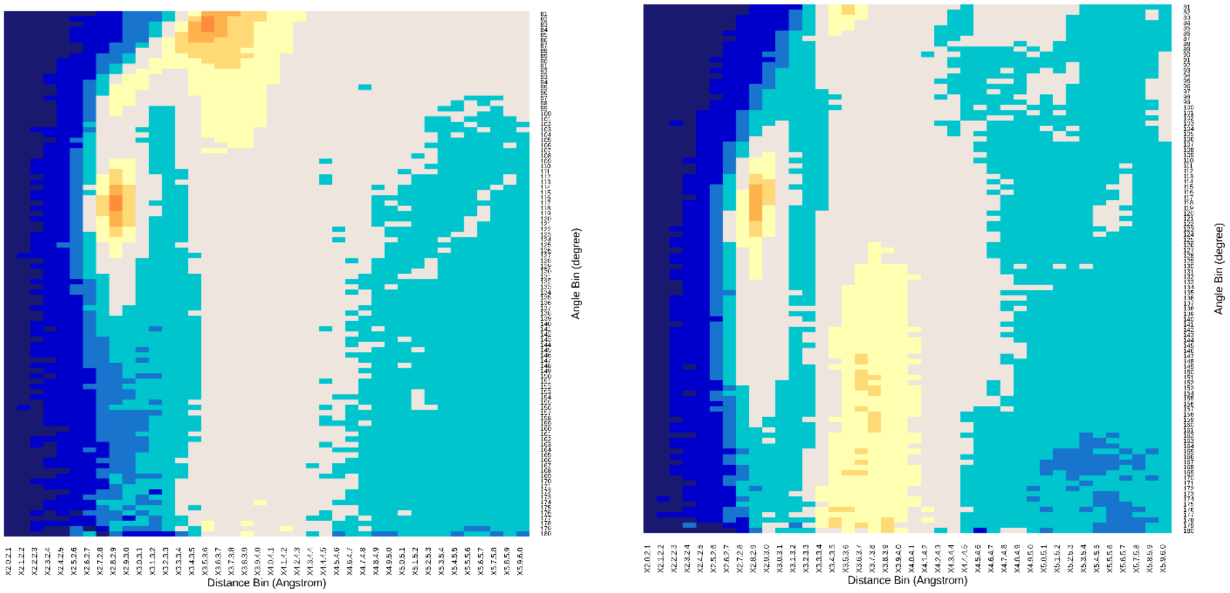

AN) NZ, acceptor

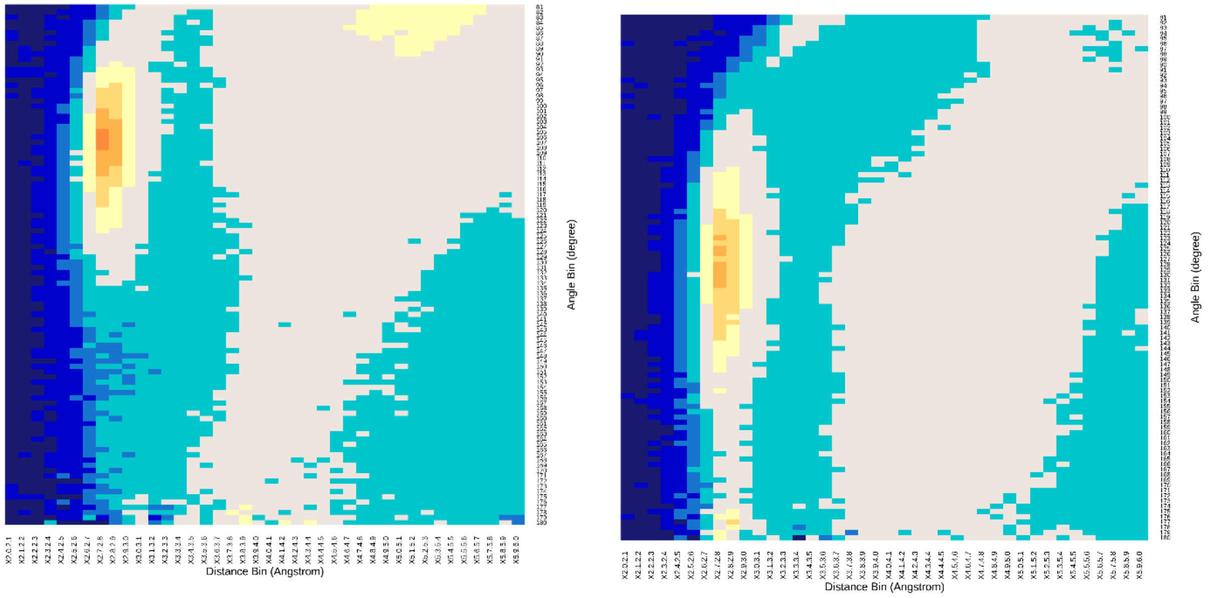

AO) O, acceptor

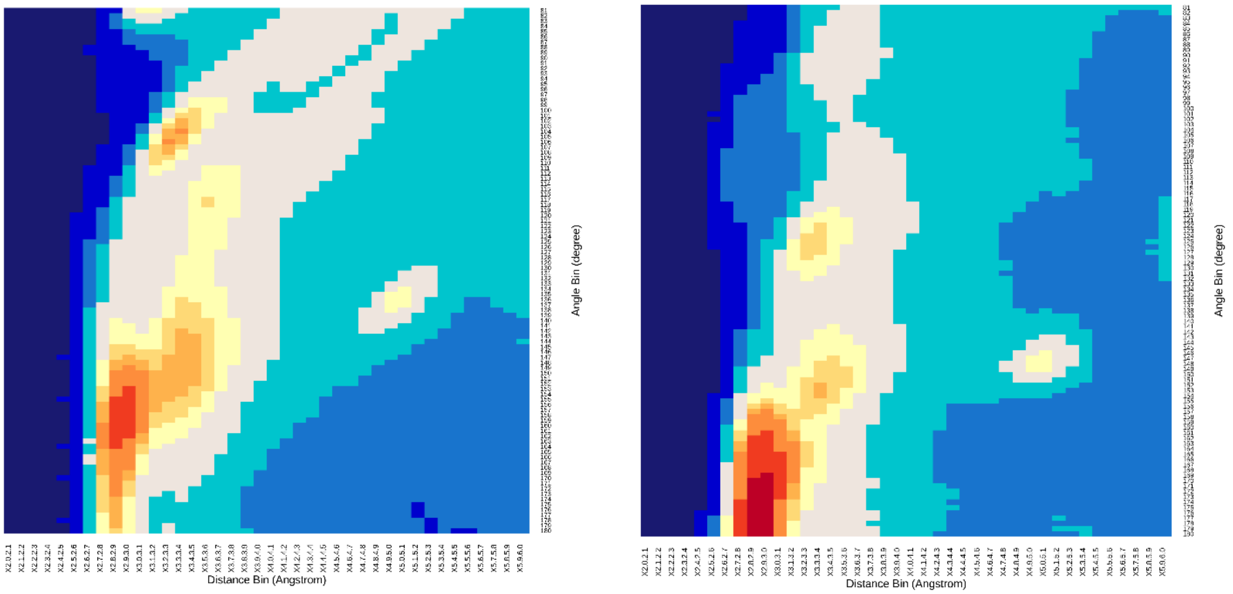

AP) OD1, acceptor

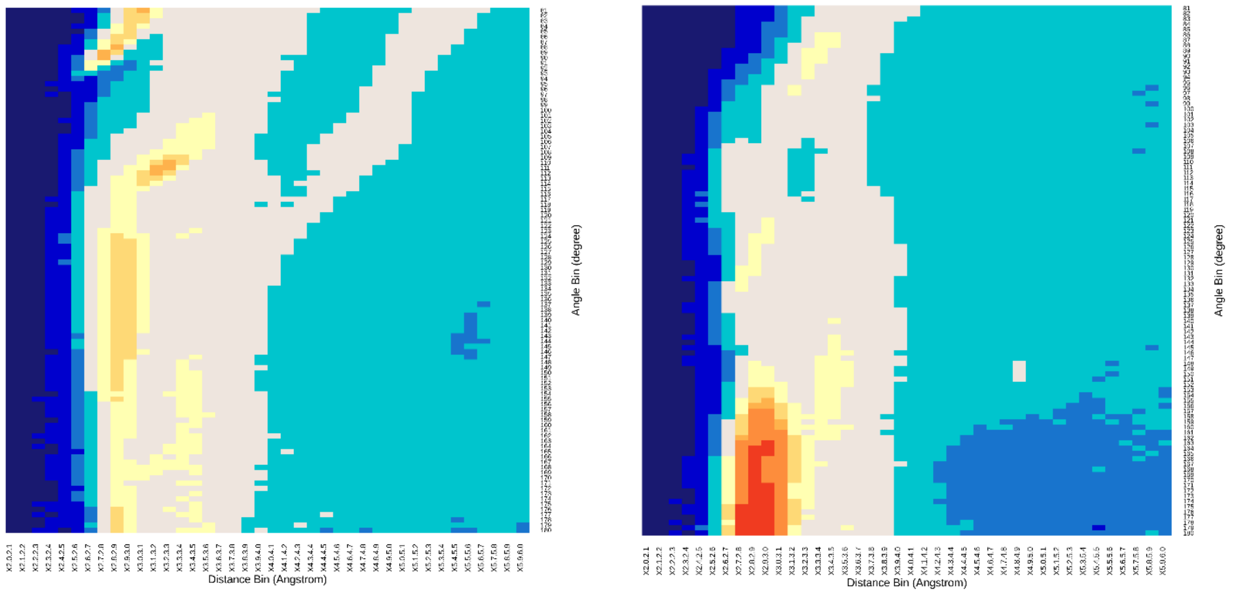

AQ) OD2, acceptor

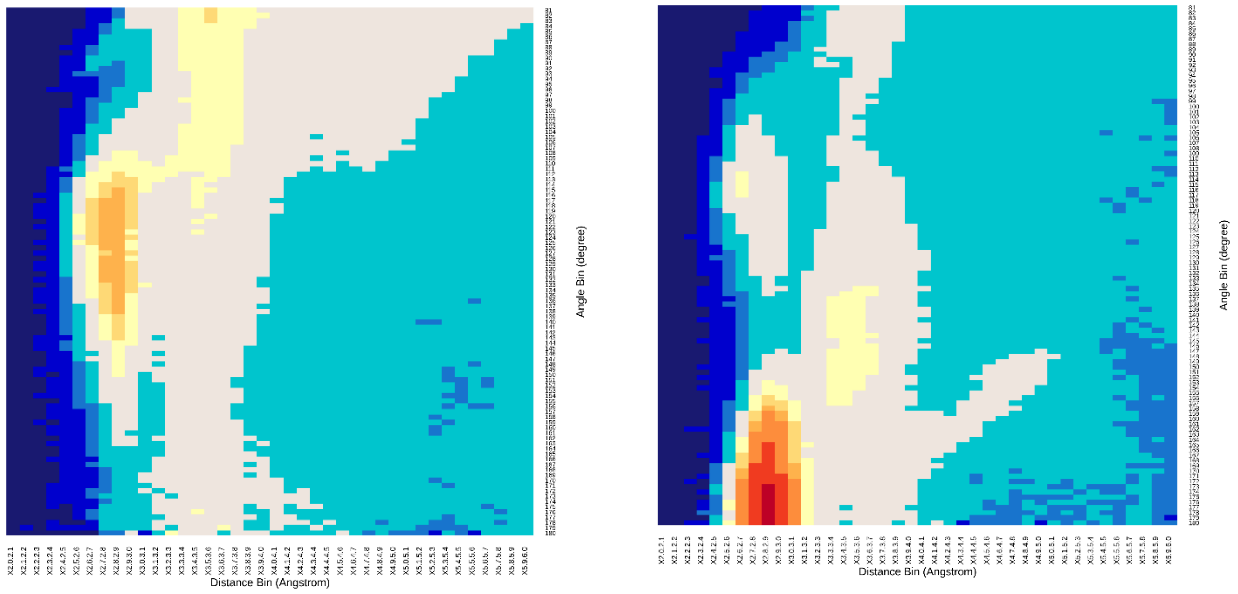

AR) OE1, acceptor

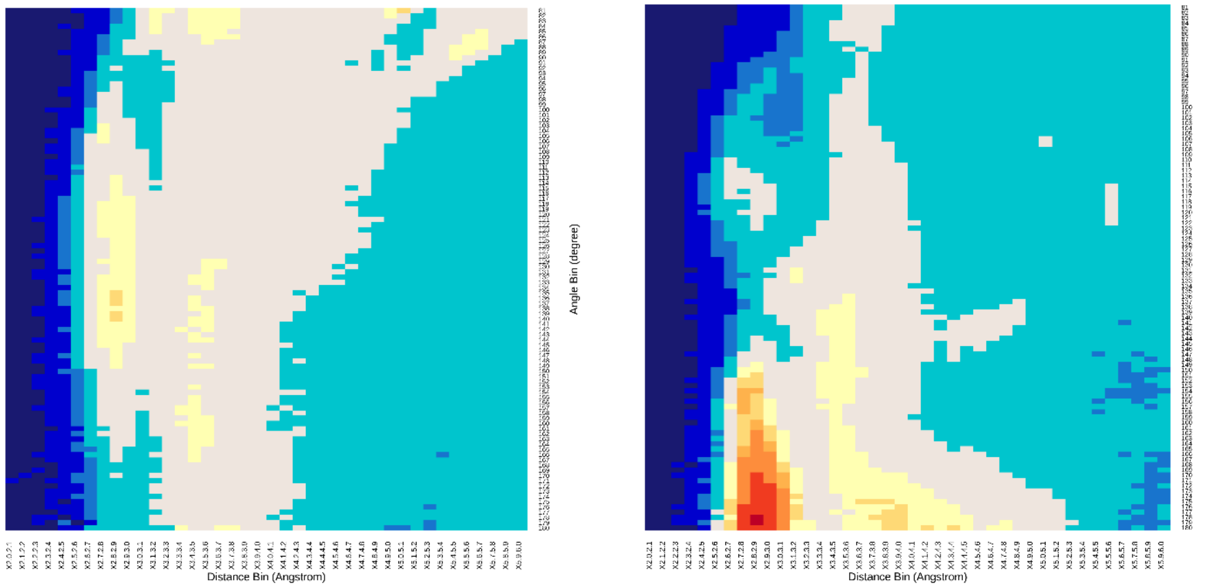

AS) OE2, acceptor

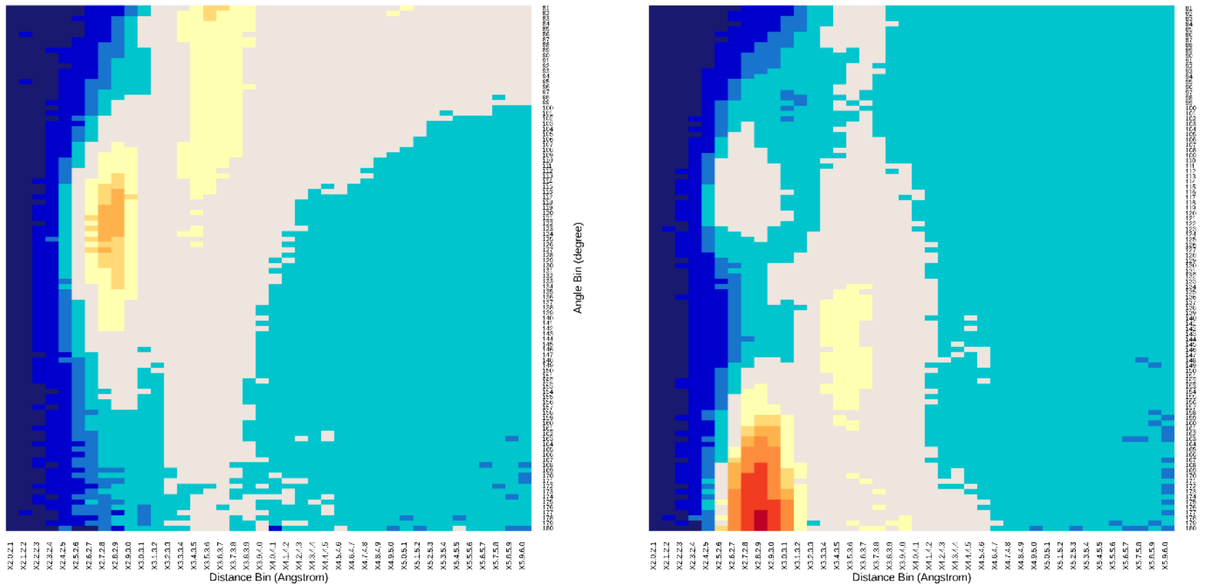

AT) OG, acceptor

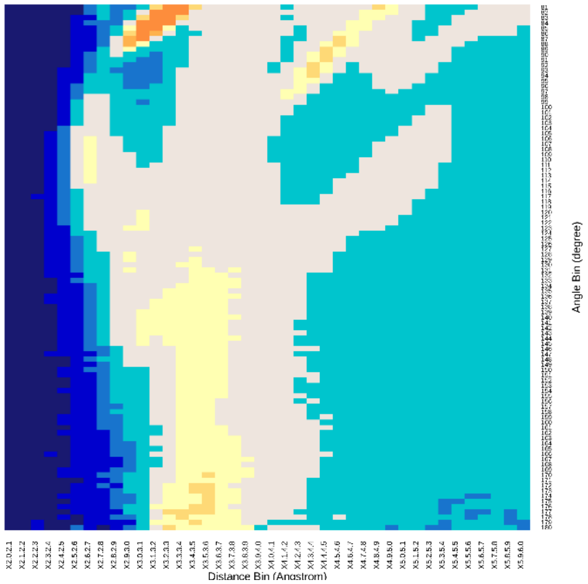

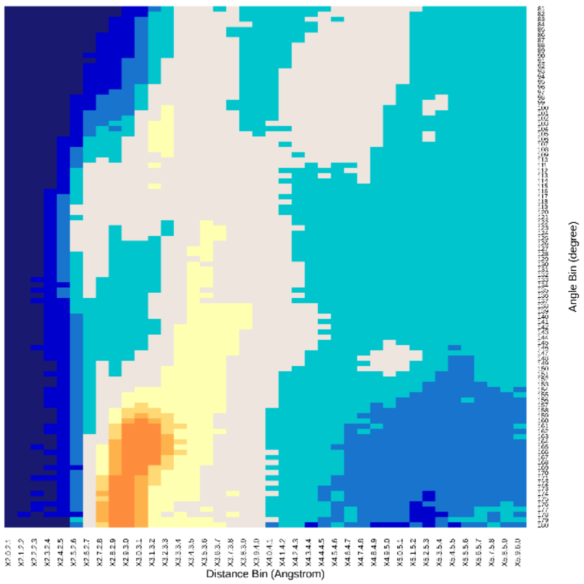

AU) OG1, acceptor

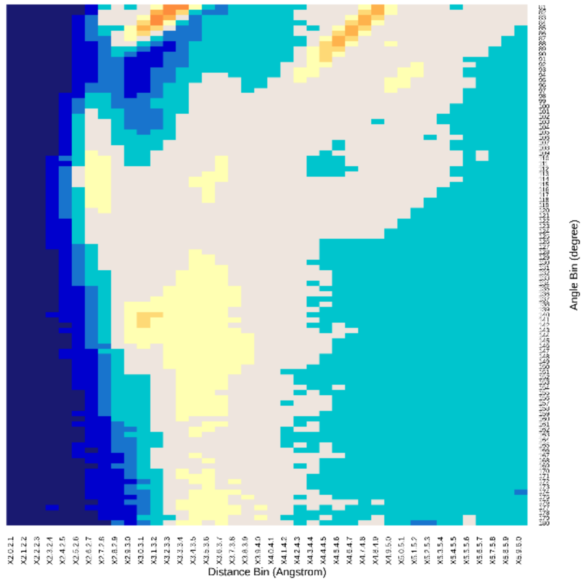

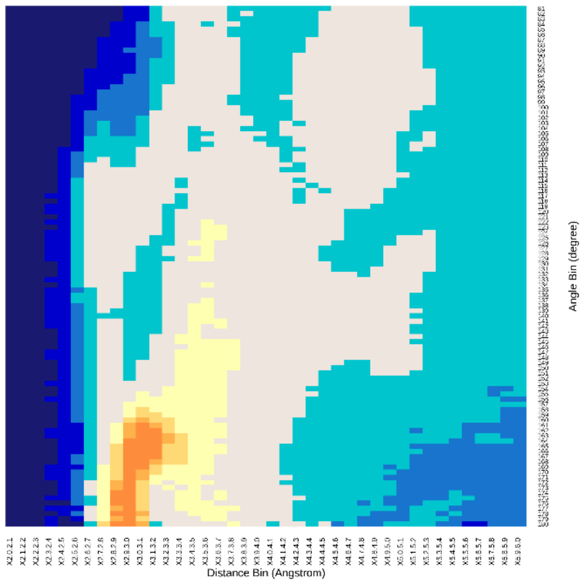

AV) OH, acceptor

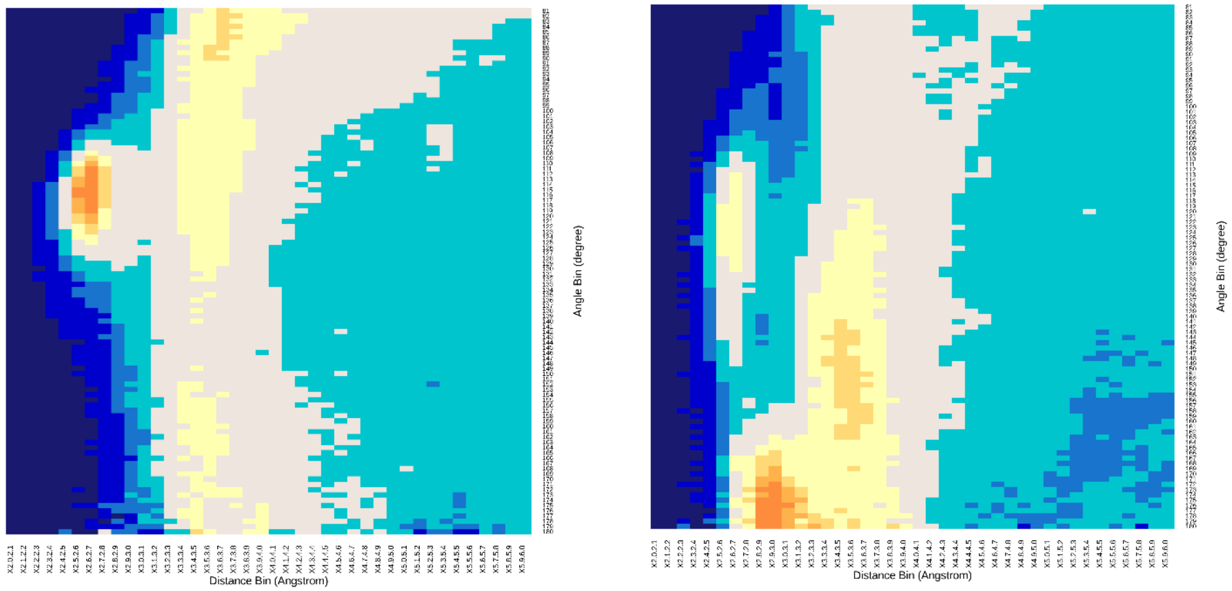

AW) SD, acceptor

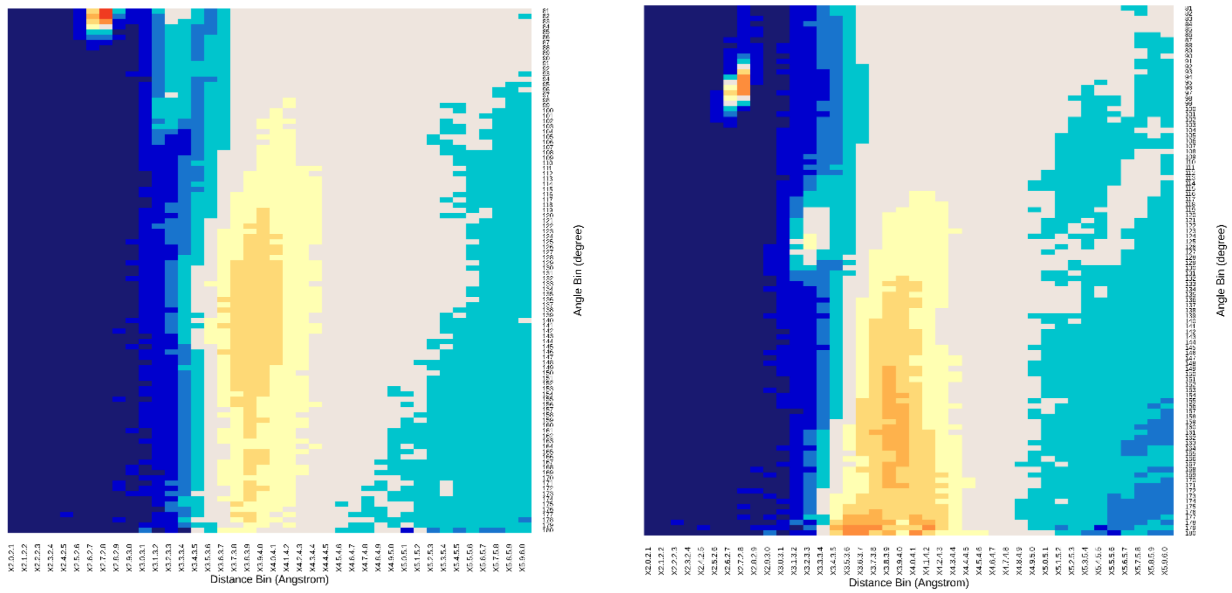

AX) SG, acceptor

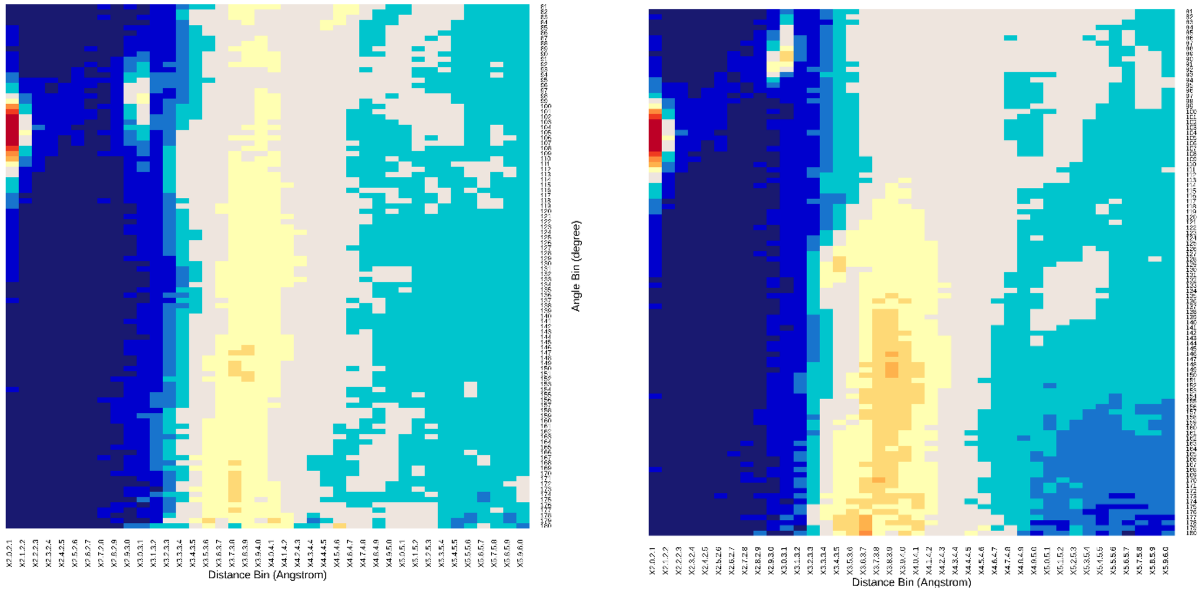

AY) X1 (Phe), acceptor

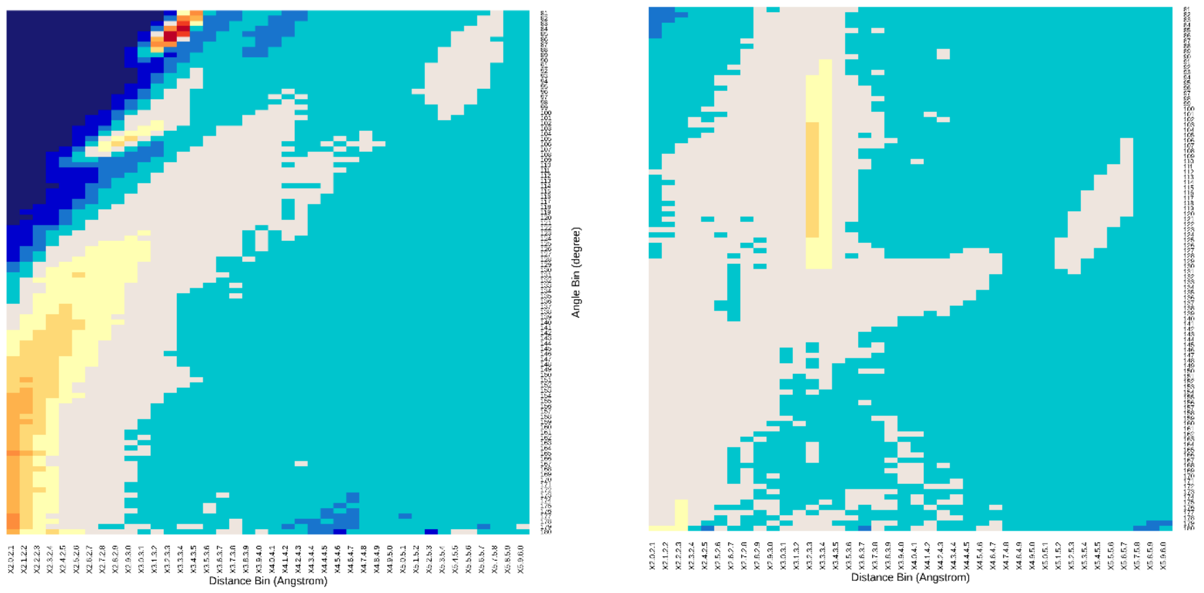

AZ) X2 (His), acceptor

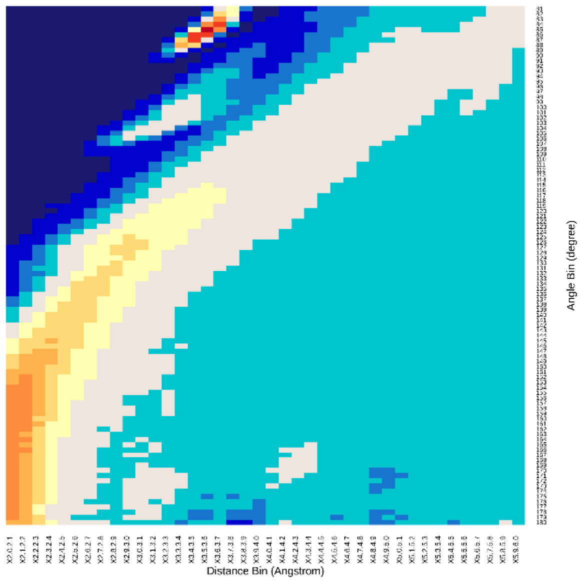

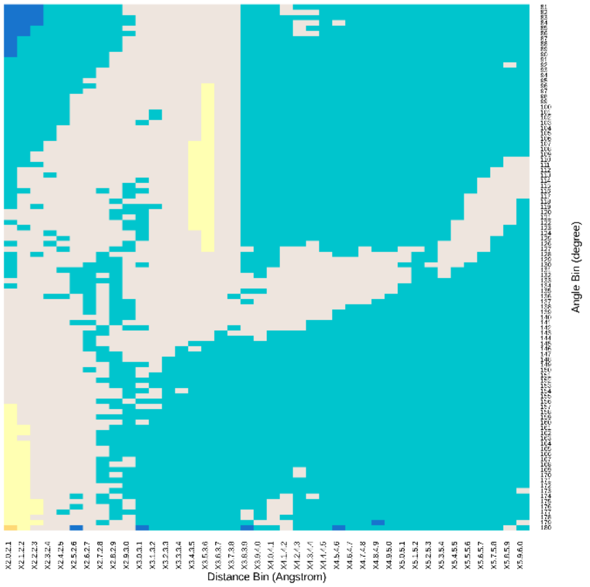

BA) X3 (Tyr), acceptor

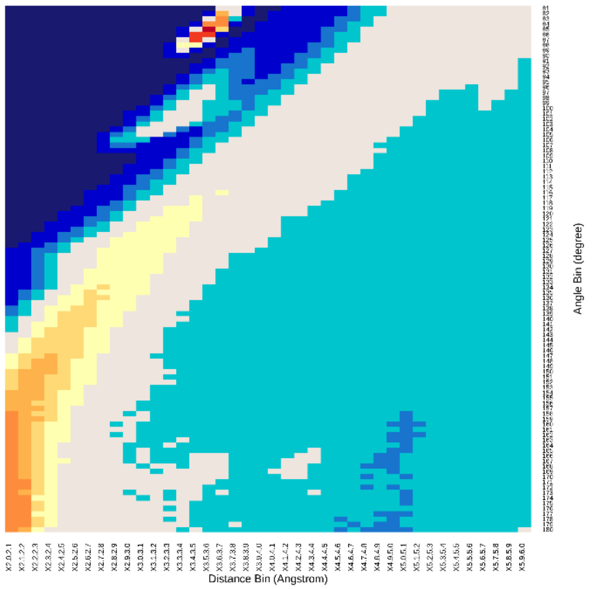

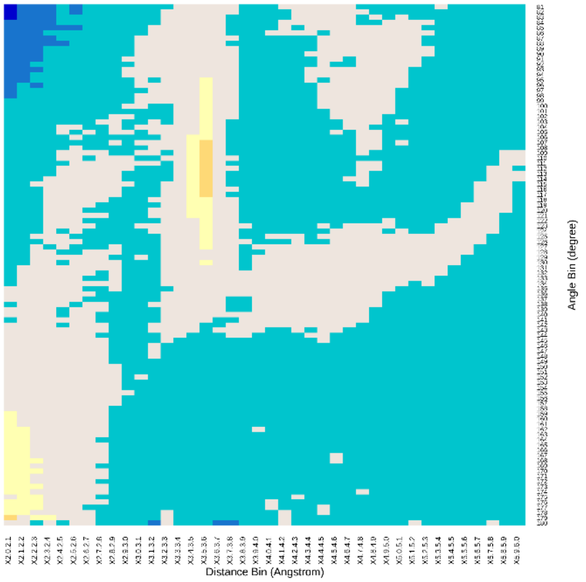

BB) X4 (Trp, 5 member), acceptor

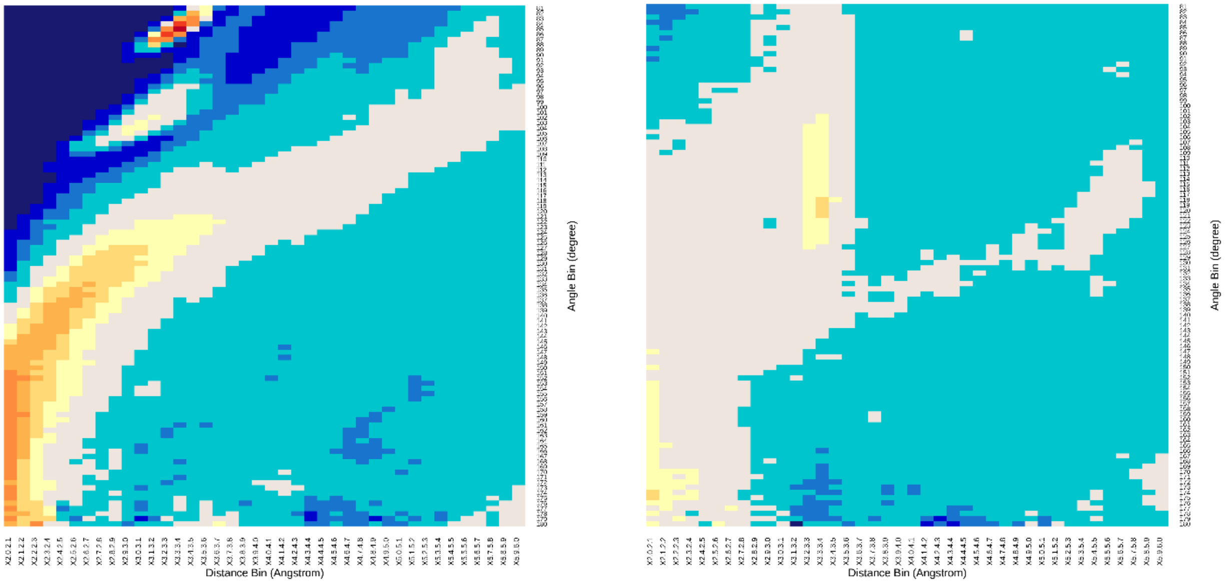

BC) X5 (Trp, 6 member), acceptor

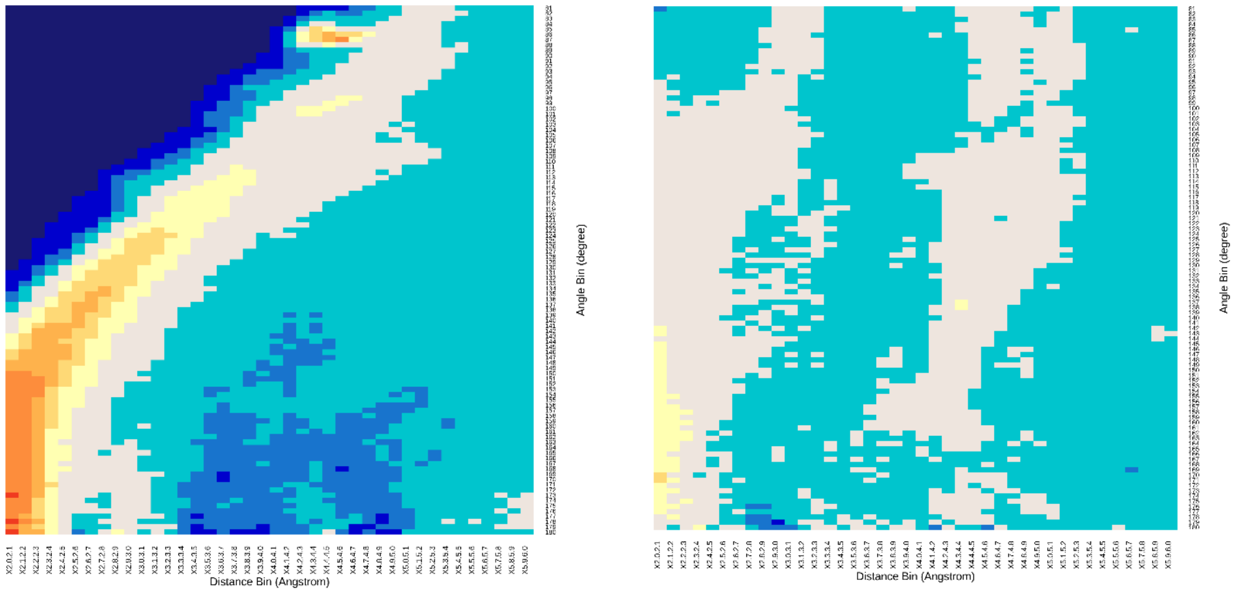

SI Figure 2: Example geometries for each of the HMI. Example geometries from the PDB for each of the HMI given in Table 1 of the main text for the direct and indirect angle of each are given where appropriate. Atoms are shown as sticks with carbon in grey, oxygen in red, nitrogen in blue, sulfur as dark yellow, and hydrogen as light yellow. The atoms involved in the hydrogen mediated interaction are further identified with an additional semi-transparent sphere of the appropriate color. In all cases, an example of the direct version of the interaction is shown on the left and the indirect formulation on the right. **A)** N atom type, donor, direct: PDB ID 2g3d, Glu32/Lys45, indirect: PDB ID 1h6r, Glu34/Thr43
B) NE atom type, donor, direct: PDB ID 1w7s, Arg122/Glu115, indirect: PDB ID 7lzm, Arg96/Leu91
C) NE1 atom type, donor, direct: PDB ID 2fzu, Trp57/Asp216, indirect: PDB ID 1jyv, Trp203/Gln573
D) ND1 atom type, donor, direct: PDB ID 2g5z, His199/Asp197, indirect: PDB ID 5dtx, His82/Asp198
E) OD1 atom type, donor, direct PDB ID 2qle, Asp210/Asn212, indirect: PDB ID 3dpz, Asp103/Lys131
F) NH2 atom type, donor, direct: PDB ID 1s6z, Arg122/Glu115, indirect: PDB ID 1l84, Arg145/Asn101
G) OD2 atom type, donor, direct: PDB ID 3l2x, Asp89/Lys85, indirect: 2ye1, Asp216/Leu53
H) NZ atom type, donor, direct: PDB ID 3gj2, Lys209K/His217, indirect: PDB ID 2ydz, Lys85/Ala72
I) NE2 atom type, donor, direct: PDB ID 1yjf, Gln177/Lys101, indirect: PDB ID 4l12, His77/Ile229
J) ND2 atom type, donor, direct: PDB ID 5dpg, Asn39/Asp36, indirect: PDB ID 1q4b, Asn135/Gln177
K) OH atom type, donor, direct: PDB ID 2okw, Tyr74/His199, indirect: PDB ID 3an1, Tyr708/Gly868
L) OE2 atom type, donor, direct: PDB ID 3dq1, Glu222/Ser205, indirect: PDB ID 2iea, Glu232/Gly195
M) NH1 atom type, donor, direct: PDB ID 2dui, Arg109/Glu111, indirect: PDB ID 1r34, Arg314/Gln284
N) OG atom type, donor, direct: PDB ID 4yw7, Ser72/Gly73 , indirect: PDB ID 3m6f, Ser141/Ser139
O) OG1 atom type, donor, direct: PDB ID 1gfl, Thr59/Tyr106, indirect: PDB ID 1gg9, Thr661/Pro658 **P)** O atom type, acceptor, direct: PDB ID PDB ID 2aha, Leu18/Val29, indirect: PDB ID 1jbz, Leu125/Asp19
Q) SD atom type, acceptor, direct: PDB ID 1cvk, Glu5/Met1, indirect: PDB ID 3gc7, Arg237/Met265
R) OD2 atom type, acceptor, direct: PDB ID 4p1q, Gln184/Asp155, indirect: PDB ID 2yr4, Val222/Asp231
S) OD1 atom type, acceptor, direct: PDB ID 1u5u, Ser149/Asn146, indirect: PDB ID 3vd4, His464/Asp469
T) OE2 atom type, acceptor, direct: PDB ID: 3qqj, Lys65/Glu81, indirect: PDB ID 2qwm, Val139/Glu132
U) OH atom type, acceptor, direct: PDB ID: 404b, Glu281/Tyr106, indirect: PDB ID 2iy6, Gly498/Tyr145
V) ND1 atom type, acceptor, direct: PDB ID 120l, Asp70/His31, indirect: PDB ID 2e8r, Gly240/His161

A)

B)

C)

D)

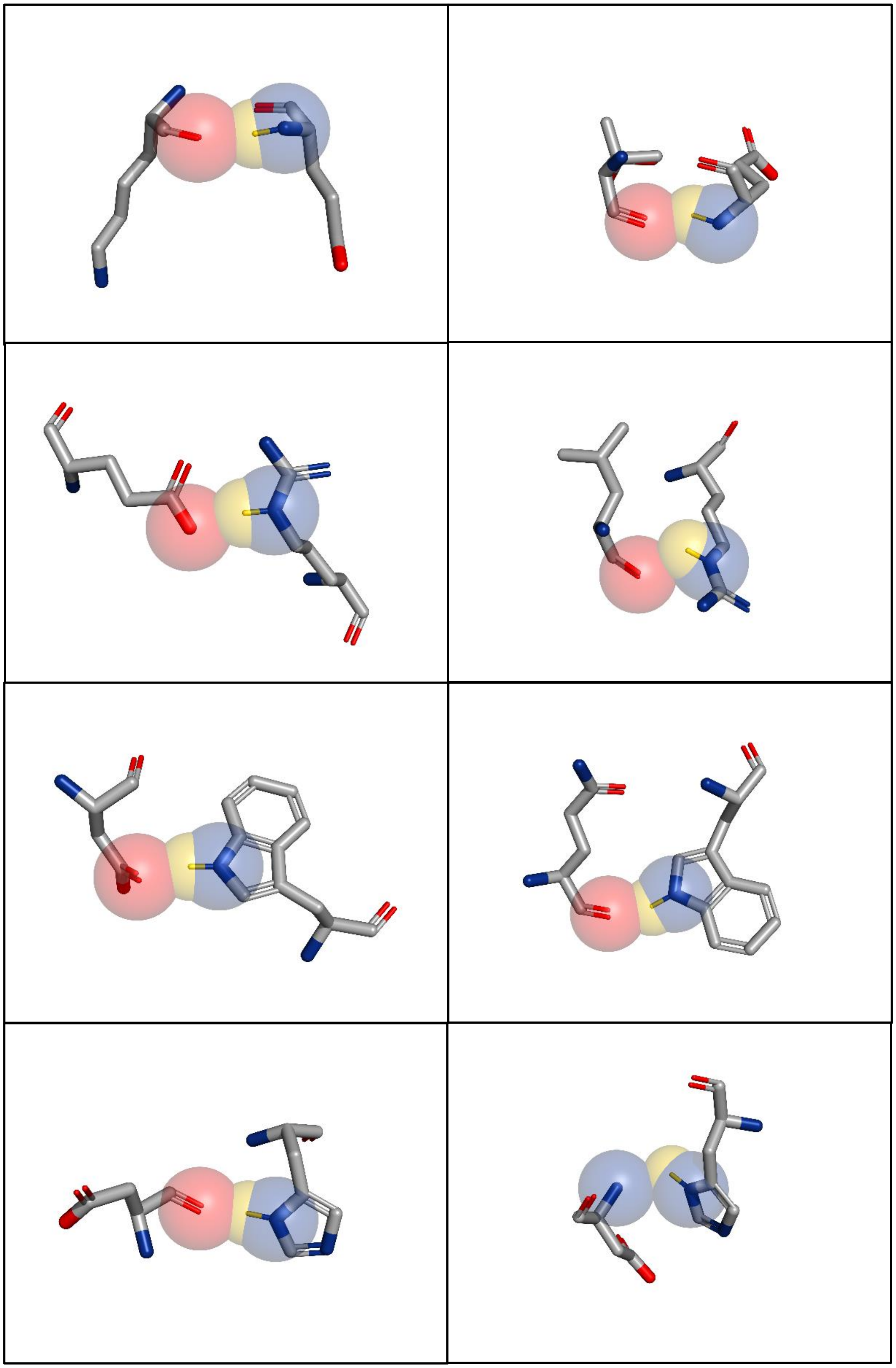

E)

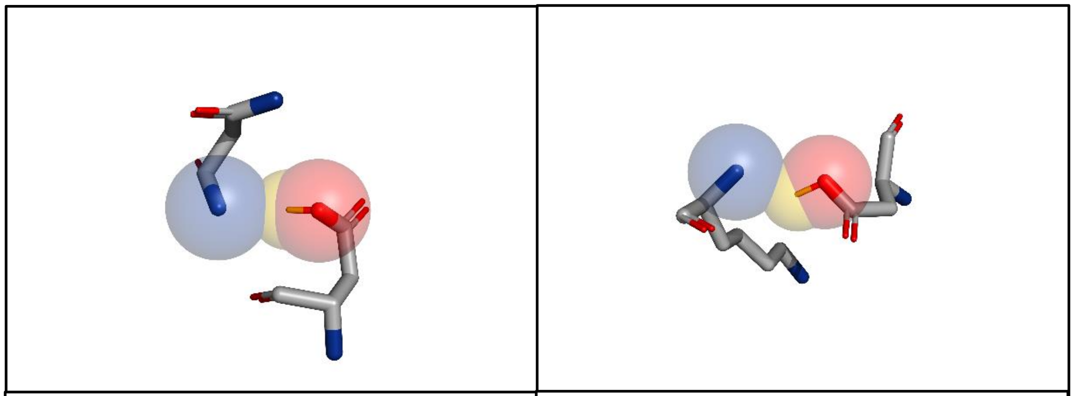

F)

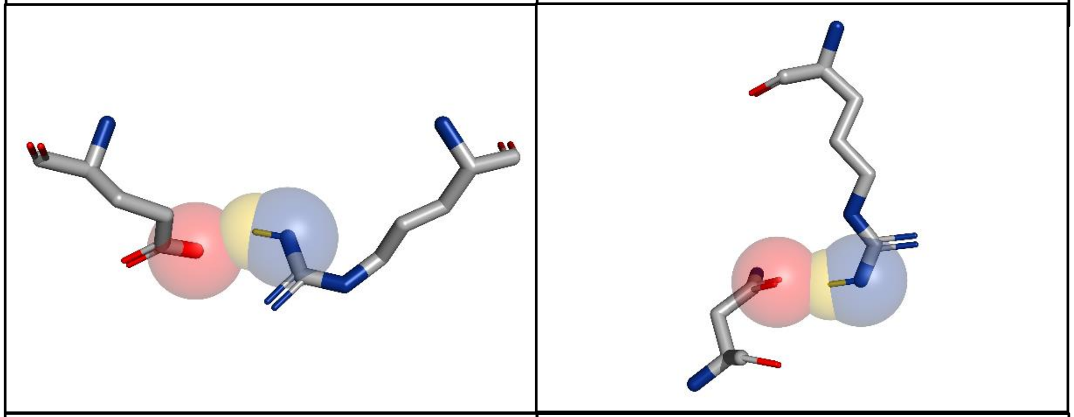

G)

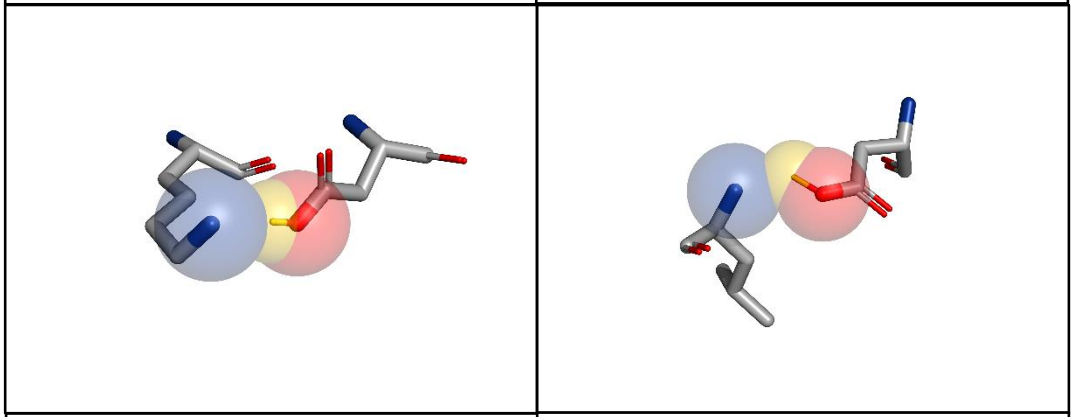

H)

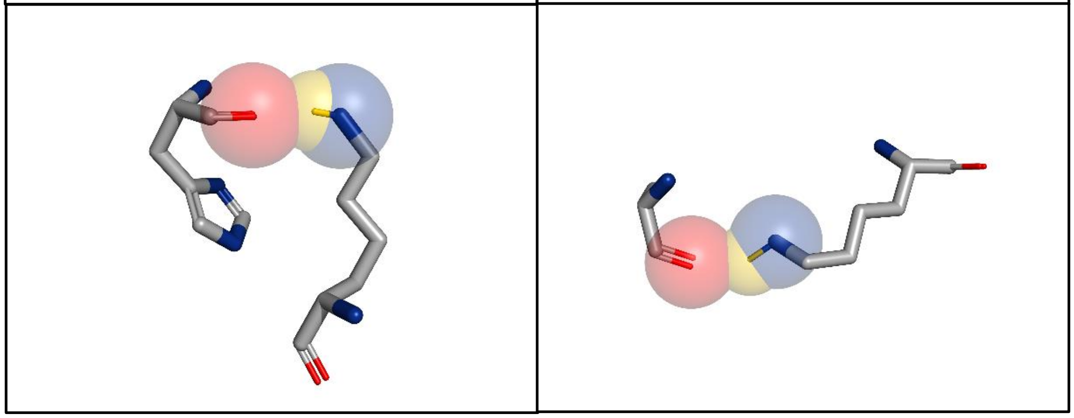

I)

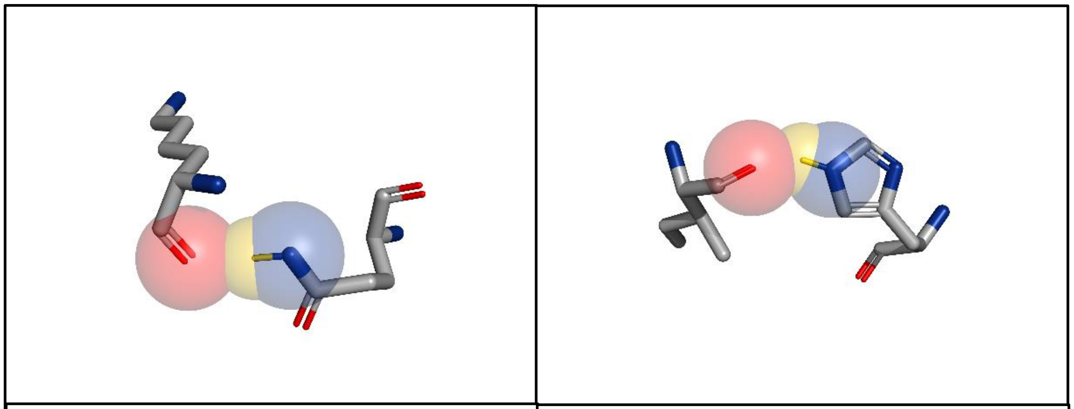

J)

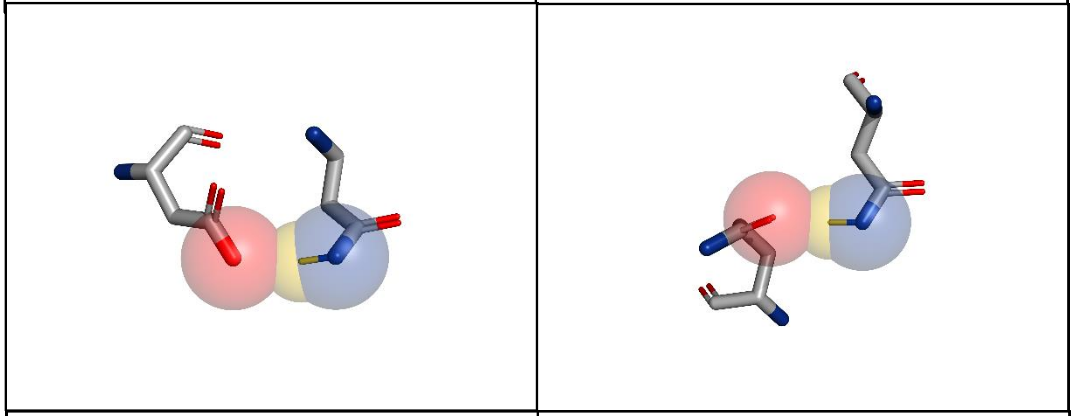

K)

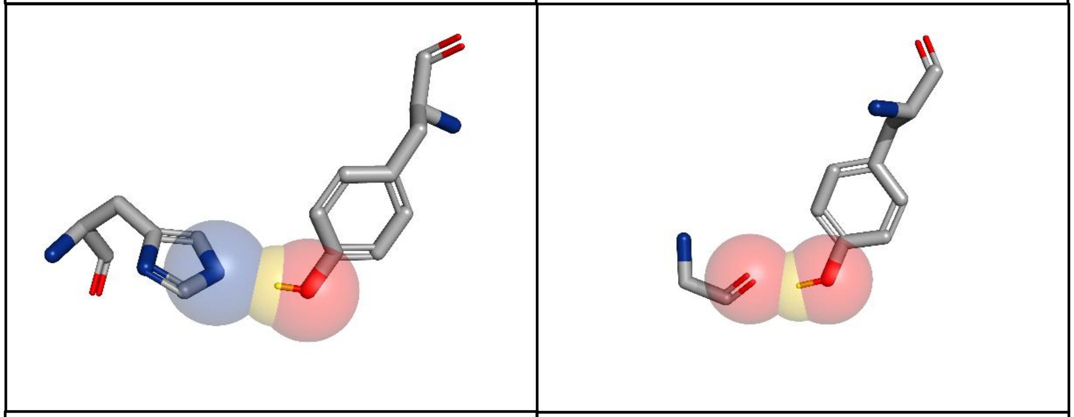

L)

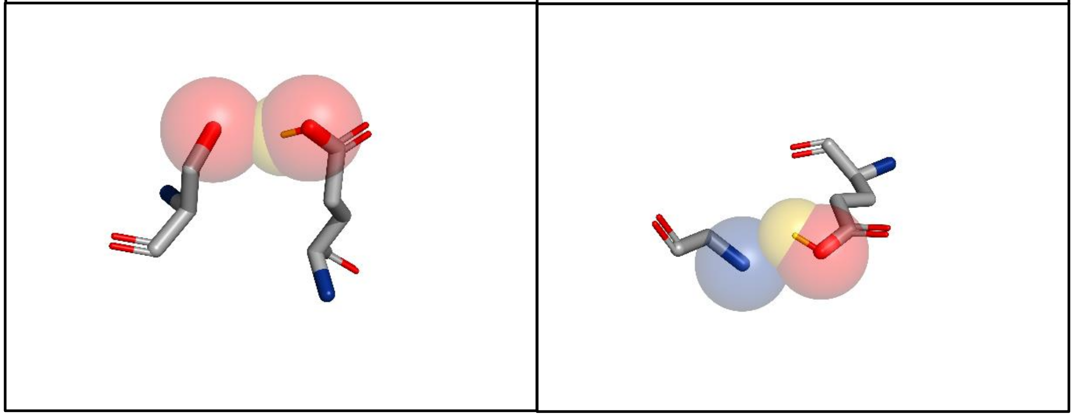

M)

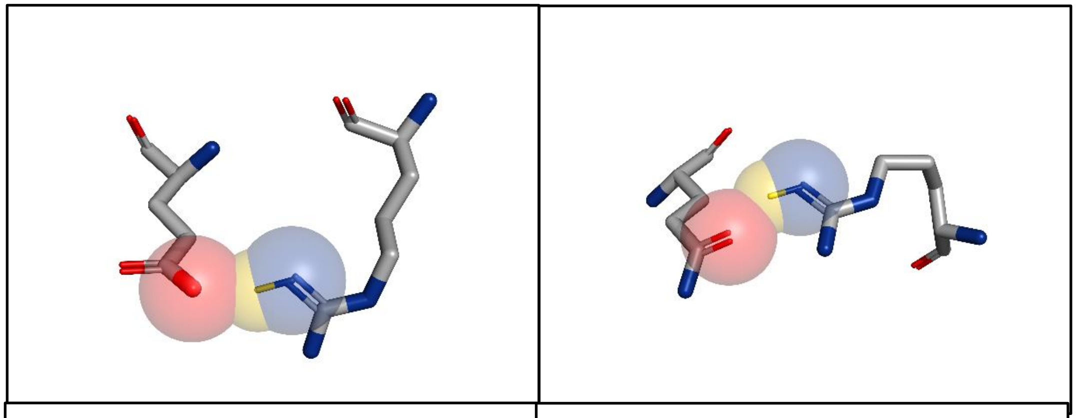

N)

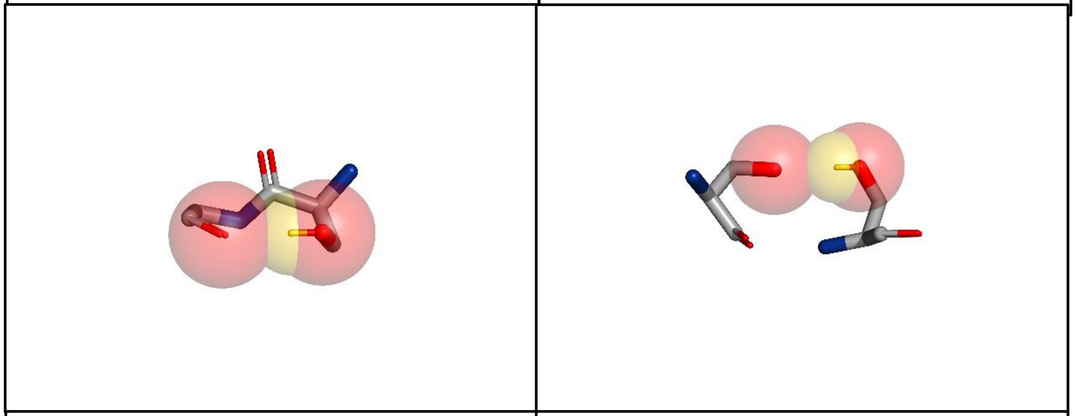

O)

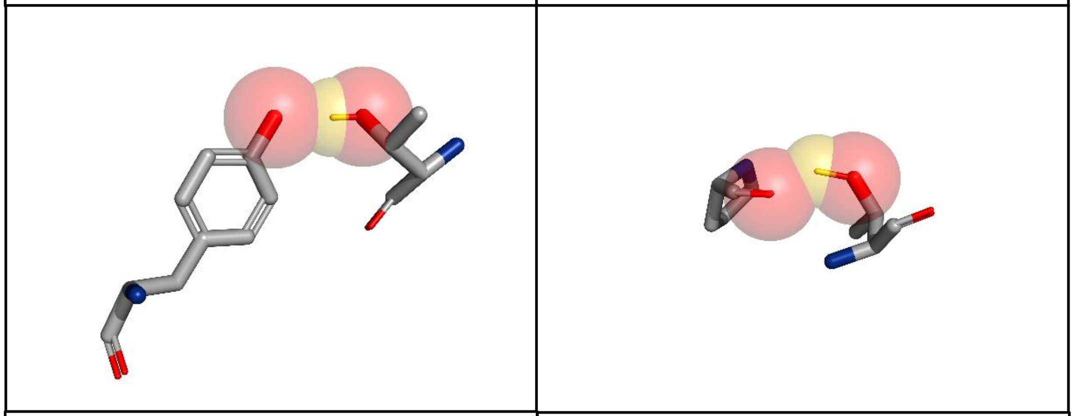

P)

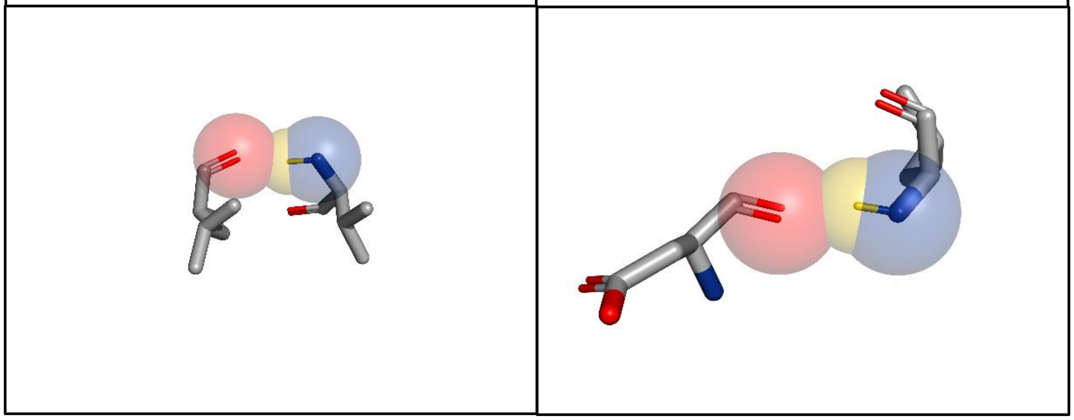

Q)

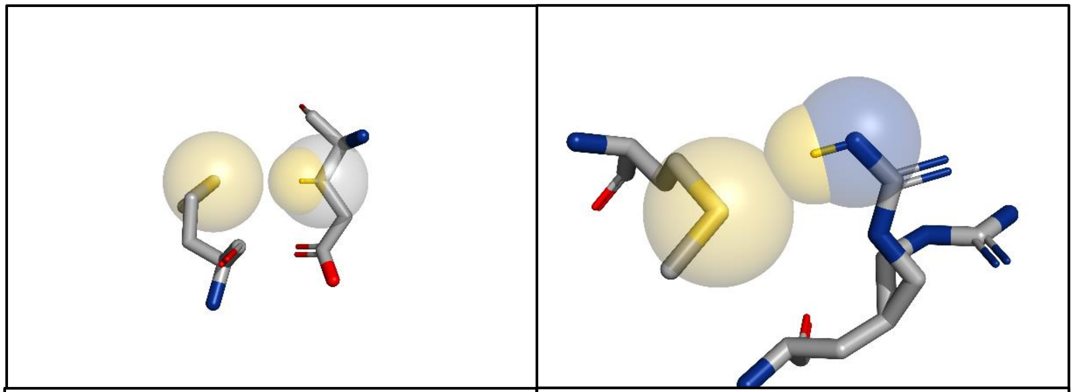

R)

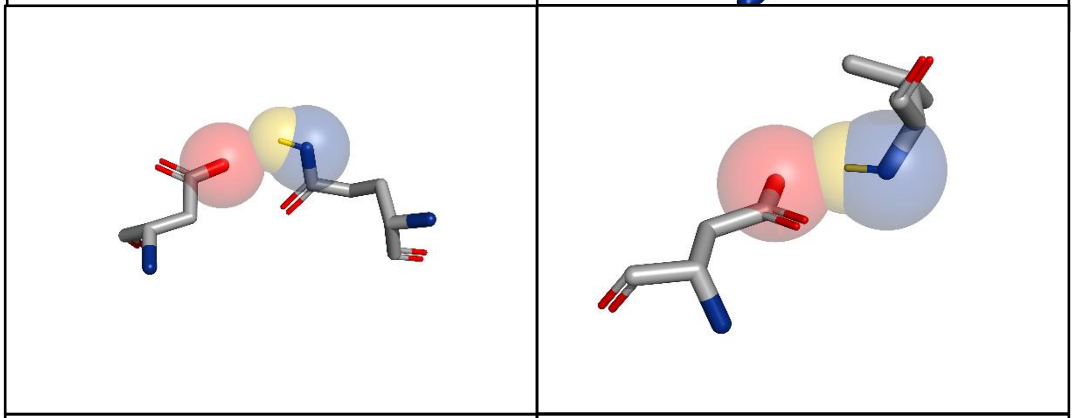

S)

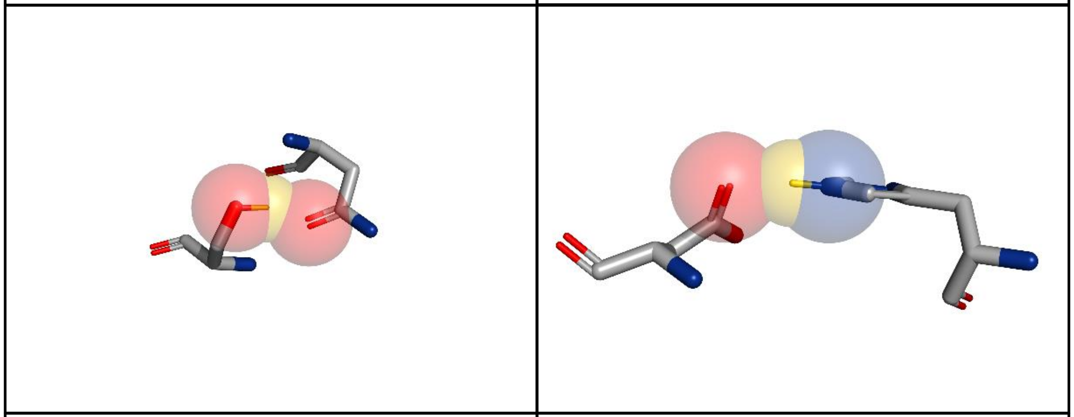

T)

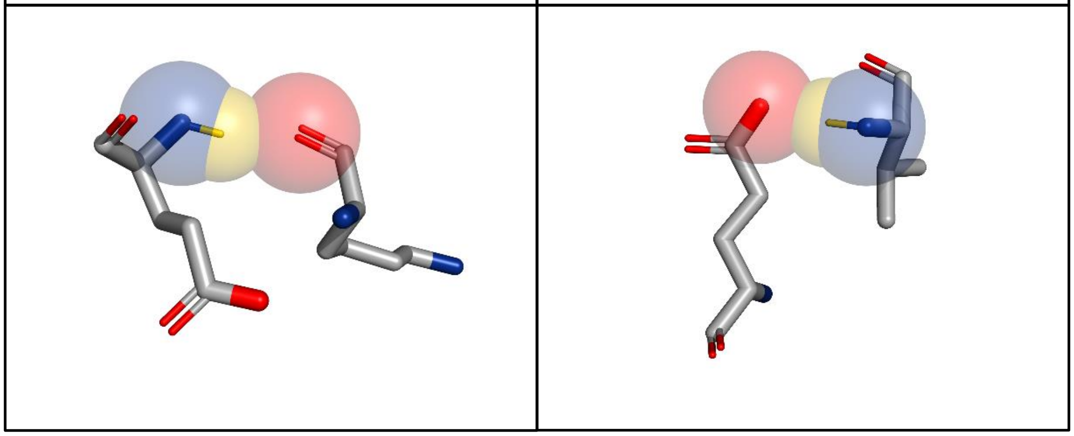

U)

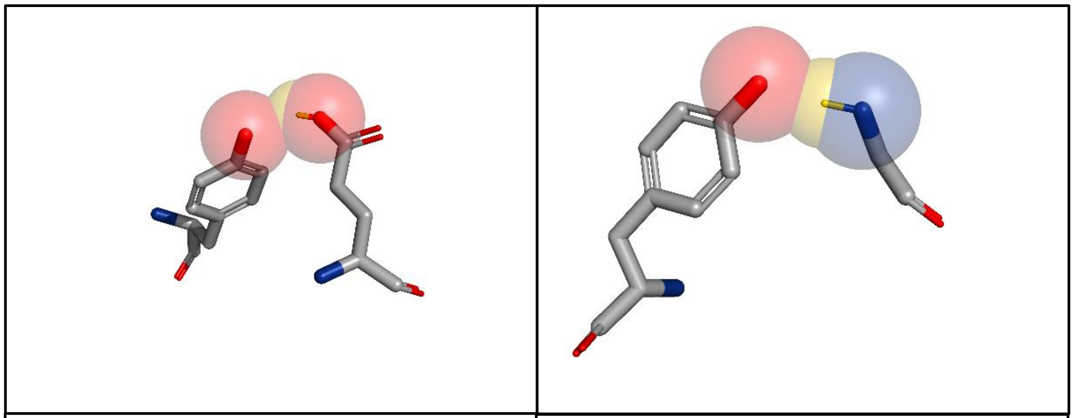

V)

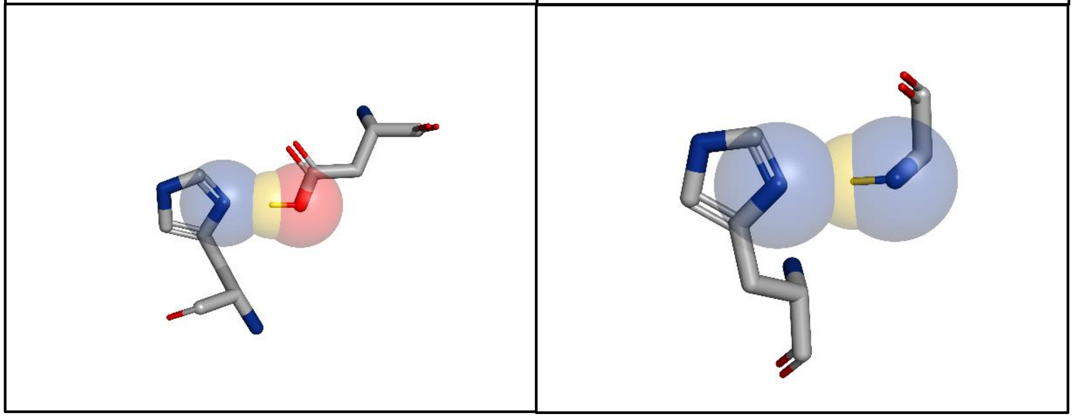

SI Figure 3: Average geometries of the identified HMI. Each identified HMI is indicated by a point corresponding to the center of the statistical peaks present in their respective heat maps. Filled in circles indicate donor atom types while open circles indicate acceptor types. Nitrogen atoms are indicated by red, oxygen by green, and sulfur in black.

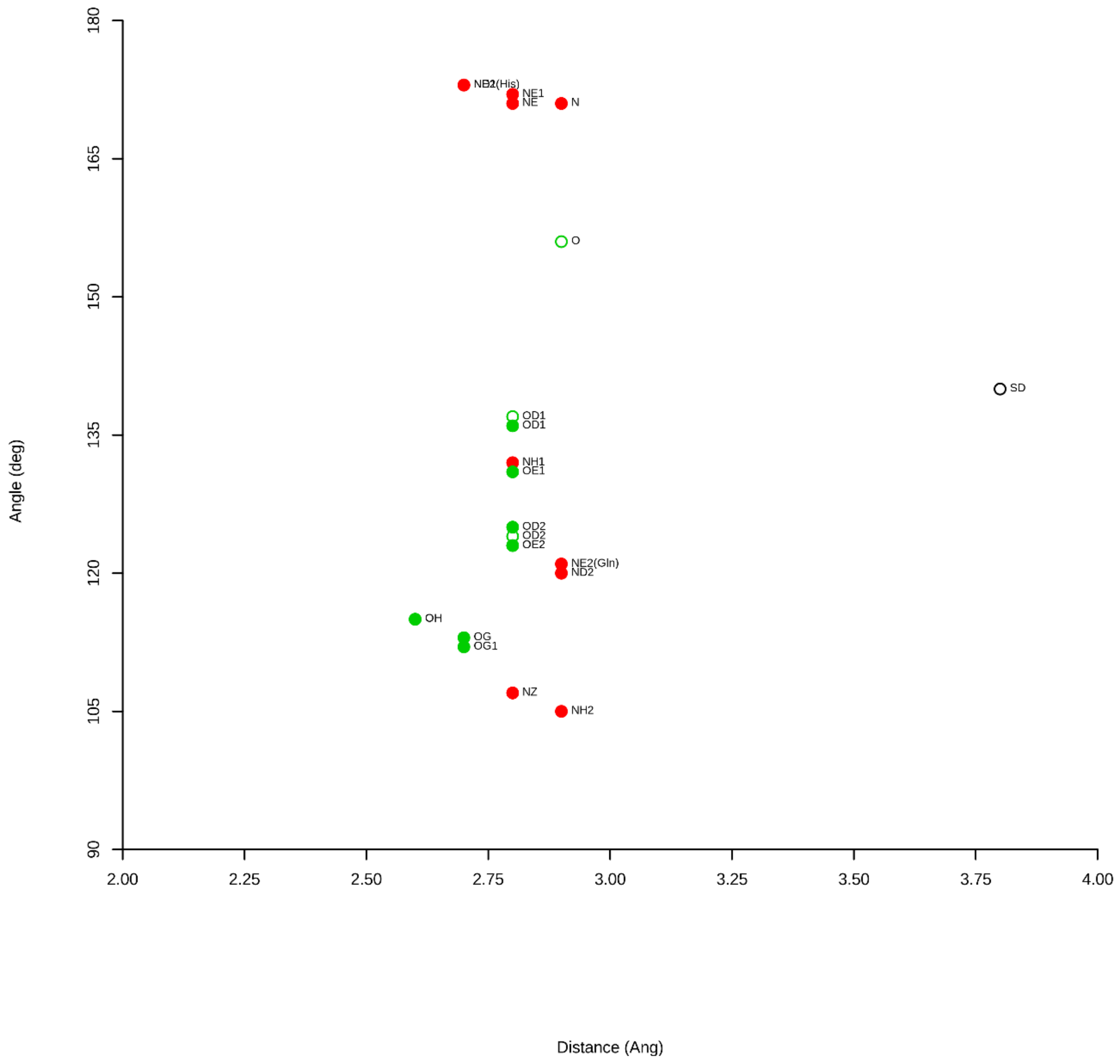

SI Figure 4: The 20 standard amino acids and their atom names from the PDB. The 20 standard amino acids in alphabetical order (by one letter code) and their atom names as in the PDB are shown. Figure made by PyMol using PDB ID 1qpb. Atoms are shown as sticks with carbon in grey, oxygen in red, nitrogen in blue, and sulfur in yellow. **A)** Alanine (Ala, A) **B)** Cysteine (Cys, C) **C)** Aspartic acid (Asp, D) **D)** Glutamic acid (Glu, E) **E)** Phenylalanine (Phe, F) **F)** Glycine (Gly, G) **G)** Histdidine (His, H) **H)** Isoleucine (Ile, I) **I)** Lysine (Lys, K) **J)** Leucine (Leu, L) **K)** Methionine (Met, M) **L)** Asparagine (Asn, N) **M)** Proline (Pro, P) **N)** Glutamine (Gln, Q) **O)** Arginine (Arg, R) **P)** Serine (Ser, S) **Q)** Threonine (Thr, T) **R)** Valine (Val, V) **S)** Tryptophan (Trp, W) **T)** Tyrosine (Tyr, Y)

A)

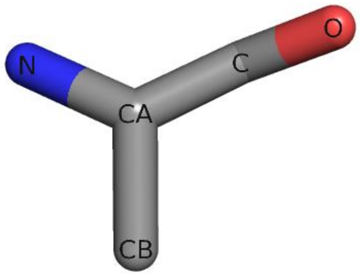

B)

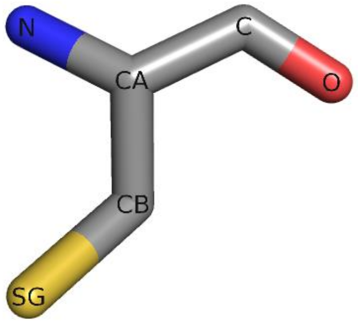

C)

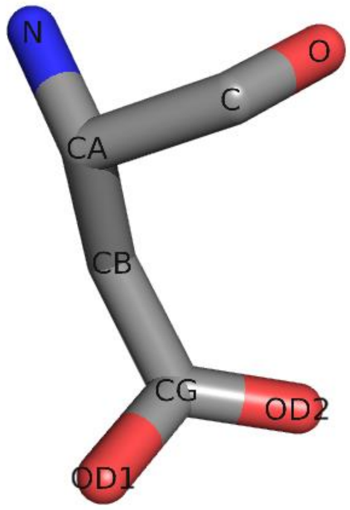

D)

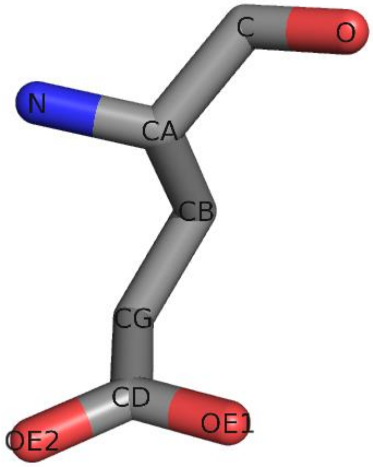

E)

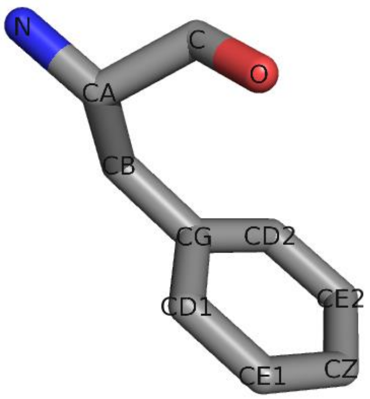

F)

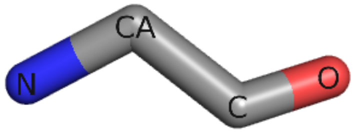

G)

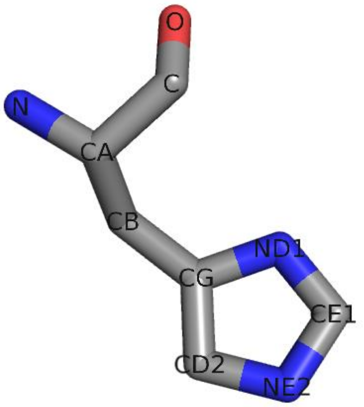
O
C
N
CA
CB
ND1
CG
CE1
CD2
NE2

H)

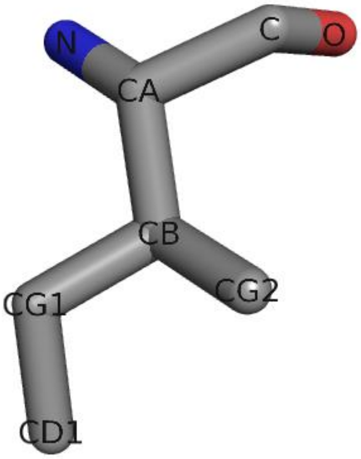
N
C
O
CA
CB
CG1
CG2
CD1

I)

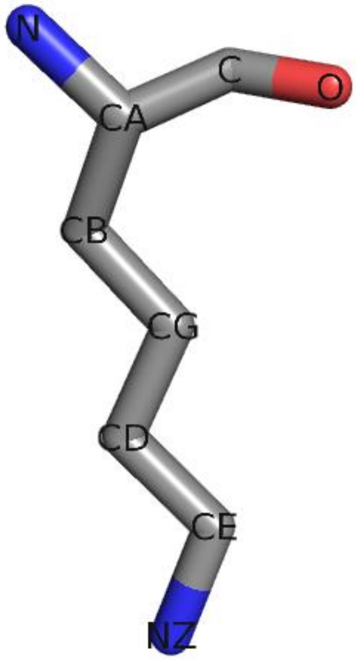
N
C
O
CA
CB
CG
CD
CE
NZ

J)

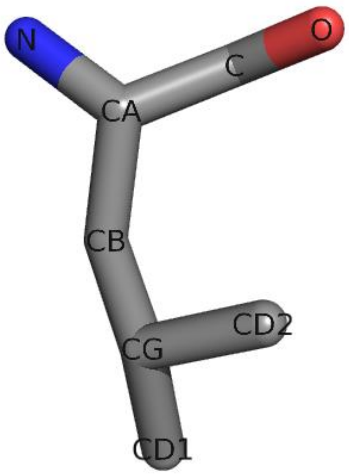
N
O
C
CA
CB
CD2
CG
CD1

K)

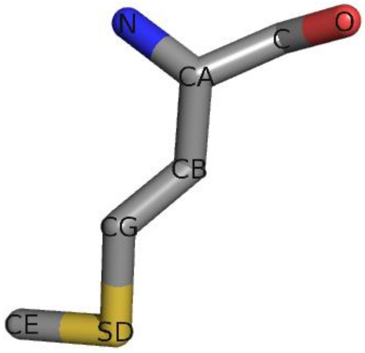
N
O
C
CA
CB
CG
CE
SD

L)

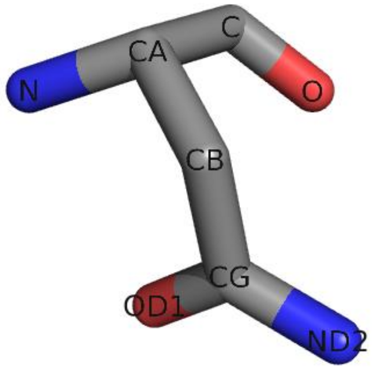
C
CA
N
O
CB
CG
OD1
ND2

M)

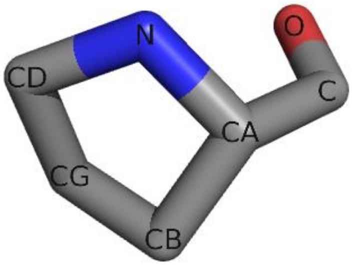
N
O
CD
C
CA
CG
CB

N)

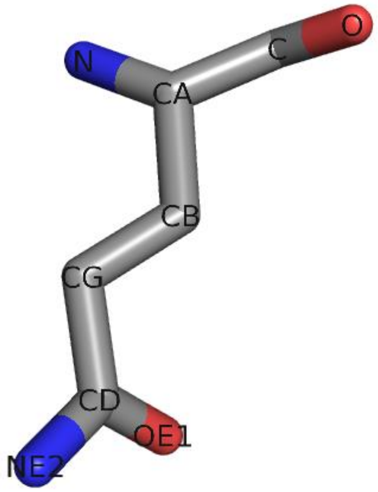
O
N
C
CA
CB
CG
CD
OE1
NE2

O)

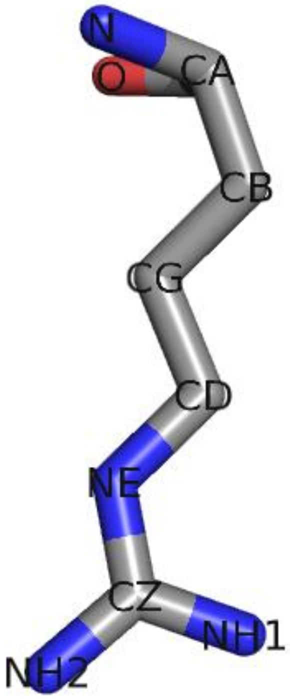
N
O
CA
CB
CG
CD
NE
CZ
NH1
NH2

P)

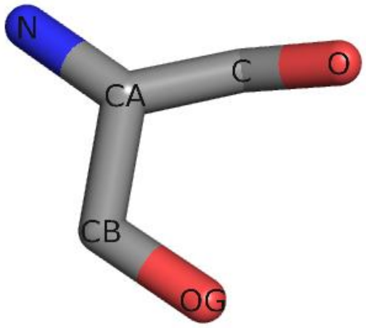
N
C
O
CA
CB
OG

Q)

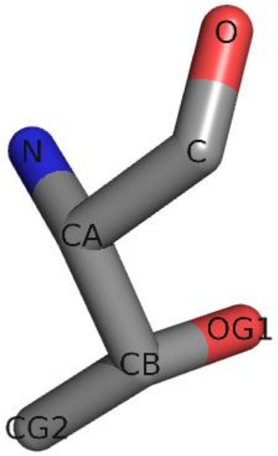
O
N
C
CA
OG1
CB
CG2

R)

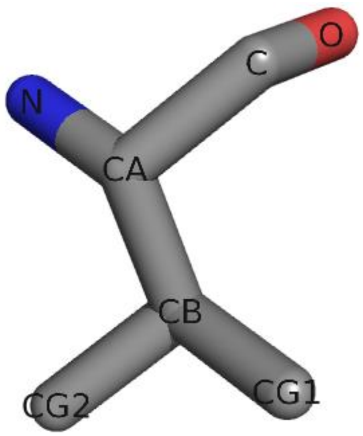
O
C
N
CA
CB
CG2
CG1

S)

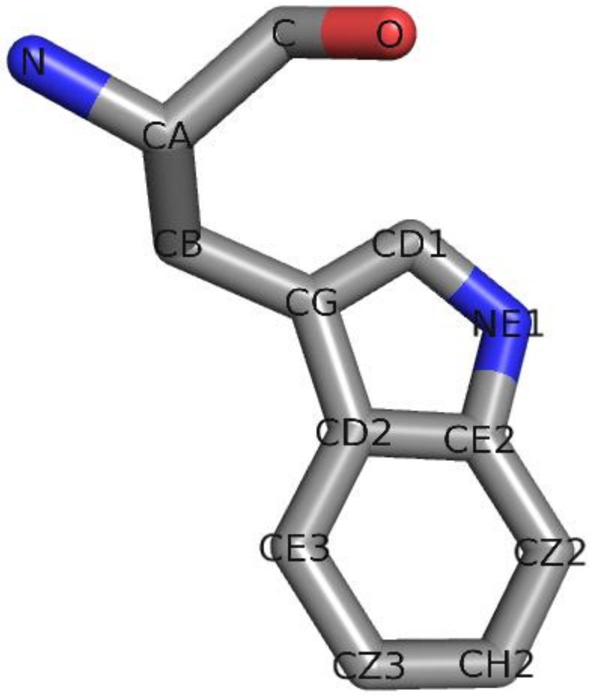
N
C
O
CA
CB
CD1
CG
NE1
CD2
CE2
CE3
CZ2
CZ3
CH2

T)

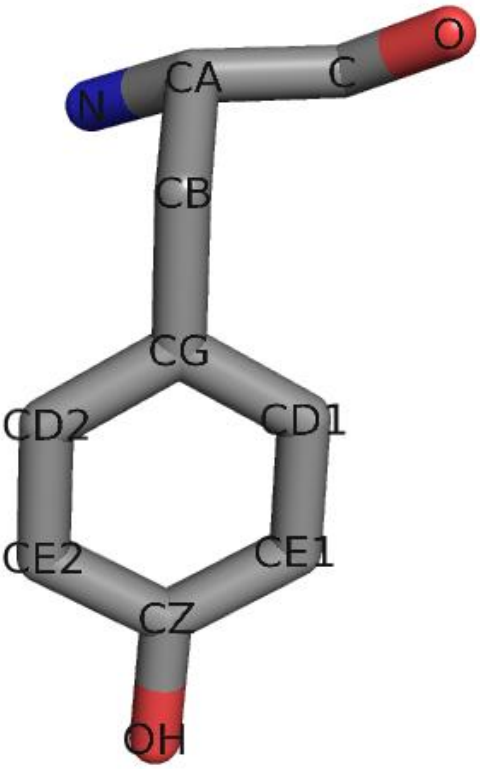
O
CA
C
N
CB
CG
CD2
CD1
CE2
CE1
CZ
OH
